# Supplementary material for: The impact of inversions across 33,924 families with rare disease from a national genome sequencing project
Source: Am J Hum Genet. 2024 May 21;111(6):1140–64. doi: 10.1016/j.ajhg.2024.04.018 (PMC11179413; doi:10.1016/j.ajhg.2024.04.018)
Supplement: Document S1. Figures S1–S31, Tables S3 and S4, and Notes S1–S8 [file mmc1.pdf]

The American Journal of Human Genetics, Volume *111*

Supplemental information

The impact of inversions across 33,924 families with rare disease from a national genome sequencing project

Alistair T. Pagnamenta, Jing Yu, Susan Walker, Alexandra J. Noble, Jenny Lord, Prasun Dutta, Mona Hashim, Carme Camps, Hannah Green, Smrithi Devaiah, Lina Nashef, Jason Parr, Carl Fratter, Rana Ibnouf Hussein, Sarah J. Lindsay, Fiona Lalloo, Benito Banos-Pinero, David Evans, Lucy Mallin, Adrian Waite, Julie Evans, Andrew Newman, Zoe Allen, Cristina Perez-Becerril, Gavin Ryan, Rachel Hart, John Taylor, Tina Bedenham, Emma Clement, Ed Blair, Eleanor Hay, Francesca Forzano, Jenny Higgs, Natalie Canham, Anirban Majumdar, Meriel McEntagart, Nayana Lahiri, Helen Stewart, Sarah Smithson, Eduardo Calpena, Adam Jackson, Siddharth Banka, Hannah Titheradge, Ruth McGowan, Julia Rankin, Charles Shaw-Smith, D. Gareth Evans, George J. Burghel, Miriam J. Smith, Emily Anderson, Rajesh Madhu, Helen Firth, Sian Ellard, Paul Brennan, Claire Anderson, Doug Taupin, Mark T. Rogers, Jackie A. Cook, Miranda Durkie, James E. East, Darren Fowler, Louise Wilson, Rebecca Igbokwe, Alice Gardham, Ian Tomlinson, Diana Baralle, Holm H. Uhlig, and Jenny C. Taylor

Note S1: Additional background on MLPA testing.

In addition to array-based testing for genome-wide copy number alterations (CNV), multiplex ligation-dependent probe amplification (MLPA) is another technology commonly used in clinical testing laboratories. This targeted method is based on a ligation reaction, followed by multiplex PCR, with typically up to 40 probes in any one mix. The method is suitable for conditions where there are a small number of strong candidate genes. Although dependant on probe design, single exon CNVs can be picked up robustly. In some cases, where the precise nature of the rearrangement is known, probes that target copy-neutral SVs can also be incorporated. For instance, a common 10Mb inversion involving *MSH2*[1] is captured by the latest commercial MLPA product (e.g. SALSA probemix for MLH1/MSH2, P003-D1; MRC Holland). However, this remains an exception rather than the rule, and the vast majority of balanced rearrangements will be missed by MLPA, as well as by arrays.

Note S2: Additional background about the 100,000 Genomes Project.

The 100kGP is a national genome sequencing study run by Genomics England, a company owned by the Department of Health and Social Care. The project was initiated in 2012 and most of the sequencing was completed in 2018. Although many thousands of primary/secondary results have already been returned to participants, analysis of data is ongoing through Genomics England's diagnostic discovery route. There have now been over 1,000 approved research projects utilizing this data (https://research.genomicsengland.co.uk/research-registry), and over 1,100 researchers active within the framework of the National Genomic Research Library. Building on the success of the 100kGP, genome sequencing is now being offered routinely within the NHS for a wide range of clinical indications. In the main RD programme of the 100kGP, the diagnostic rate is estimated to be 20-25%, and varies according to the clinical indication, which further highlights the importance of improving variant detection/prioritisation strategies in the clinical analysis pipeline. The analysis described in this study is covered by project RR693 in the Genomics England Research Registry ("The impact of germline inversions in the rare disease arm of the 100,000 Genomes Project") which was submitted in March 2022 and has been approved by Genomics England. The majority of variants reported here were entered into the Diagnostic Discovery pathway over a 9 month period between September 2021 and June 2022.

Note S3: Additional example of individual with phenotype blending.

In Family 12, the proband was already known to have achondroplasia and prior testing of *FGFR3* had uncovered a maternal NM_000142.5:c.1138G>A, p.(Gly380Arg) variant. This is a well-known recurrent pathogenic mutation listed in ClinVar with over 40 submissions (VCV000016327.104). This family had been recruited to the 100kGP as it was felt that *FGFR3* did not explain all the individual's clinical features. Identification of a complex SV in *EFTUD2* led to the hypothesis that this variant may be acting together with the missense in *FGFR3* to result in the participant's phenotype. The SV in *EFTUD2* was shown to have arisen *de novo* and comprised a deletion of 6.5kb, with two retained internal segments of 252bp and 236bp (Figure S12).

Note S4: Enzyme and immunohistochemistry for *PDHA1.*

In Family 41, an inversion call involving exons 6-9 of *PDHA1* (NM_000284.4) was in fact the proximal 3x end of a duplication-triplication (Figure S5). This SV was identified in a 100kGP participant with exercise intolerance, intellectual disability and white matter abnormalities and so compatible with Pyruvate dehydrogenase E1-alpha deficiency [MIM #312170]. Interpretation was more complex due to the structural ambiguity. This SV had previously been picked up independently by a clinical laboratory using array-CGH and interpreted as a duplication of uncertain significance. Pyruvate

dehydrogenase activity was measured in cultured fibroblasts from the proband and the mean activity of 0.54 nmol/mg protein/min was marginally below the normal range of 0.6-0.9. In a female, there is always the possibility of normal, or near normal, activity with heterozygosity for a *PDHA1* mutation and a pattern of X-inactivation favouring expression of the normal X chromosome, however, this is relatively uncommon. In light of the Xp22 rearrangement in this patient, cells were also analysed with an antibody to the Ela subunit to see if there was any evidence of mosaicism. This method permits small populations of deficient cells to be detected. However, the cells were all uniformly positive with the antibody. Together with the near-normal enzyme activity, these results suggest that the duplication likely has no consequences as far as *PDHA1* is concerned.

Note S5: Examples from the 100kGP of complex SVs in autosomal recessive disease associated genes.

We recently described a 100kGP participant with generalized arterial calcification of infancy [MIM #208000] who harboured interlinked/inverted duplications that disrupt *ENPP1*.[2] The variant was identified following a manual search at a specific locus that was prompted by clinical suspicion. Another (unpublished) example from the 100kGP involves a previously reported complex deletion-inversion involving *OCA2*[3] found *in trans* with NM_000275.3:c.1441G>A (p.Ala481Thr, VCV000000954.45) in sisters with developmental macular and foveal dystrophy. In that case, the SV was identified via use of a SV-haplotype tagging SNV (rs374519281).

Note S6: Additional examples of cryptic *APC* variants.

Inversions that disrupt *APC* have been reported previously and these have been detected using a variety of methods such as by high coverage NGS capture of intronic regions[4] or by nanopore sequencing.[5] An earlier study also used a cDNA approach to detect structural variants in 4/49 potential FAP families,[6] suggesting that SVs involving this gene are not uncommon. Another study identified two individuals with adenomatous polyposis likely due to intronic SVA element insertions that affect *APC*.[7] Other recent reports highlight that genome sequencing can detect deep intronic variants that lead to the introduction of pseudoexons in the *APC* transcript.[8]

Note S7: Other examples of complex SVs reported with incomplete interpretation.

In a recent study on inherited eye diseases[9], Fig. 3 shows a complex deletion-inverted non-tandem duplication in *EYS*. Careful review of the images shown suggests that the authors interpretation may be incomplete. Due to the presence of a 31kb duplication which cannot be spanned with short reads, the middle “Segment C” could be both ways around and the split read pattern would be identical. In addition, the extra copy of exon 31 is on the other strand from the rest of the gene and we suspect is unlikely to be spliced into the RNA transcript.

In another recent report, a rearrangement disrupting *SMARCAL1*, found *in trans* with a frameshift variant in a patient with Schimke immune-osseous dysplasia, was interpreted as inversion.[10] Again, closer scrutiny of the published IGV image suggests that a non-tandem duplication inserted in an inverted orientation could also potentially explain that short-read data.

Note S8: Additional tips on manual review of read alignments.

Read alignments files (BAM or CRAM format) can be loaded up for manual analysis using IGV software that is freely available (https://igv.org/doc/desktop). When viewing read alignments, it is important to use the IGV setting “color alignments by insert size and pair orientation” or the more stringent “color alignments by pair orientation” such that +ve to +ve strand read-pair mappings are highlighted in teal, whilst -ve to -ve mappings are in blue. For balanced SVs, the green and blue should point inwards towards a discreet breakpoint. Where copy number changes are involved, split-reads should

point to the breakpoint but only in the direction going from higher to lower coverage. For large genes, it can also be helpful to load up structural vcf file in IGV as well, to help guide the analyst towards which regions of the gene are most critical to check in more detail. The “show mismatched bases” option can as also be turned off to further assist visualisation of SVs in read alignment data. We hope that the collection of IGV screenshots provided here, in combination with access to alignment data via the National Genomic Research Library, can be a useful learning resource for genome analysts new to structural variation.

Visualisation is critical to help facilitate the correct interpretation of complex SVs and many ways to illustrate such rearrangements have been proposed. Schematic diagrams showing the relative copy number states and the positions/directions of the split read-pairs were crucial in several cases for determining if additional configurations were potential solutions to the short-read data. For Family 43, we felt that instead of Circos plot, a “subway” plot gave a better representation and showed in an intuitive way that the complex SV structure was a translocation that could be confirmed by karyotyping, which proved to be correct. In contrast, for the cases involving *MECP2,* annotated dotplots generated from single PacBio reads were used to help demonstrate the precise configuration of the rearrangement and aid interpretation. This worked well for Family 33 (Figure 4B) and comparison to a similar plot produced for Family 47 (Figure S28) helped confirm that the same hotspot region was involved. We anticipate that future developments in this area should help automate SV reconstruction (i.e. variant calling algorithms that report complete structures, not just breakpoints) and aid conceptualisation of complex SVs.

Supplemental Figures

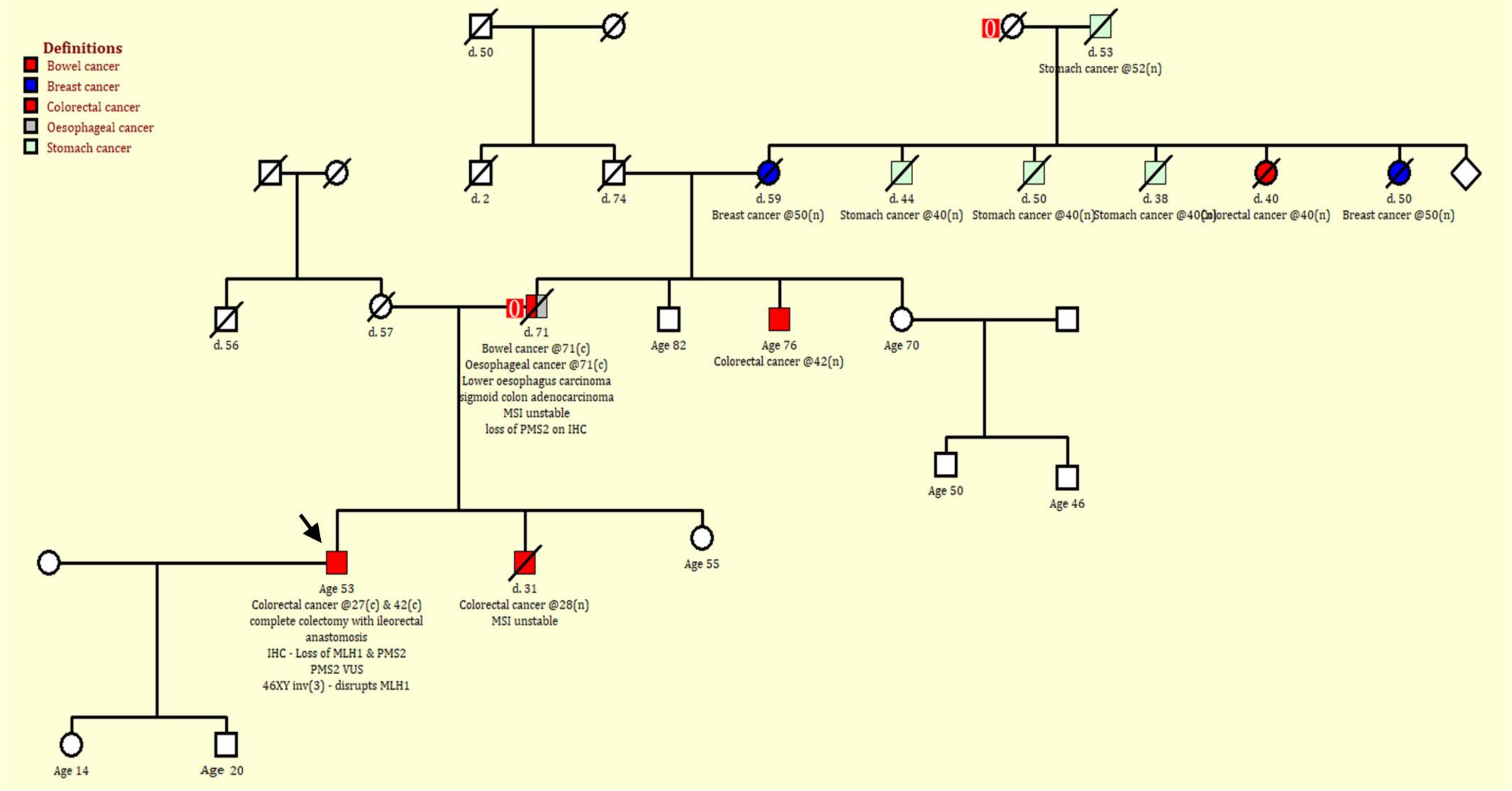

Figure S1: Pedigree for Family 19 harbouring a 30.7Mb inversion that disrupts *MLH1*. The 53 year old male labelled with the arrow is the 100k Genomes Project participant.

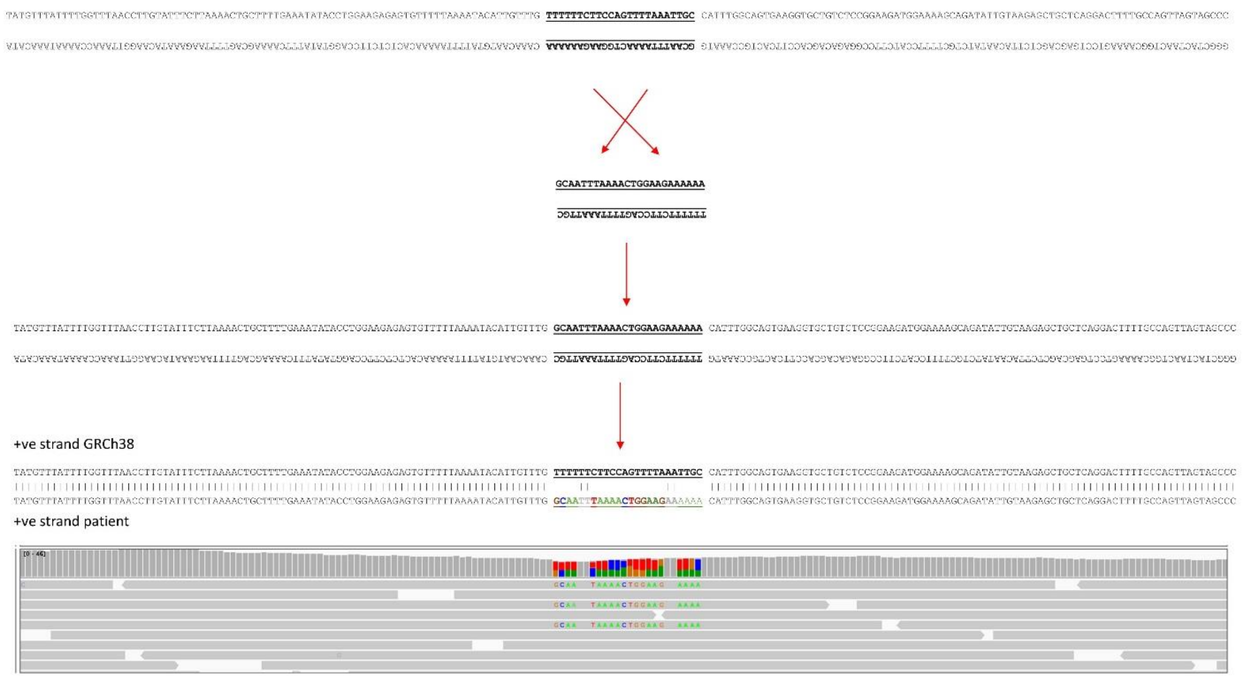

Figure S2: Schematic diagram illustrating how the 24bp inversion seen in Family 45 can result in the pattern of mismatches seen in the read alignments shown in IGV. For 4 positions, the inversion does not change the DNA base present.

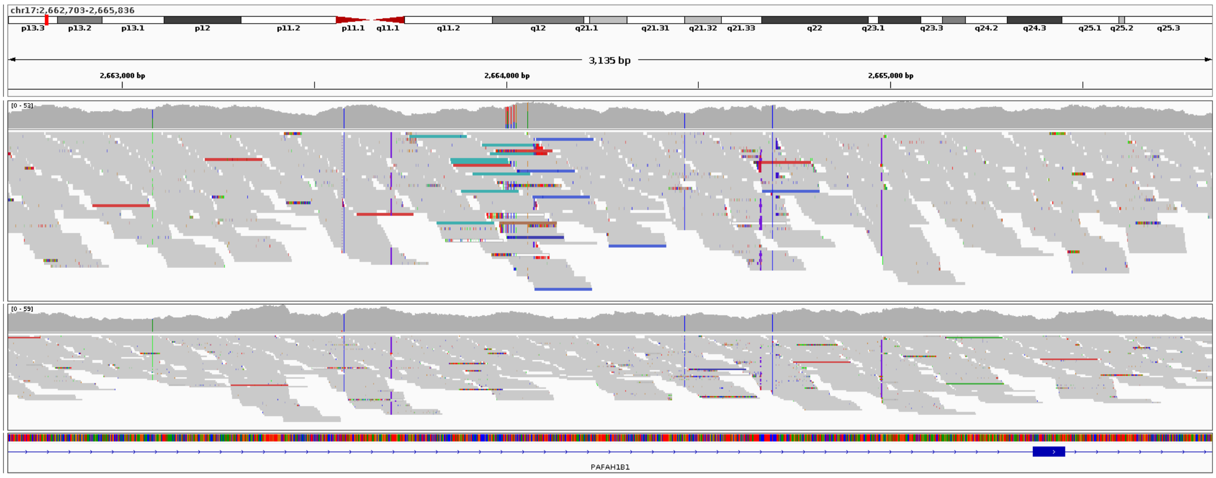

Figure S3: Read alignments supporting a 256kb inversion involving *PAFAH1B1* (NM_000430.4) in Family 13. Split read-pairs shown in blue (-ve to -ve strand) and teal (+ve to +ve strand) are seen for the proband (upper) but not in the mother (lower). As the proximal breakpoint lies in intron 2, this inversion is likely to disrupt gene function. The high degree of phenotypic specificity lends additional weight supporting this inversion to be responsible for the patient's diagnosis.

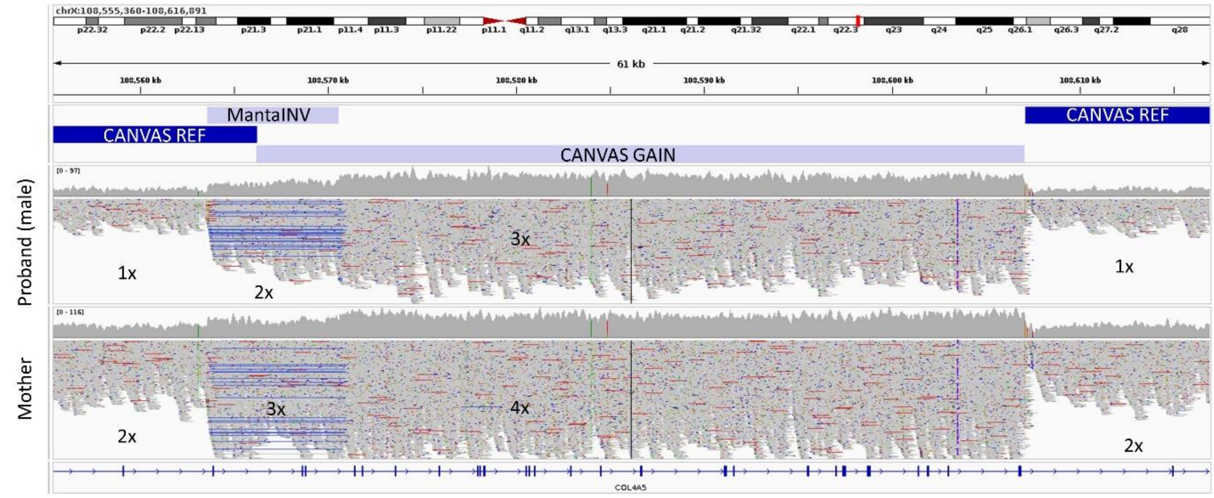

Figure S4: Read alignments supporting complex duplication-triplication involving *COL4A5* in Family 25. The 7kb MantaINV call involving 3 coding exons is shown as thin horizontal blue lines which denote -ve to -ve strand mapping split read pairs. Reads are shown using the squished view option in IGV. The algorithmic SV calls are shown in the top track and the relative copy number states are labelled. The rearrangement was present in the similarly affected mother (lower track).

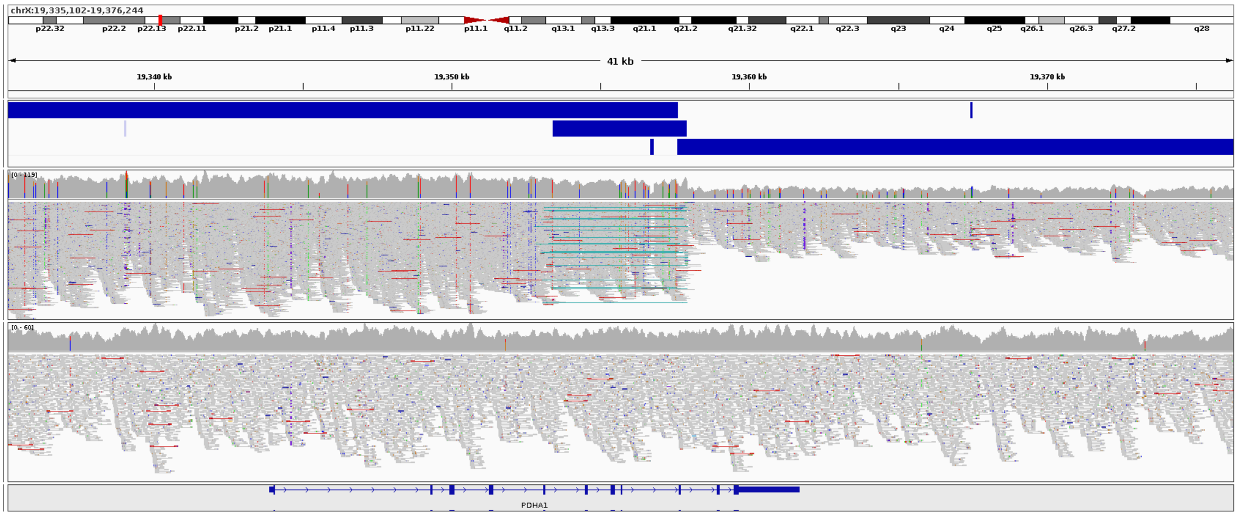

Figure S5: Read alignments supporting complex duplication-triplication involving *PDHA1* in Family 41. The 4.5kb MantaINV call involving 4 coding exons is shown as a horizontal blue bar in the upper track. Read alignments for the proband highlight +ve to +ve strand read pairs (in teal) which, combined with the increased coverage, are indicative of a duplication-triplication. The rearrangement was not seen in the unaffected mother (lower track). Testing of the father was not possible.

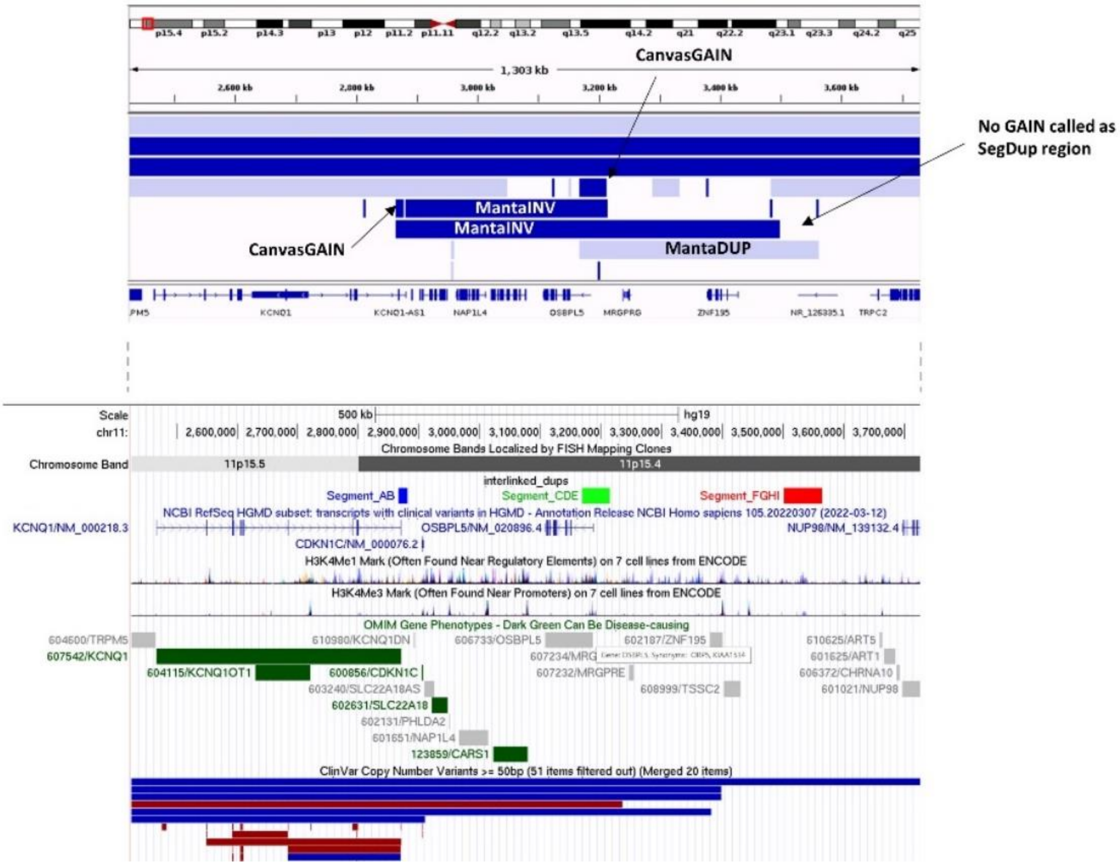

Figure S6: Complex *de novo* SV in Family 44 involving three interlinked duplications on chromosome 11p15.4. The SV was identified due to two overlapping MantaINV calls, as shown in the IGV screenshot. The genomic window shown in IGV is aligned to the UCSC genome browser image below. The smallest of the duplicated segments (AB; blue) lies 24kb downstream of *CDKN1C* and also close to *KCNQ1OT1* (KCNQ1-opposite strand/antisense transcript 1) which has a critical role in regulating *CDKN1C*. An interactive UCSC session is available at http://genome.ucsc.edu/s/AlistairP/CDKN1C_duplications. Coordinates shown here are based on GRCh37, but are lifted over to GRCh38 for Table S2.

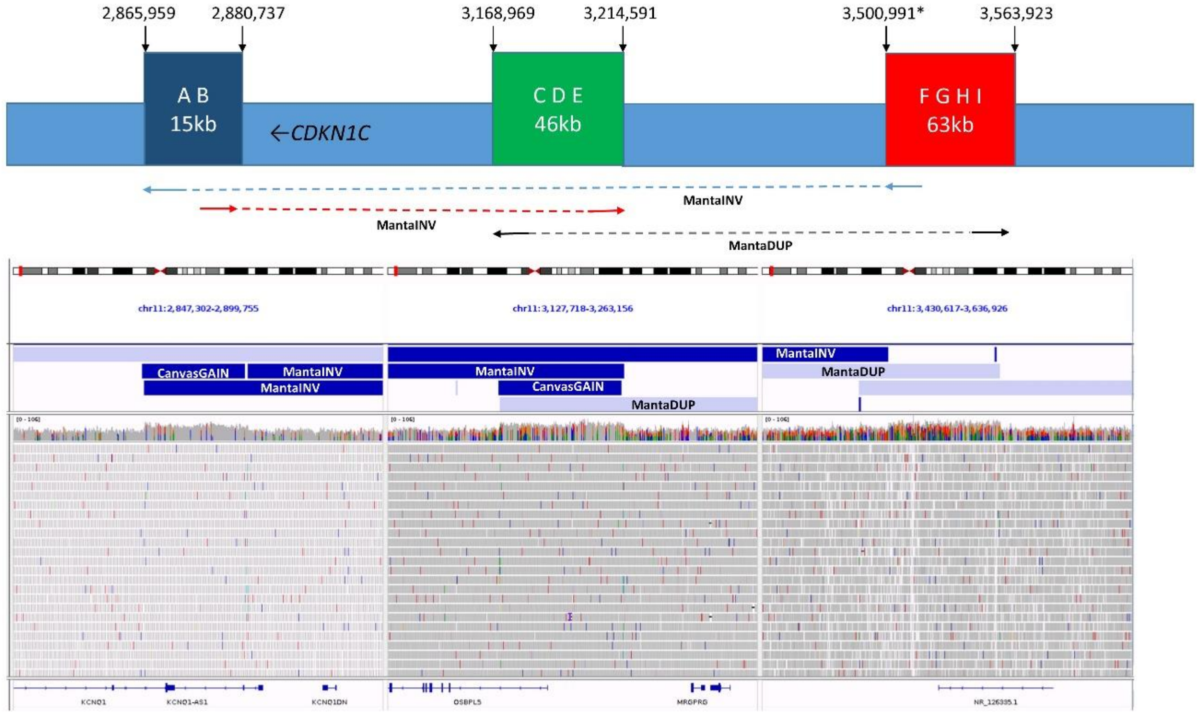

Figure S7: Summary of split reads for complex *de novo* SV found in Family 44. The three duplications on chromosome 11p15.4 are interlinked and this resulted in 2 MantaINV and 1 MantaDUP call. Two of the three duplications were called by CANVAS but the largest ~63kb was missed due to the presence of a SegDUP and low mapping quality. Positions shown are based on GRCh37.

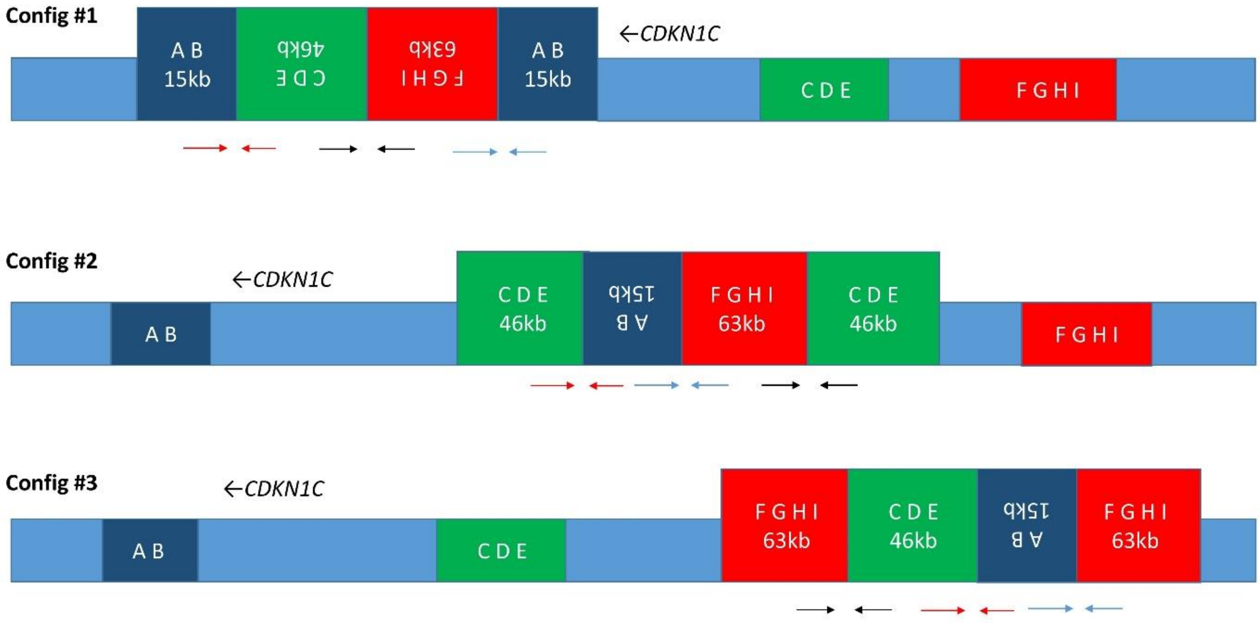

Figure S8: Schematic diagram of complex *de novo* SV found in Family 44. Short read data is ambiguous as there are three possible SV configurations that could potentially explain the split-read data. Approximate segment sizes are indicated, but not drawn to scale.

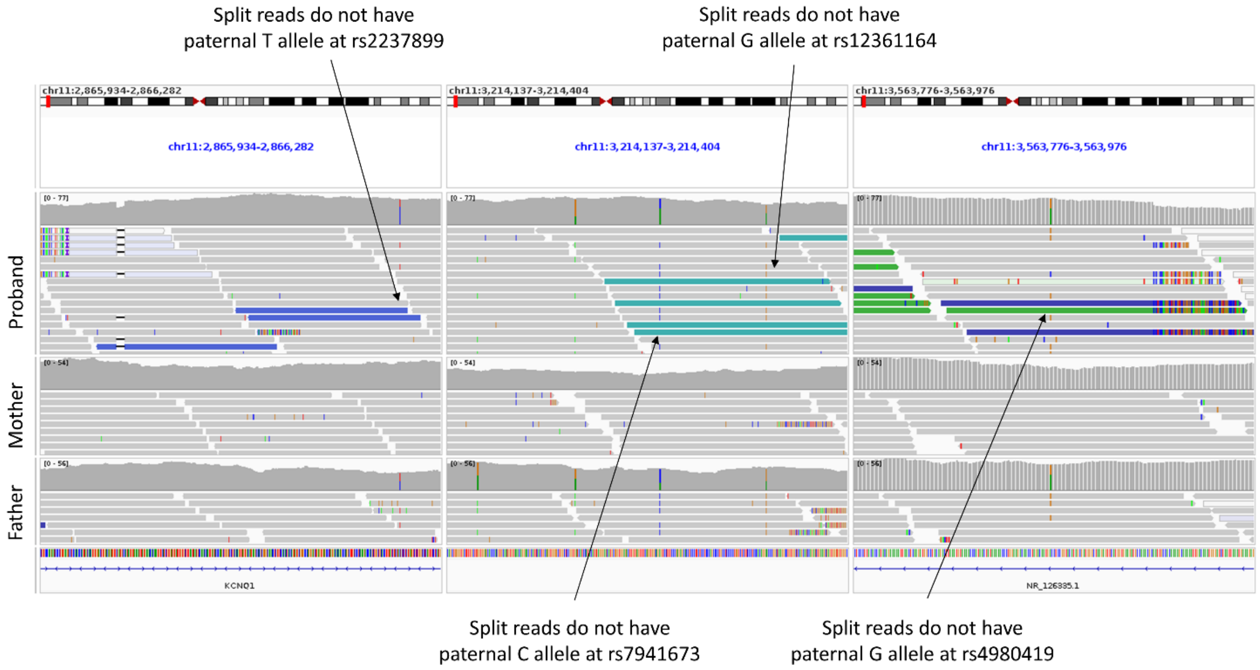

Figure S9: Informative SNPs close to the breakpoints allow phasing of the *de novo* SV found in Family 44 to the maternal chromosome. IGV screenshot shows split view corresponding to GRCh37 chr11:2865934-2866282 (rs2237899), chr11:3214137-3214404 (rs7941673, rs12361164) and chr11:3563776-3563976 (rs4980419). In each case the transmitted paternal non-reference allele is not present in read-pairs that span the SV breakpoints.

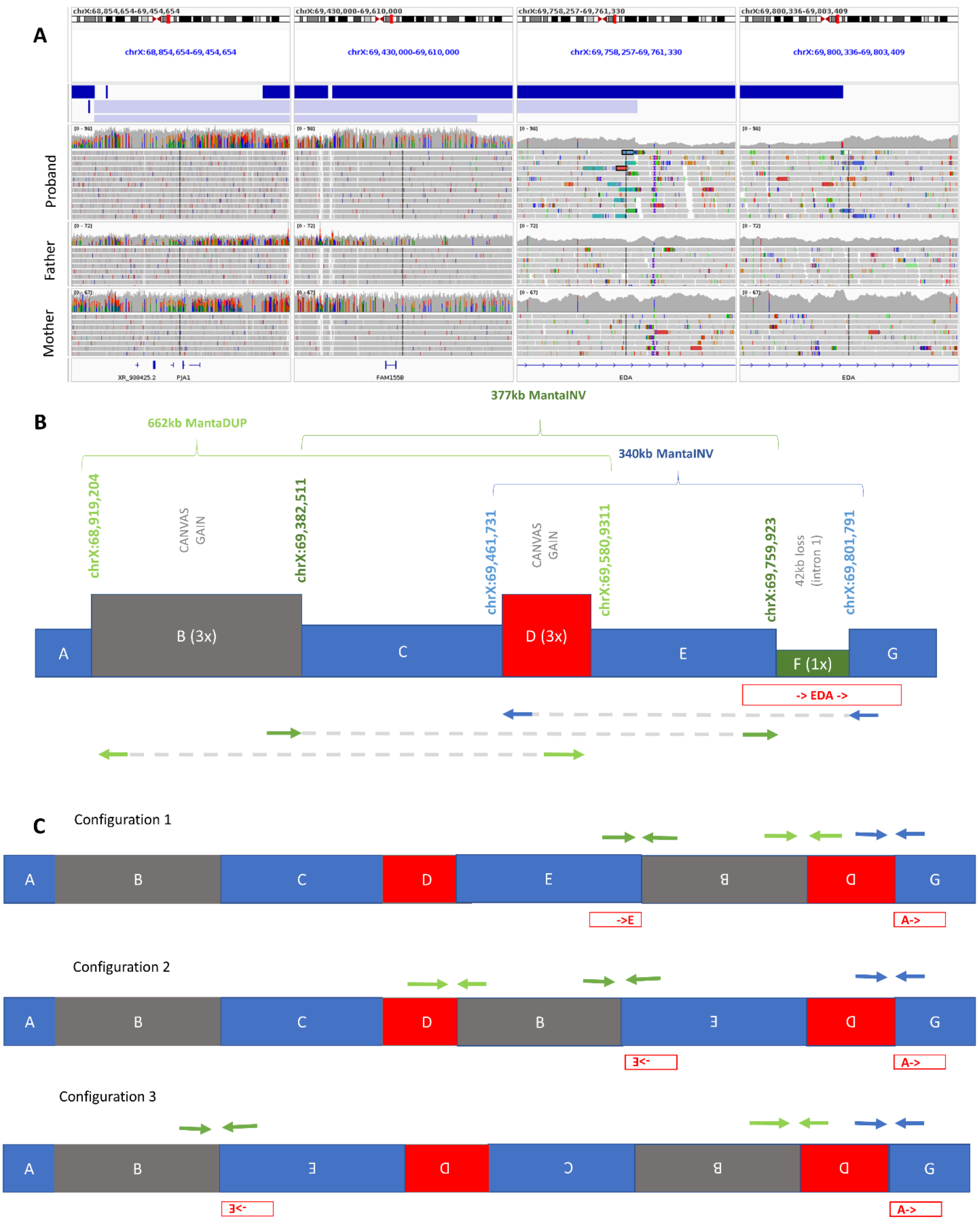

Figure S10: Read alignments and schematic diagram of a *de novo* SV involving *EDA* in Family 30. A) Read alignments shown in IGV highlighting (from LH to RHS); a 463kb gain, a 119kb gain and proximal/distal breakpoints of a 42kb deletion in intron 1 of *EDA*. The MantaINV calls (upper track) indicate that the duplicated segments are non-tandem and have been integrated at the position of the deletion. Genomic windows shown are chrX:68854654-69454654, chrX:69430000-69610000, chrX:69758257-69761330 and chrX:69800336-69803409. B) Schematic diagram (not to scale)

highlighting the duplicated segments of 463kb (grey) and 119kb (red), the relative orientation of the split reads and the resulting Manta SV calls. C) Three possible configurations can explain the short-read data as shown. For configurations #2 and #3, exon 1 of *EDA* has switched to the negative strand. However, even if configuration #1 is correct, insertion of 583kb and deletion of 42kb in intron 1 is likely to impact on correct splicing of the gene.

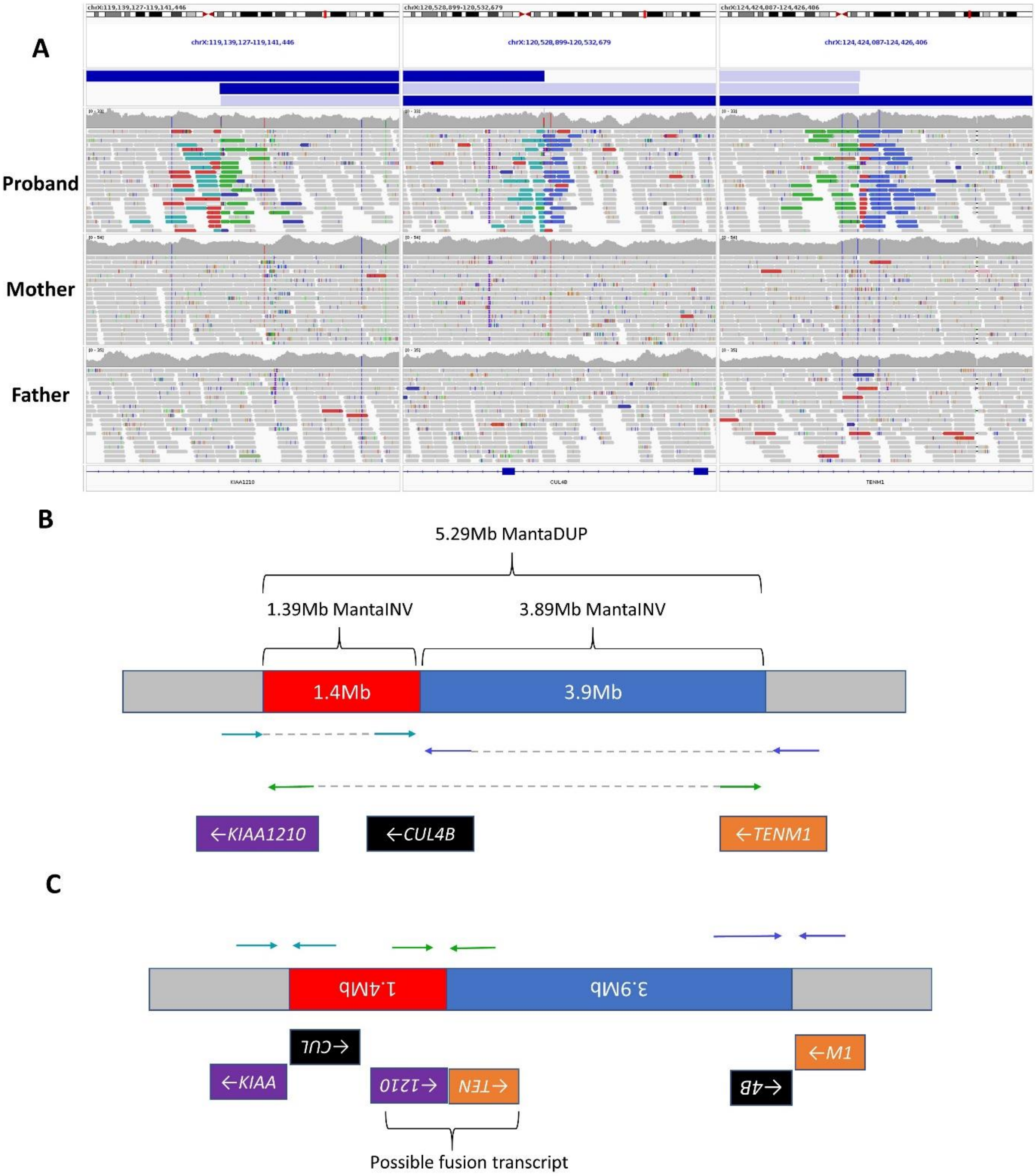

Figure S11: Complex *de novo* rearrangement in Family 39 that disrupts *CUL4B*. A) IGV screenshot showing alignments supporting two immediately adjacent inversions. Regions shown in the multi-region view are chrX:119,139,127-119,141,446 chrX:120,528,899-120,532,679 chrX:124,424,087-124,426,406 (GRCh38). B) Schematic diagram showing the relative split-read positions and Manta SVs compared to the reference genome and C) the configuration in the patient genome that can explain these pattern of split-reads. Although the breakpoints in intron 26 of 34 for *TENM1*

(NM_001163278.2) and intron 2 of 13 for *KIAA1210* (NM_020721.1) suggest the possibility of a fusion transcript involving *KIAA1210* and *TENM1*, the *TENM1* segment would be out of frame and therefore a gain of function mechanism seems unlikely.

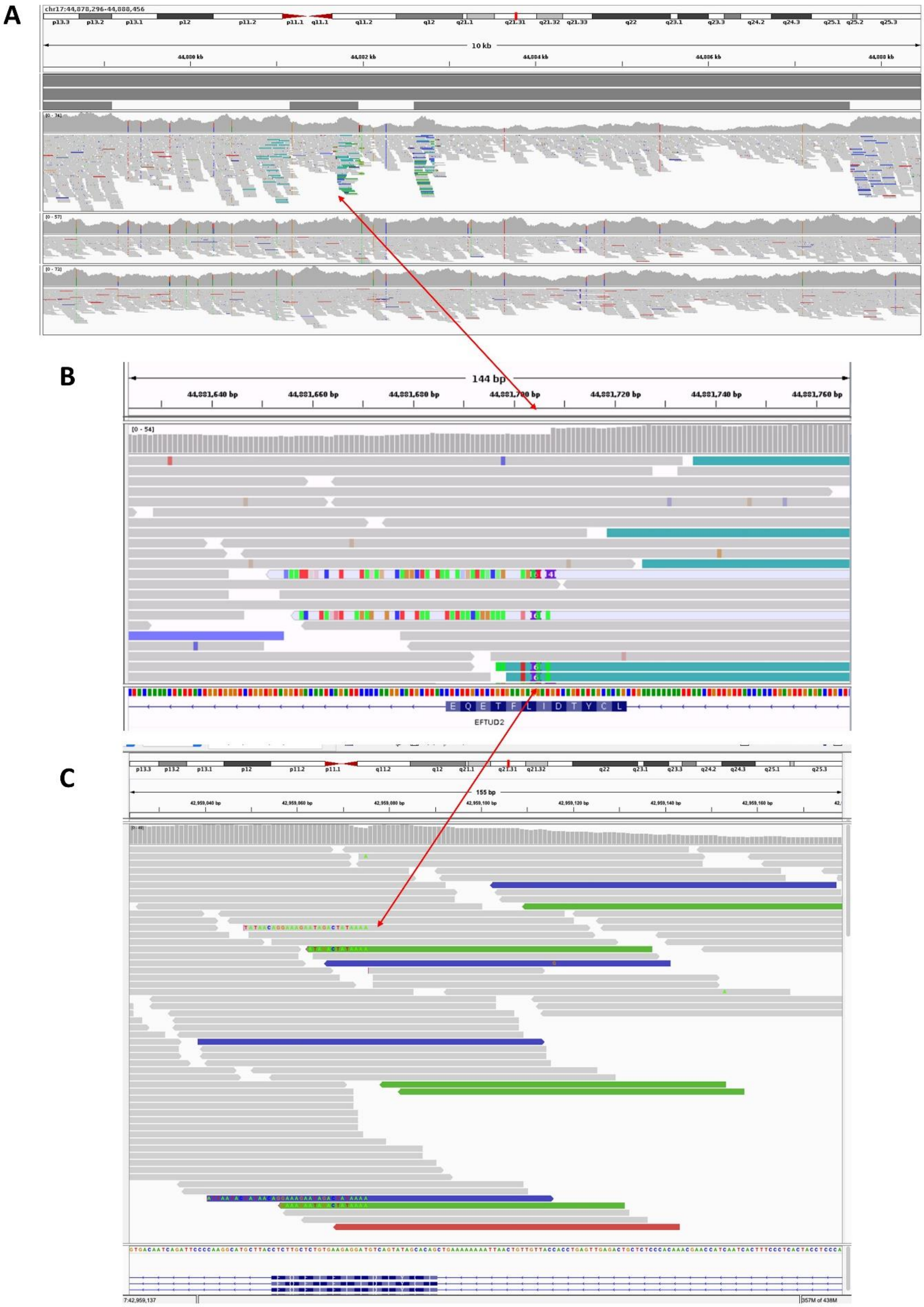

Figure S12: IGV screenshot showing read alignments supporting a complex SV involving *EFTUD2.* A) Zoomed out view showing drops in coverage and split read-pairs in proband (upper) but not in father

(middle) or mother (lower) suggesting *de novo* occurrence. The deletion contains 2 internal segments which are retained in an inverted orientation. Zoomed in view of B) genome sequence data and C) exome sequence data showing the same breakpoint in the middle of exon 7. Coordinates of the exome data are on GRCh37.

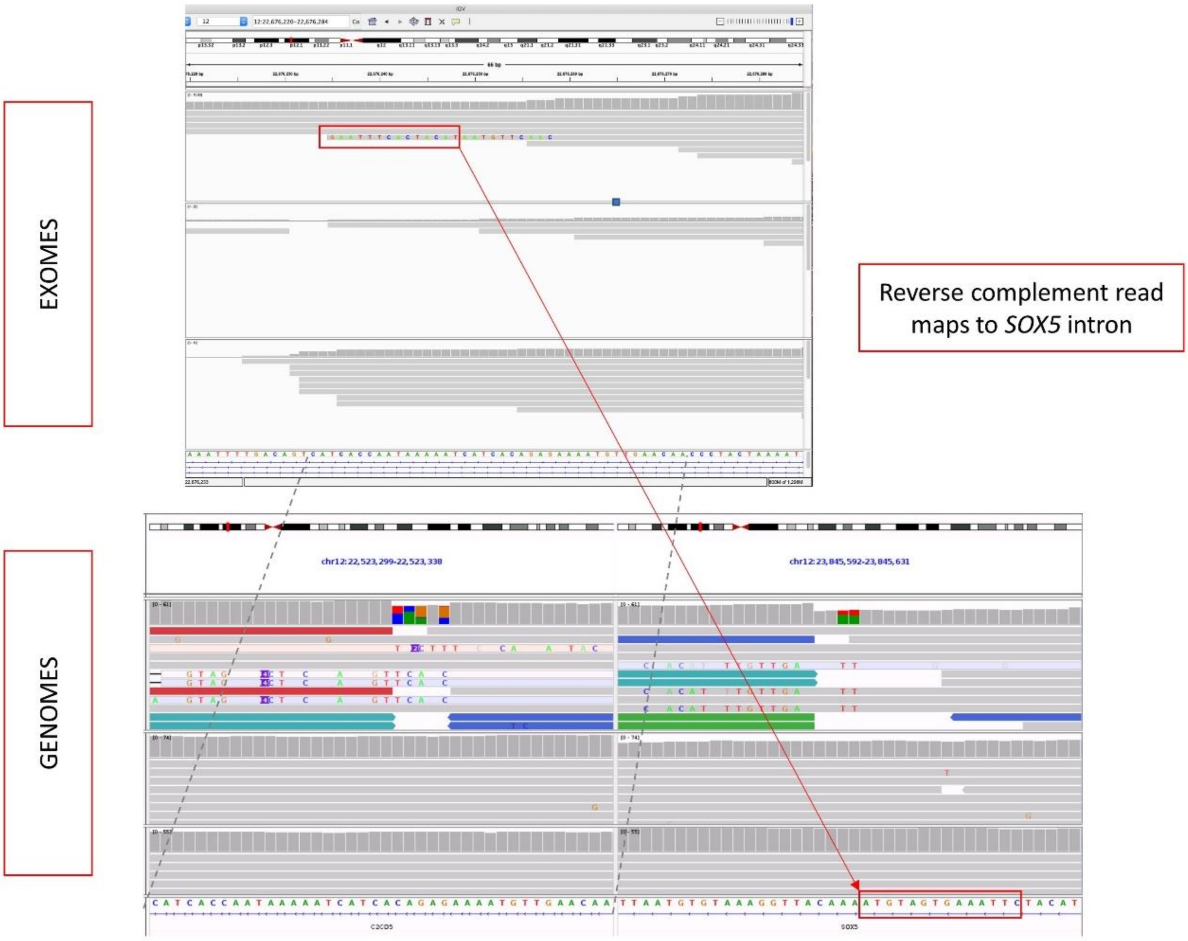

Figure S13: IGV screenshots showing read alignments supporting a *de novo* inversion disrupting *SOX5.* Top image shows trio exome data where a 1/6 reads from the proband (upper) has soft clipped sequence which maps 1.3Mb away to intron 3 of *SOX5,* but in an inverted orientation.

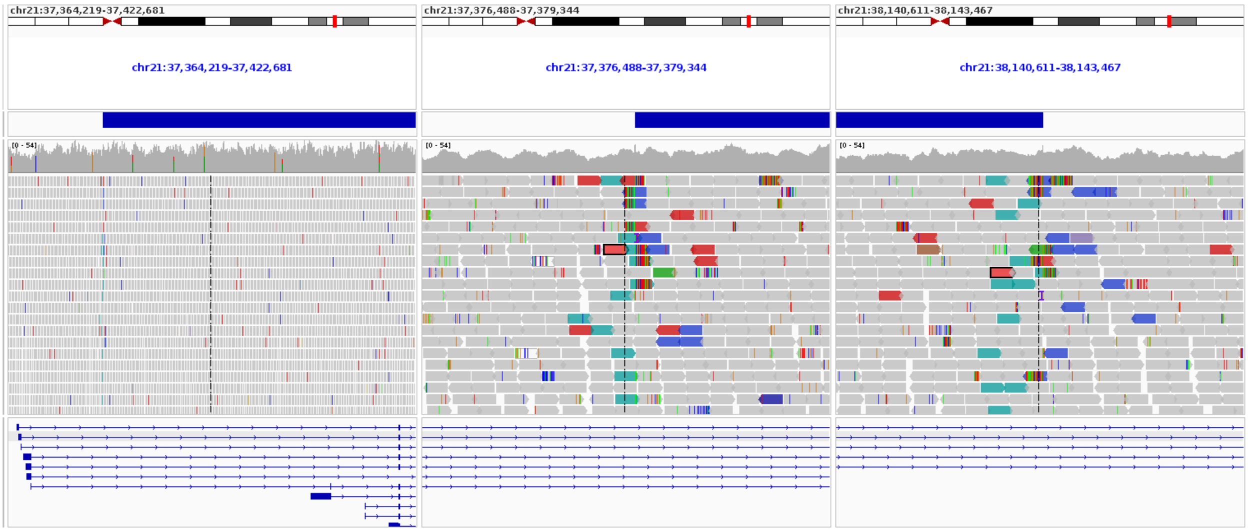

Figure S14: Read alignments and Manta calls supporting 764kb inversion that disrupts the 5'-UTR region of *DYRK1A*. The zoomed-out view (left panel) shows that, based on the canonical isoform (NM_001347721.2) and the majority of other RefSeq annotations, the proximal breakpoint lies in intron 1, whilst the start codon is in exon 2. Zoomed in views are shown of the proximal (centre panel) and distal breakpoints (right panel), the latter which lies in *DSCR8*. GRCh38 coordinates for the three IGV windows shown are chr21:37,364,219-37,422,681, chr21:37,376,488-37,379,344 and chr21:38,140, 611-38,143,467.

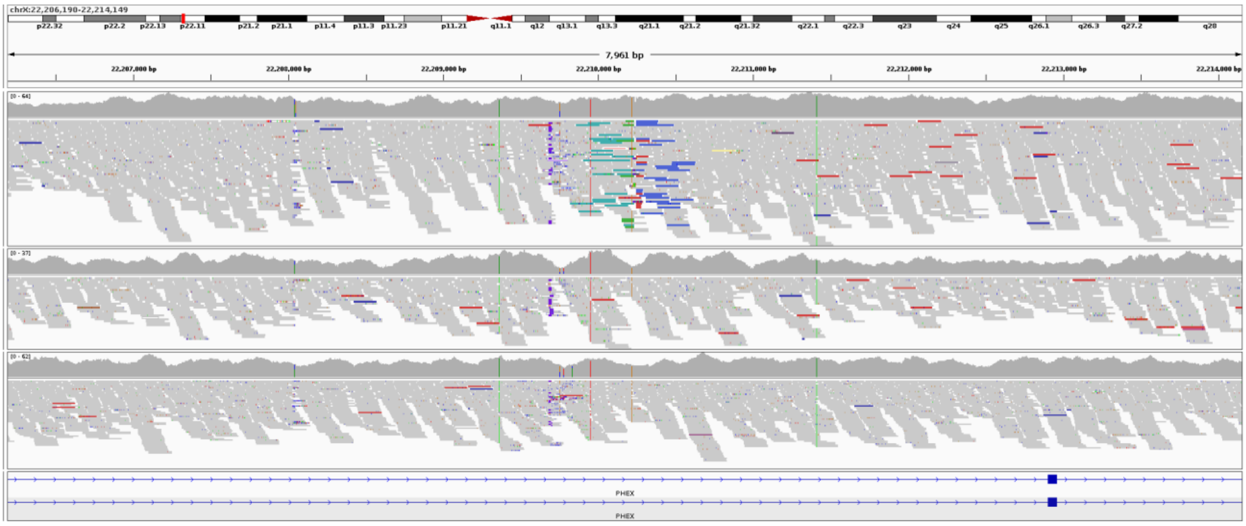

Figure S15: Read alignments supporting a 2.6Mb inversion involving *PHEX* (NM_000444.6). Only the proximal breakpoint in intron 15 is shown. This SV was identified in an individual with suspected hypophosphatemic rickets (Family 26). The absence of split read-pairs in the father (middle track) and mother (bottom) suggest that the inversion arose *de novo*.

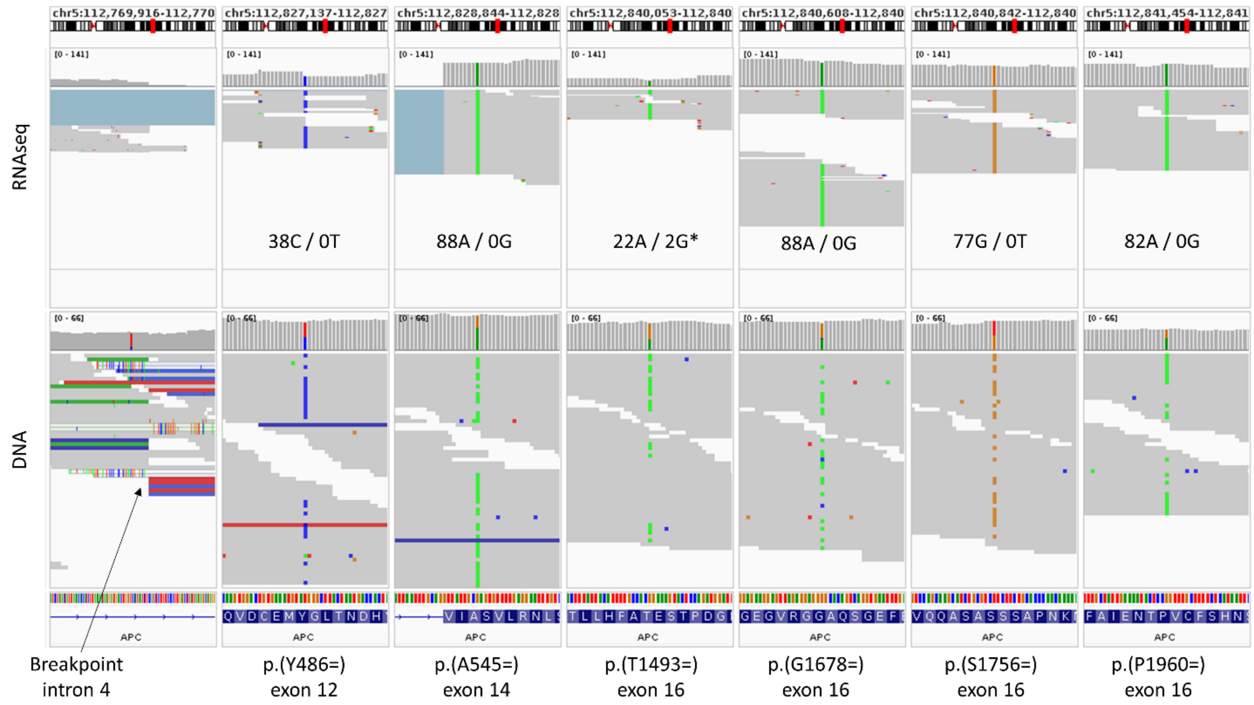

Figure S16: RNAseq data indicates that the complex *APC* translocation in Family 43 results in monoallelic expression. RNAseq data for the proband (F43-II-2; upper track) is compared to the genome sequencing data (lower) for the same individual. Monoallelic expression is apparent for a common 6 SNP haplotype (rs2229992-rs351771-rs41115-rs42427-rs866006-rs465899; C-A-A-A-G-A) spanning exons 12-16 (NM_000038.6). Only the non-reference alleles were expressed. PacBio data (available for F43-I-1) confirmed that the SV lies *in cis* with the reference alleles at these respective sites. *both Gs lie at the ends of reads.

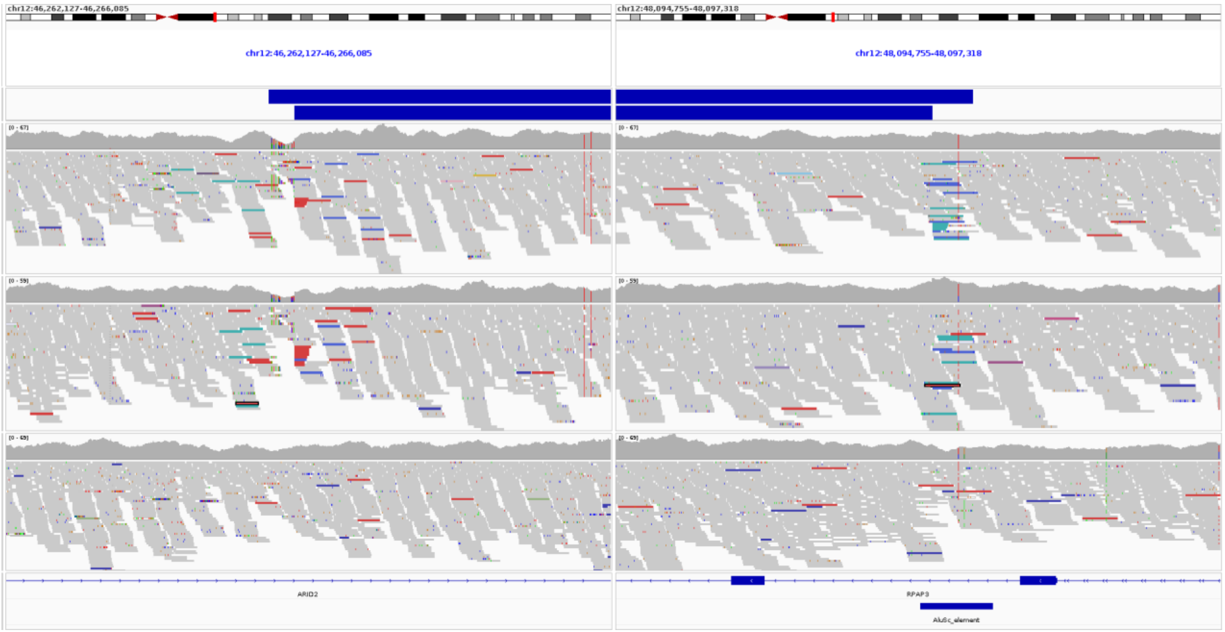

Figure S17: Read alignments and Manta calls suggesting possibility of a 1.8Mb inversion disrupting *ARID2* in Family 35 in proband (top) and inherited from the mother (middle). The bottom track is a control genome sequenced in the same batch as the mother. Although the +ve strand split read pairs (green) and the -ve strand split read pairs (blue) lie distinctly at each side of the breakpoint on the

proximal side, at the distal end they overlap and coincide with an intronic AluSc element. A more likely explanation of this data is therefore an intronic retrotransposon event into *ARID2* intron 16.

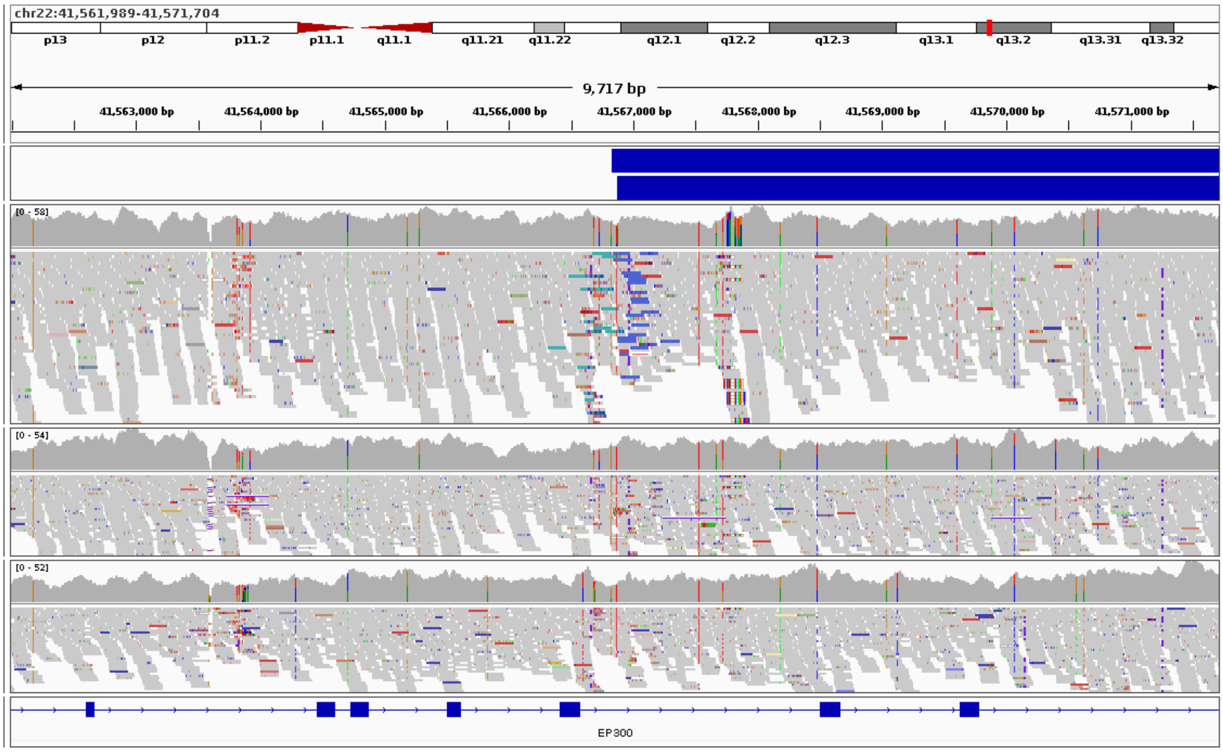

Figure S18: IGV screenshot showing read alignments supporting the 1.0Mb inversion on 22q13.2 in Family 18 that disrupts *EP300* and *TCF20*. In this view, only the proximal breakpoint that disrupts *EP300* in intron 27/30 (NM_001429.4) is shown. The horizontal blue bars in the top track show the reciprocal MantaINV calls. The parental data is shown in the two tracks immediately below that of the proband, confirming the SV to have arisen *de novo*.

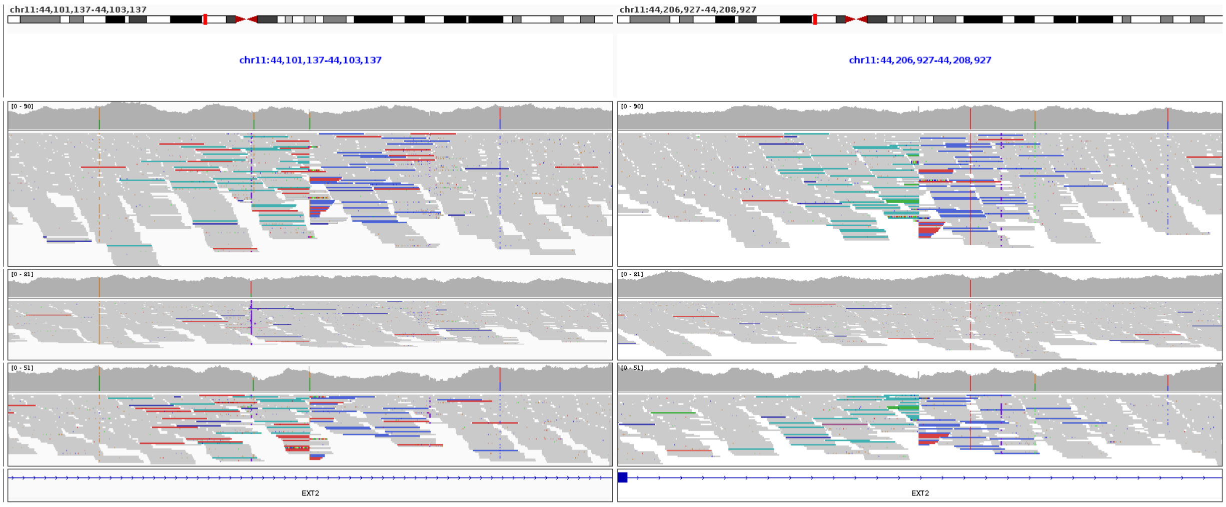

Figure S19: IGV screenshot showing read alignments supporting the 106kb inversion disrupting *EXT2* in Family 4. The inversion is seen in the proband (upper) and is inherited from the father (bottom) but not seen in the mother's data (middle). The reads are shown using the split-window option so that both the proximal and distal breakpoints can be viewed at the same time. The structural rearrangement inverts exons 2-10 of the 14 exon gene.

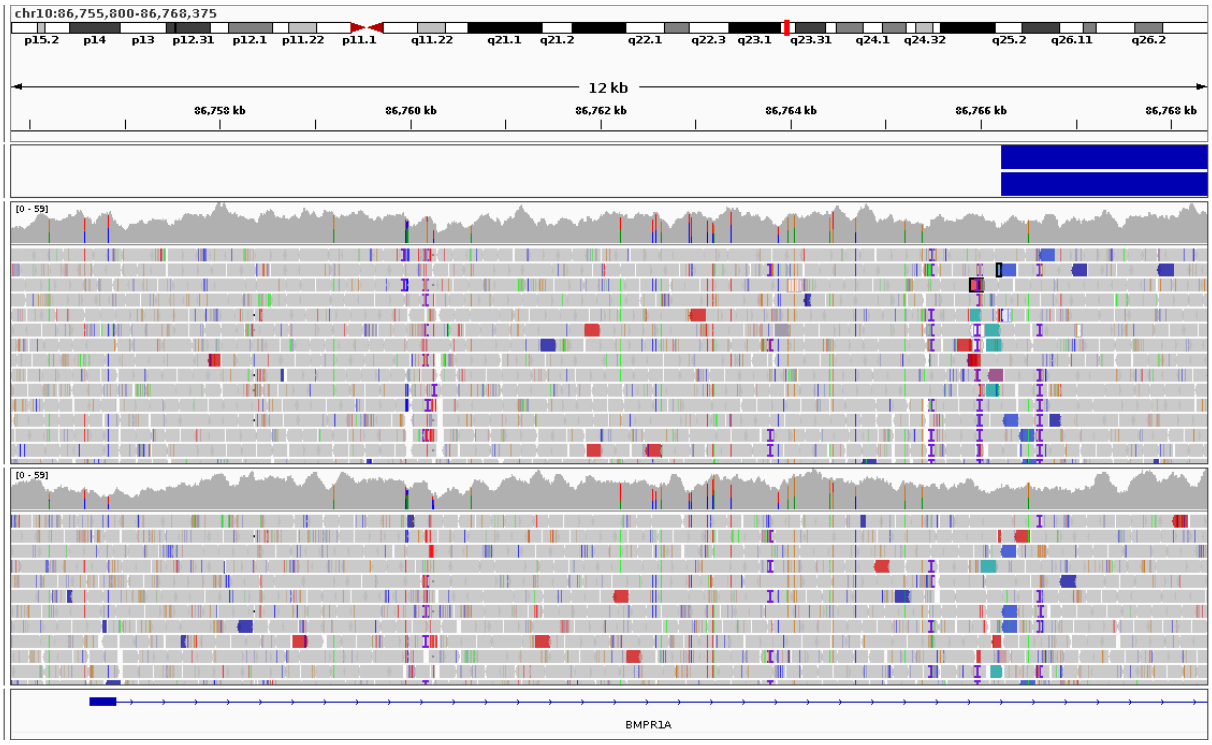

Figure S20: Read alignments and reciprocal MantaINV calls for a 1.4Mb inversion with a proximal breakpoint in intron 1 of *BMPR1A*. The inversion is seen in both the proband in Family 1 (upper) and in her affected mother (lower); both these individuals have a phenotype consistent with classical juvenile polyposis syndrome.

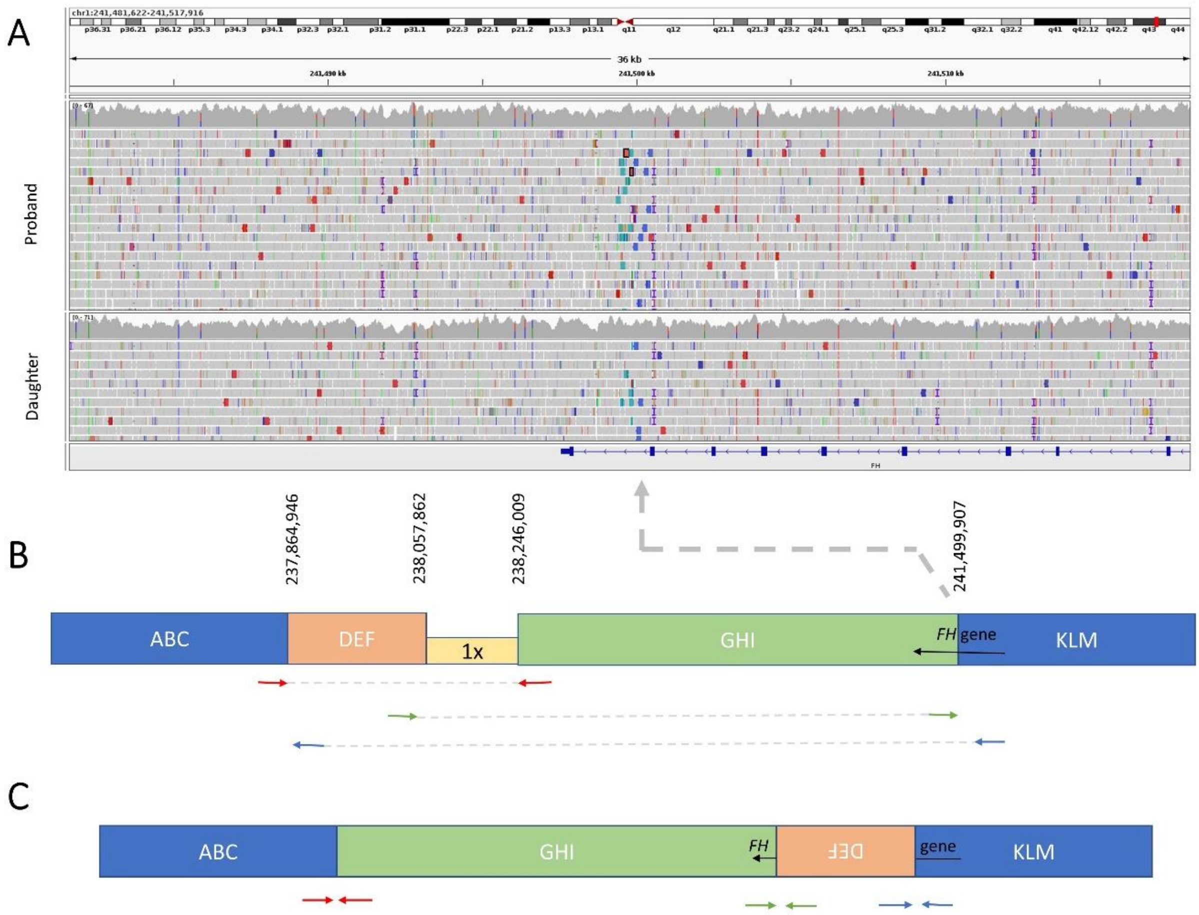

Figure S21: Read alignments and schematic diagrams explaining the structure of a complex rearrangement disrupting the fumarate hydratase gene. A) IGV screenshot showing split read pairs highlighted in green/blue that signify a breakpoint in the final intron of *FH*. B) Diagram summarising the positions of the split read-pairs. Plus to plus strand mappings are shown in red arrows and minus to minus strand mappings are shown as green arrows. Chromosomal segments are labelled A-M to help with orientation. Although mostly balanced, the rearrangement also involves a deleted segment (yellow) of 188kb in size. C) Schematic diagram showing the structure of patient genome that explains the split read-pairs in panel B. Segments are not shown to scale and genomic coordinates are based on GRCh38.

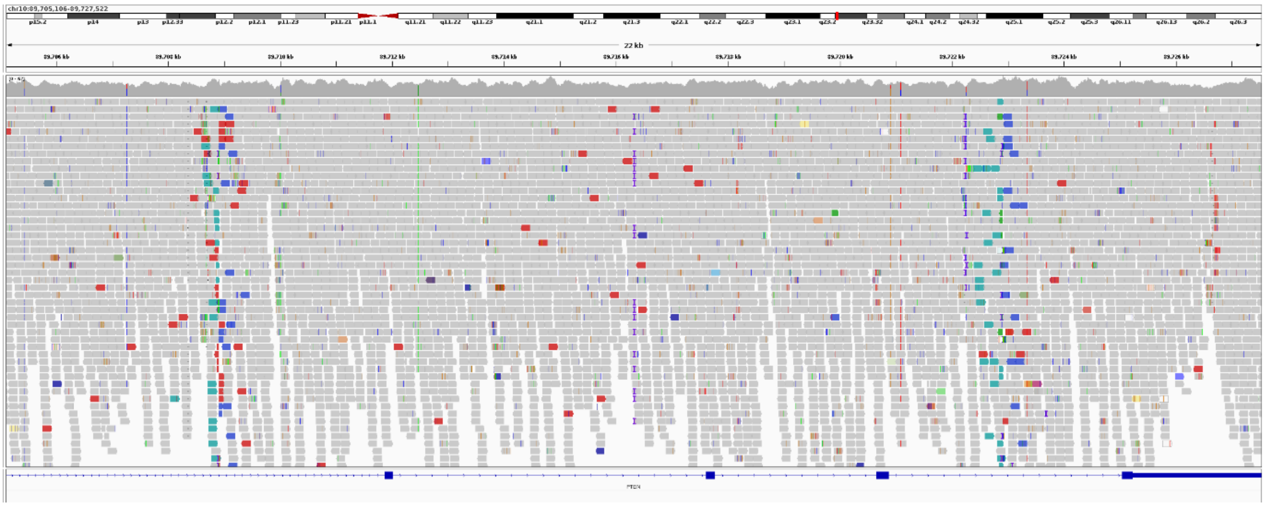

Figure S22: Read alignments supporting a 14kb inversion in Family 6 that disrupts *PTEN*. The rearrangement involves exons 6-8 and so is highly likely to disrupt gene function. Although the data shown here is on GRCh37, the coordinates were lifted over to GRCh38 for the purposes of SVRare and for Tables 1 and S2.

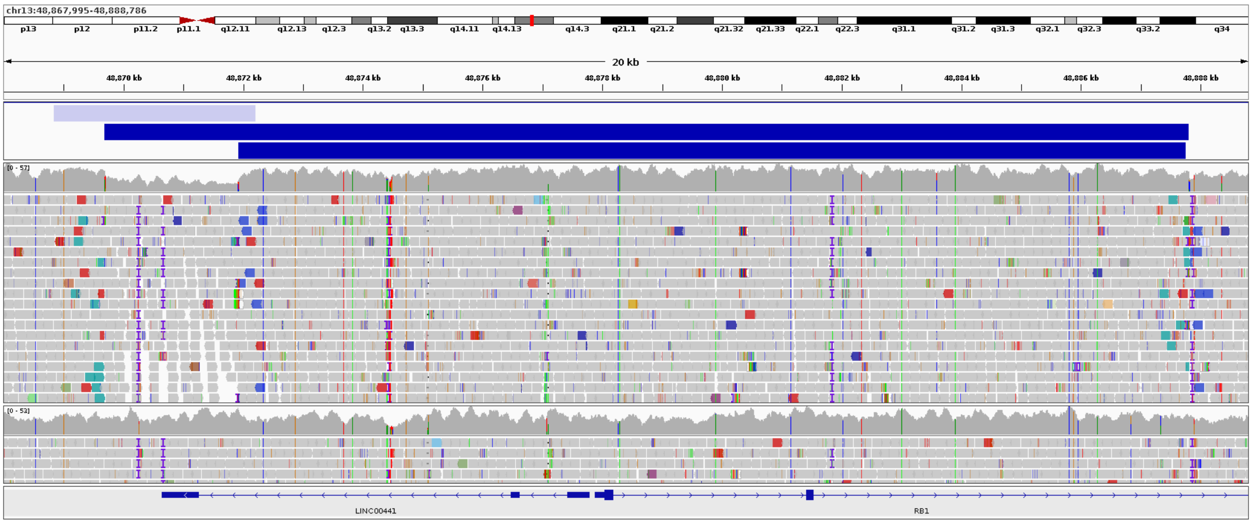

Figure S23: Read alignments supporting an 18.1kb inversion in Family 8 that disrupts *RB1*. The variant is private to this family amongst data from the 100kGP and as the distal breakpoint lies in intron 2, it is highly likely to disrupt gene function. The proximal end of the inversion is associated with a 2246bp loss involving the last exon of *LINC00441.* Identification of the loss only would have prioritised the wrong gene. The top track shows the Canvas call overestimates the deleted region (light blue), whilst the Manta accurately detected both reciprocal breakpoints of the inversion (dark blue). The middle track shows read alignments from the patient and the bottom track shows data from a control individual. The data is shown on GRCh37 build but we note coordinates have been lifted over to GRCh38 in Table S2.

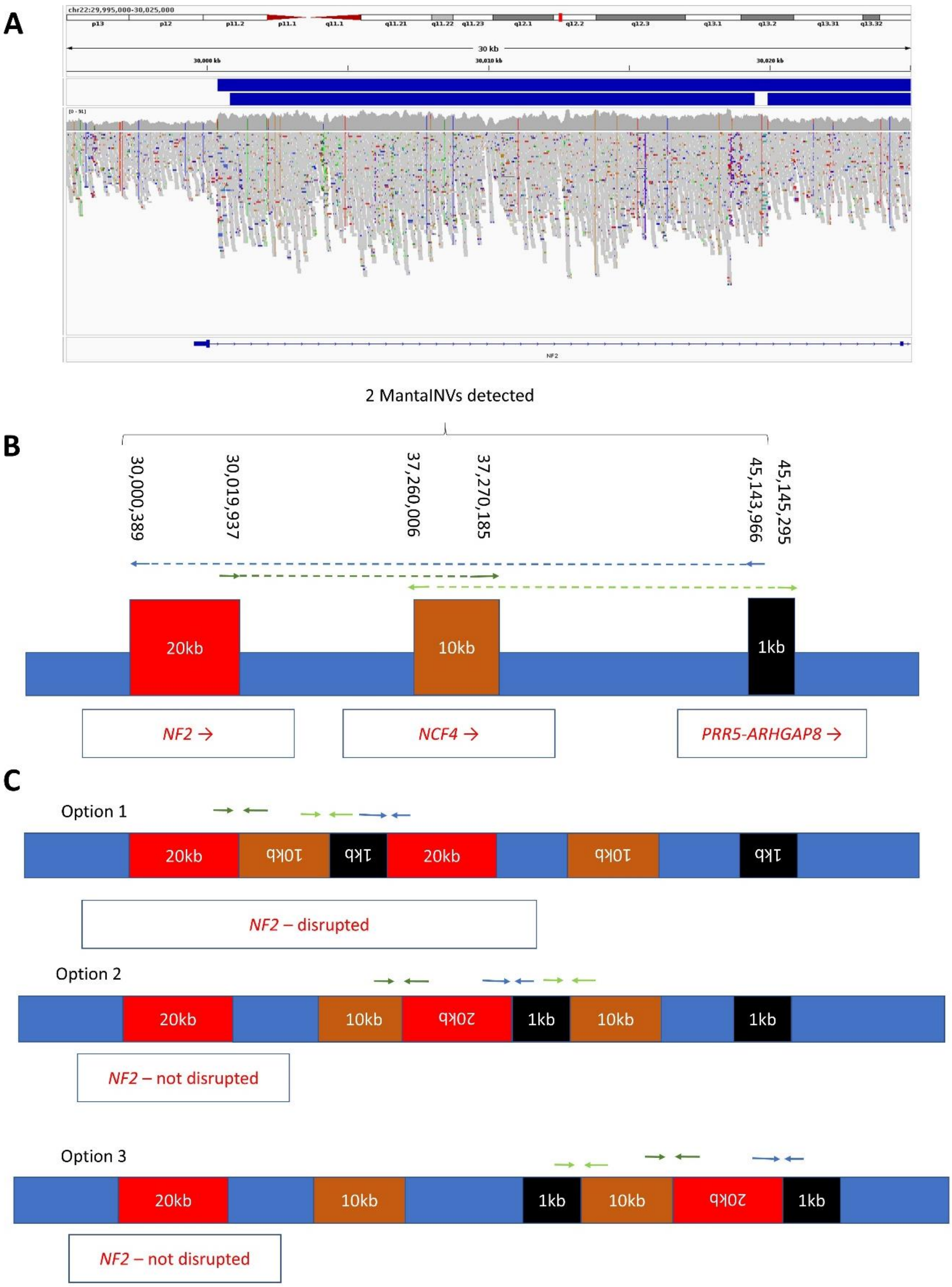

Figure S24: Complex SV involving *NF2* in Family 40. A) Read alignments shown in IGV are for the larger of the 3 duplicated segments which lies in intron 1 of *NF2*. B) Schematic diagram showing the 3 interlinked duplicated segments and the relative positions of the split read-pairs that result in the MantaINV call. *PRR5-ARHGAP8* refers to a readthrough transcript NM_181334.6. Genome coordinates are based on GRCh37 but the coordinates are converted to GRCh38 for Table S2. C) Schematic diagram showing 3 possible solutions to the short-read data. Only the first of these configurations is likely to

disrupt *NF2* and even then the duplication/insertion lies in intron 1. Clinical presentation and initial analysis of long-read PacBio sequencing data do not support options 2 and 3.

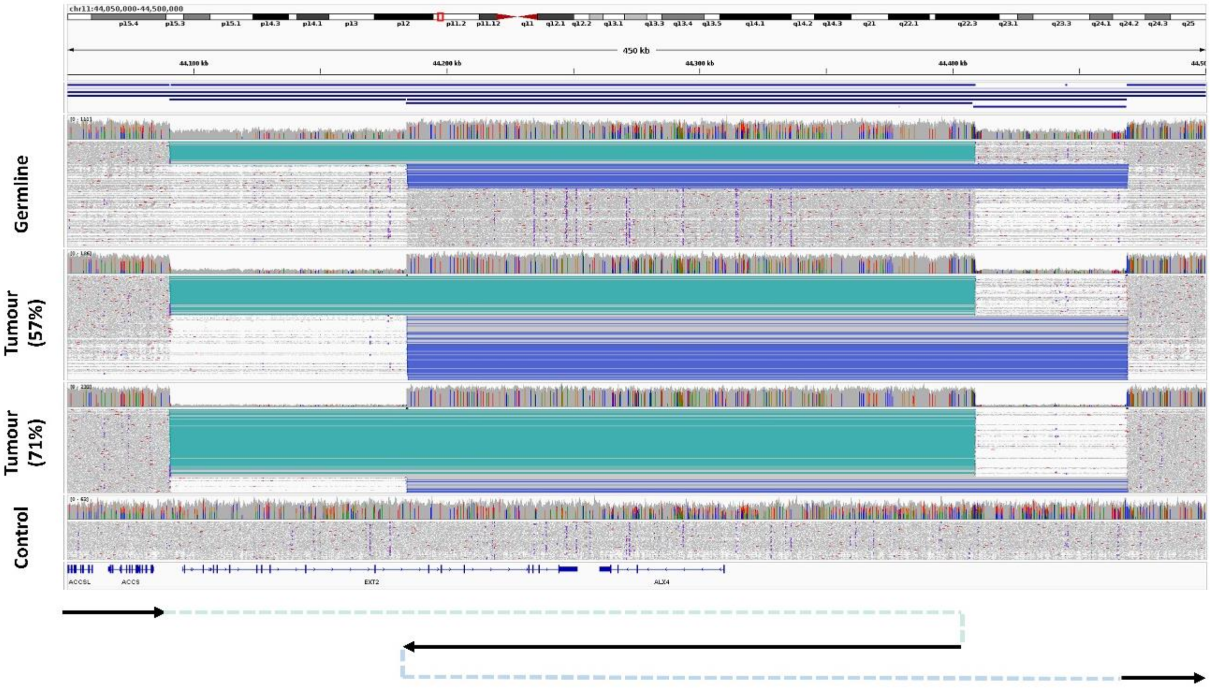

Figure S25: Example of a germline deletion-inversion from the cancer arm of the 100kGP that removes exons 1-8 of *EXT2* (NM_207122.2). The rearrangement was seen at a far higher allelic fraction in the tumour samples (of differing purity) due to a somatic chromosome 11 cnLOH event and so is predicted to result in a complete loss of *EXT2*. Multiple biopsies, all from the primary tumour of a participant with multiple exostoses and chondrosarcoma were sequenced alongside the germline. The +ve to +ve strand mapping read-pairs (teal) and the -ve to -ve mapping pairs (blue) indicates that the central segment has been inverted, as shown in the schematic diagram below. Reads are shown as pairs, squished and sorted by insert size. Genome coordinates are on GRCh38. The track at the top shows squished Manta/CANVAS calls. The control sample is a randomly selected sample sequenced in the same batch as the germline sample.

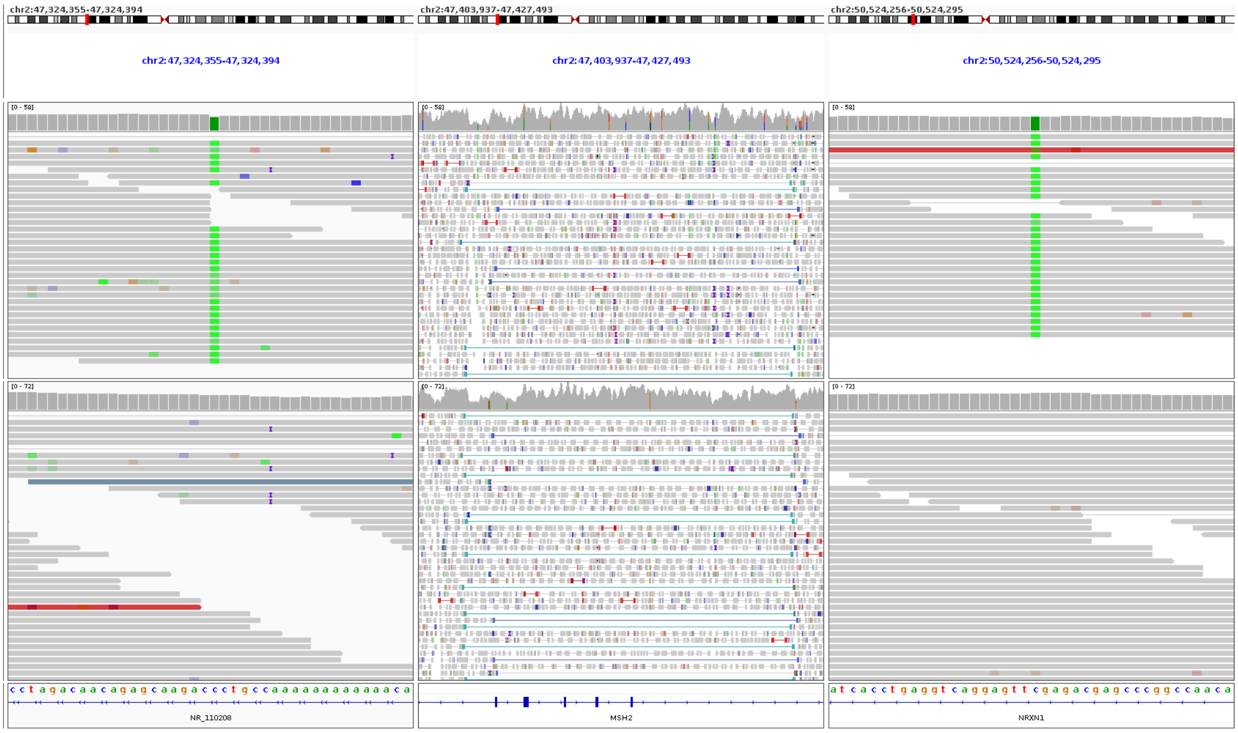

Figure S26: Conflicting homozygosity at common SNPs that flank the *MSH2* inversion haplotype and define the maximal shared region to be 3.2Mb (chr2:47,324,375-50,524,276, GRCh38). IGV image shows data for F16 (upper) and F17 (lower). The three windows show read alignments supporting rs115321698 (left), the inversion (middle) and rs13420048 (right).

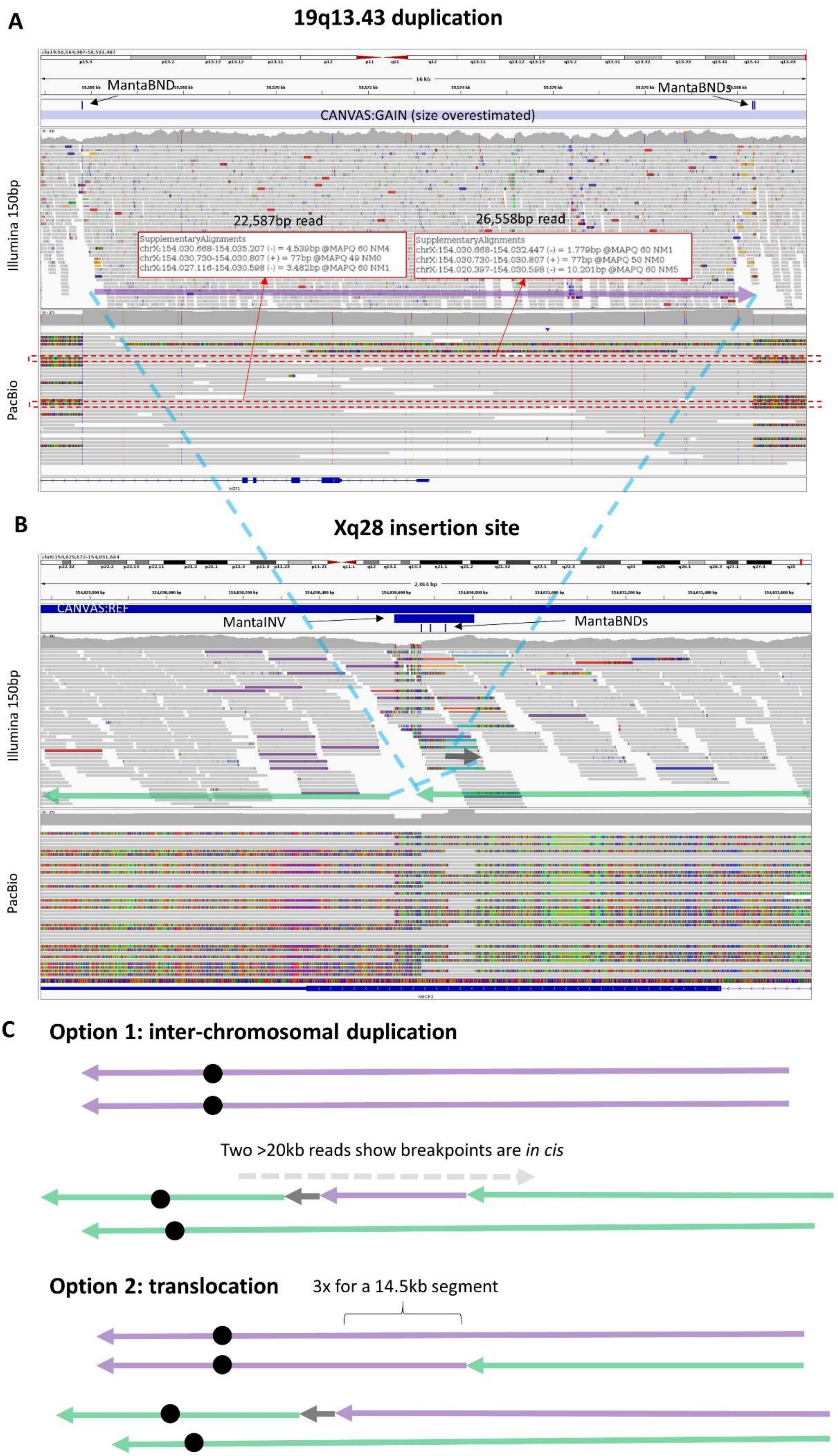
A
19q13.43 duplication
MantaBND
MantaBNDs
CANVAS:GAIN (size overestimated)
Illumina 150bp
22,587bp read
26,558bp read
SupplementaryAlignments
chrX:154,030,668-154,035,207 (-) = 4,539bp @MAPQ 60 NM4
chrX:154,030,730-154,030,807 (+) = 77bp @MAPQ 49 NM0
chrX:154,027,116-154,030,598 (-) = 3,482bp @MAPQ 60 NM1
SupplementaryAlignments
chrX:154,030,668-154,032,447 (-) = 1,779bp @MAPQ 60 NM1
chrX:154,030,730-154,030,807 (+) = 77bp @MAPQ 50 NM0
chrX:154,020,397-154,030,598 (-) = 10,201bp @MAPQ 60 NM5
PacBio
B
Xq28 insertion site
CANVAS:REF
MantaINV
MantaBNDs
Illumina 150bp
PacBio
MECP2
C
Option 1: inter-chromosomal duplication
Two >20kb reads show breakpoints are *in cis*
Option 2: translocation
3x for a 14.5kb segment

Figure S27: Representation of a complex inter chromosomal rearrangement disrupting the final exon of *MECP2*. A) IGV screenshot showing Manta and CANVAS calls (upper panel), Illumina 150bp read alignments (middle) and PacBio long reads (lower) for the proband in Family 47. Two PacBio reads of >20kb are highlighted that span both breakpoints and for these the information about the supplementary alignments are shown. B) IGV image similar to above for the Xq28 locus, showing *in silico* calls and read alignments supporting the complex SV. Dotted blue lines highlight the junctions between the chromosome segments. C) schematic diagram highlighting the two possible configurations that could explain the short read WGS data. Chr19 is shown in purple whilst chrX segments are in green/grey, consistent with the colour coding in panels A/B. Due to the two long reads shown in panel A the presence of two derivative chromosomes (and thus a translocation event) could be ruled out.

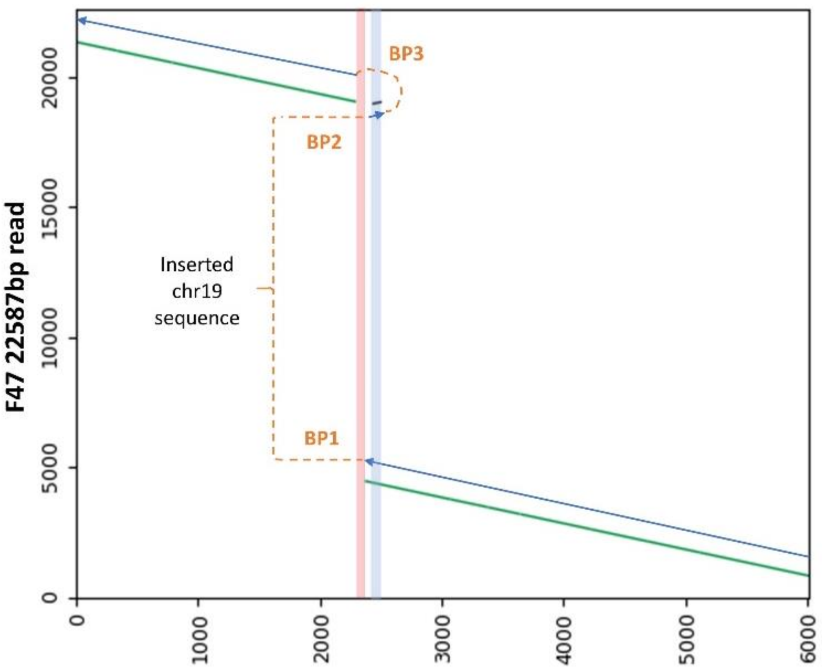

Figure S28: Dot-plot using the 22.6kb PacBio read indicated in Figure S27A showing the structure of a *de novo* inter-chromosomal duplication in Family 47 that involves the final exon of *MECP2*. To enable comparison to the *MECP2* rearrangement seen for F33, the X axis corresponds to the identical region shown in Figure 4 (chrX:154,028,301-154,034,315, GRCh38). Grey and green lines indicate sense/antisense matches to the reference, whilst the blue/orange lines help show how these segments are connected. The vertical red and blue shading highlights deleted and duplicated regions respectively.

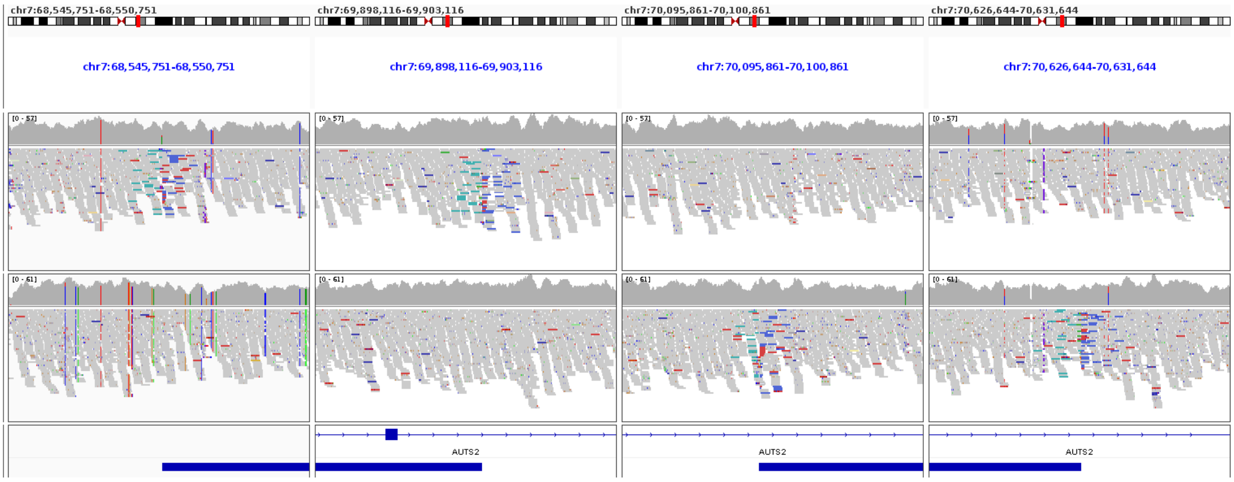

Figure S29: Proband-only read alignments and MantaINV calls for 2 *de novo* inversions involving *AUTS2* (NM_015570.4). For the 1.35Mb inversion in Family 37 (upper), the distal breakpoint lay near the start of intron 2. For the 531kb inversion in Family 38 (lower), the proximal and distal breakpoints lay towards the end of intron 2 and in the middle of intron 5, respectively. GRCh38 coordinates for the IGV windows shown are chr7:68545751-68550751, chr7:69898116-69903116, chr7:70095861-70100861 and chr7:70626644-70631644.

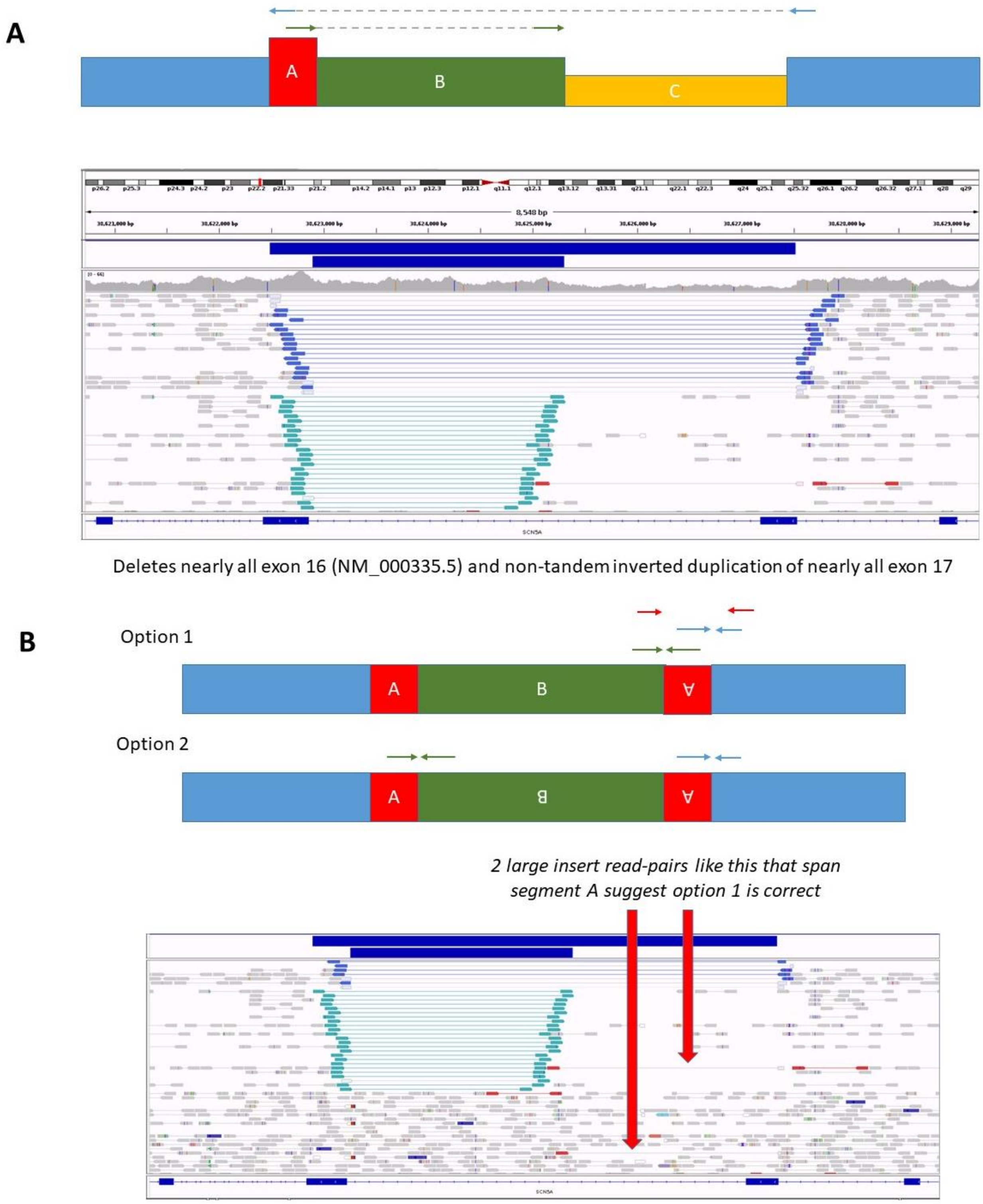

Figure S30: Schematic diagrams and read alignments supporting a *SCN5A* variant from the 100kGP pilot study. A) Schematic diagram highlights the different copy number states and split read-pairs. IGV screenshot shows read-pair alignments, which are sorted by size. B) Schematic diagram highlights two possible solutions to the short-read data. However, as there are two read pairs with large insert sizes that span the deleted region and also the 406bp segment A (red arrows), option 1 appears to be the correct orientation.

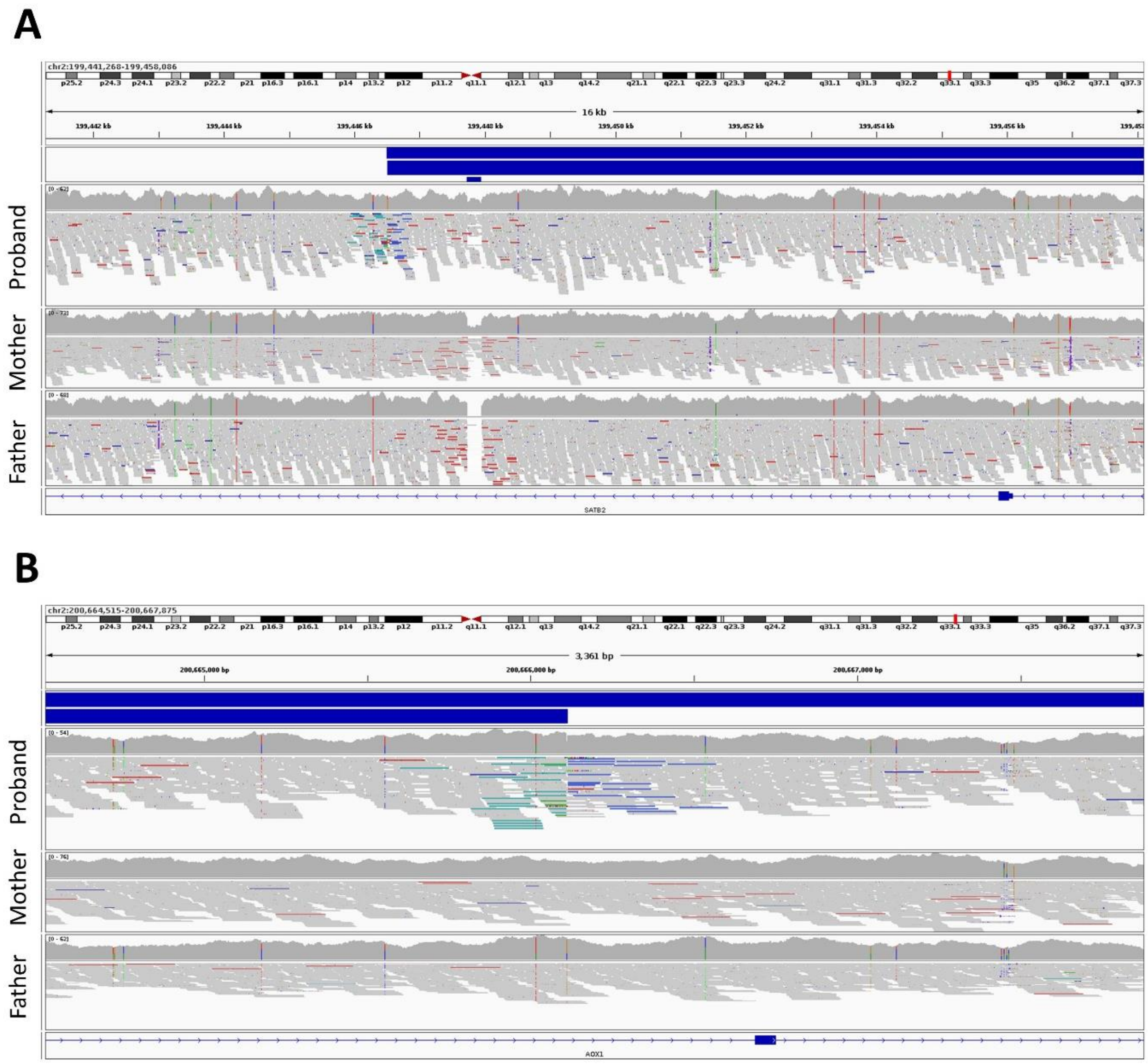

Figure S31: Read alignments supporting a *de novo* inversion involving *SATB2* (NM_015570.4) in Family 15. A) The proximal breakpoint of this 1.22Mb inversion lies in intron 2 of *SATB2,* whilst B) the distal breakpoint disrupts *AOX1*, a gene according to OMIM not known to be associated with disease.

Supplemental Tables

Table S1: Set of curated disease associated genes where haploinsufficiency is a known disease mechanism (HI Score = 3). List downloaded from ClinGen November 2022. Coordinates from initial download were from GRCh37 and so these were switched to GRCh38 using the UCSC LiftOver tool. *Table available as separate xlsx file.*

Table S2: Full details for 47 families with rare structural variants detected on account of a MantaINV call. *Table available as separate xlsx file.*

Table S3: Targeted validation strategies and PCR primers used for families where the SV has been confirmed with an orthogonal approach.

Family	Gene	Strategy (laboratory type)	Primers or other details
1	*BMPR1A*	PCR-Sanger (clinical)	BMPR1AChr10inv_P1F N13-TACCATGCCCAGCTAATTAAAAAAT<br>BMPR1AChr10inv_P1R N13-ACTGCCTAATCCGGGTGTTT<br>BMPR1AChr10inv_P2F N13-ATGGTACGGGTCGATTAATTTTTTA<br>BMPR1AChr10inv_P2R N13-TGACGGATTAGGCCCACAAA<br>BMPR1AChr10inv_D1F N13-TCAGAAAATGGAATAACTGCTTAAC<br>BMPR1AChr10inv_D1R N13-TTACCTTCATGGGATGCACA<br>BMPR1AChr10inv_D2F N13-AGTCTTTTACCTTATTGACGAATTG<br>BMPR1AChr10inv_D4R N13-AATGGAAGTACCCTACGTGT
2	*FH*	PCR-Sanger (clinical)	FH_BREAKPOINT_A_F N13-AACCCAAGGGCTGGATCAAA<br>FH_BREAKPOINT_A_R N13-ACCAAGGTTGACTTGGCCTG<br>FH_BREAKPOINT_B_F N13-CTGGGAAGAAAAAGAGGCTTA<br>FH_BREAKPOINT_B_R N13-GTTGTGGGAGAAACCTGGTG<br>FH_BREAKPOINT_C_F N13-TTAAGTGGAGGAGGCATTGG<br>FH_BREAKPOINT_C_R N13-AGTTTCATGTCATTGTGGTTAGAA<br>FH_BREAKPOINT_D_F N13-TGCAACATAATGCCTCAAAATC<br>FH_BREAKPOINT_D_R N13-CAATTCAGAAATGGAAAGTTACAA
5	*KMT2A*	PCR-Sanger (clinical)	Breakpoint 1 (NGS-4665)<br>Forward primer CCTCCTCTTGTACCTTGGCC<br>Reverse primer TGAGGGGAGGTGTTTGTTGG<br><br>Breakpoint 2 (NGS-4790)<br>Forward primer CACAGTCTCCATTCCTTGCCA<br>Reverse primer TCTCCCATCCCAAAGCAACC<br><br>Primers had the M13 tag for sequencing<br>M13F GTAAAACGACGGCCAGT<br>M13R CAGGAAACAGCTATGAC
6	*SOX5*	PCR-Sanger (clinical)	SOX5 Breakpoint 1F AGTGTTTCGATCTGGAGGGC<br>SOX5 Breakpoint 1R ACCAGGCTAGGCAACATGAC<br>SOX5 Breakpoint 2F CAGAGCCGGGAATAGTCACC<br>SOX5 Breakpoint 2R TCCATTTGCCTATCACCTGAAGG<br><br>SOX5 inversion breakpoint pair<br>Breakpoint 1F AGTGTTTCGATCTGGAGGGC<br>Breakpoint 2F CAGAGCCGGGAATAGTCACC
9	*FBN1*	PCR-Sanger (clinical)	As described (Family 3 in PMID:36411030)
13	*PAFAH1B1*	PCR-Sanger (clinical)	PAFAH1B1_BP1a_F GGGCATCAAAGGTGGTAGTG<br>PAFAH1B1_BP1a_R AACAGGGAATTACCAGCAAAAA<br>PAFAH1B1_Inv1a_F AGCGACAAGCCCTGCTAATA<br>PAFAH1B1_Inv1a_R CTGGTGGGATTACTGGCTTT<br>PAFAH1B1_Inv2a_F CTCAGTGGGGAGTGCTAGAG<br>PAFAH1B1_Inv2a_R GGCATCAAAGGTGGTAGTGC
14	*KMT2B*	Digital droplet PCR (clinical)	KMT2B_ddIn1_F_V1_and_KMT2B_ddIn1_R_V1<br>GGAAAGGGCCTCTGGAAGTG CGAAAAGGATCGGCCCAAGA<br><br>KMT2B_dd12_F_V1_and_KMT2B_dd12_R_V1<br>TCTGCTGTGACCCATTCCAC GTCCACAGACGTGGCAGAAT<br><br>KMT2B_dd13_F_V1_and_KMT2B_dd13_R_V1<br>CATACCACCCGGCCTGTC AGCAAGTGGGTGAACCTCAT

17	*MSH2*	PCR and Sanger (clinical)	Primers described elsewhere (PMID: 26498247)
21	*KMT2E*	PCR-Sanger (research & clinical)	Forward ACATTTACGCTTGGAAATTAAAA<br>Reverse GCTCTCTGATAACTCCTTTCTCTGA.
22	*GLI3*	PCR-Sanger (research)	As described (Family 2 in PMID:36411030)
23	*GLI3*	PCR-Sanger (clinical)	As described (Family 1 in PMID:36411030)
26	*PHEX*	PCR-Sanger (research)	PHEX-INV-1F TCTCTCACAAAGGTCACAGTCA<br>PHEX-INV-2F AAGATATTGAGTTGACCCTGTAG<br>PHEX-INV-1R CCCATGAGCCCAAACCTTCT<br>PHEX-INV-2R ACTTTTGCCGTTAGAAGCCC
30	*EDA*	PCR-Sanger (clinical)	EDA Breakpoint 1F GGGGAAATCTACCTAGGCACC<br>EDA Breakpoint 1R AGAGTGGGCTCAAGCATGAC<br>EDA Breakpoint 2F AGAGGTTGGAGAGGGAGTGG<br>EDA Breakpoint 2R CTCAGTCCTCTTCTGCTGGC<br><br>EDA inversion 1 breakpoint pair<br>Breakpoint 1F GGGGAAATCTACCTAGGCACC<br>Breakpoint 2F AGAGGTTGGAGAGGGAGTGG
33	*MECP2*	PCR-Sanger (research)<br>Single breakpoint	MECP2_Aii TGCAAATAATTCTAAGCTGTCCC<br>MECP2_DF GCCACCCACAAGTCTCCTA
38	*AUTS2*	Nanopore WGS (service/research)	Methods and analysis pipeline to be described elsewhere
39	*CUL4B*	PCR-Sanger (clinical)	Inv1: chrX: 118274086 – 119664466 (Build37)- KIAA1210 (R) + CUL4B (R)<br><br>KIAA1210-int2R GGGGCACATGGAGTCCTTTC<br>CUL4B-int20R TGCTGACAGAGAAAAATCCTACAAAC<br><br>Inv2: chrX:119664465 – 123558976 (Build37)- - CUL4B (F) + TENM1 (F)<br><br>CUL4B-int20F TGCTGCAAAAAGGCCAAACTG<br>TENM1-int23F CTCACCCCAGTTGGAATGGC
40	*NF2*	PCR-Sanger validated (clinical) and PacBio HiFi data	Described elsewhere (PMID: 38302265)
43	*APC*	Karyotyping (clinical), PacBio data (service, via Genomics England), PCR-nanopore of clinically relevant breakpoints (research)	Karyotyping confirms translocation and used for cascade testing, PacBio data confirms conformation, PCR-nanopore of selected breakpoints:<br><br>Breakpoint 1<br>APC-EF CTCTCCAGTTTCATATATGCCCA<br>APC-CR CAGGAGCATGGTGTGAGC<br>Breakpoint 2<br>APC-XR AGAGACTAGTGGTACTACAGGGA<br>APC-FR CTGAAATTCCCTCTCTCTGCT<br><br>Notes: The first targeted breakpoint contained a 92bp product (chr5:112,769,884-112,769,975) from *APC*, followed by sequence chr5:111,639,990-111,640,288 from *STARD4-AS1*. The second product contained 173bp from chr5:116,946,043-116,946,215, followed by 137bp of the proceeding sequence for the first junction chr5:112,769,977-112,770,113 within *APC*.

Table S4: Rare Variants defining inversion haplotype. Ultra rare variants (<0.1% AF in 100kGP) across the *MSH2* locus that are shared by the probands in Families 16 and 17. Although a 6Mb region was interrogated (chr2:45,450,067-51,450,067), all shared rare variants lay within the same 3.2Mb region identified by analysis of common variants (Figure 3C). Genomic positions are based on GRCh38. *MANE isoform unless otherwise stated. AggV2, aggregate vcf file with AN=156,390 unless otherwise stated. †ENST00000644092.1, ‡AN=156388. §Allelic read depths (ref/alt) for the individual reported by Brennan *et al*[11] from genome sequencing data (150-bp paired-end sequencing on a NovaSeq6000) were consistent with heterozygosity at all 13 positions.

Chr2 position	Ref/Alt	AF in AggV2	gnomAD AF (v4.0.0)	rsID	Gene (region)*	HGVSc	Allelic depth for Australian individual§
47,459,403	A/G	0.0352%	0.0197%	rs915614489	*MSH2* (intron 8 of 15)	c.1387-3628A>G	20/28
47,629,158	G/A	0.0454%	0.0151%	rs755620092	*MSH2* (intron 17 of 19†)	c.*1243-3644G>A	9/13
47,892,894	A/G	0.0121%	0.0026%	rs776369167	*FBXO11* (intron 1 of 22)	c.232+12595T>C	25/23
48,202,772	G/A	0.0109%	0.0013%	rs767041723	intergenic	NA	19/30
48,383,404	C/T	0.0403%	0.0118%	rs771247708	intergenic	NA	15/22
48,595,107	T/C	0.0090%	0.0013%	rs1374545554	*STON1* (intron 3 of 3)	c.2134-121T>C	14/15
48,998,287	G/A	0.0019%	0.0020%	rs1351434493	*FSHR* (intron 4 of 9)	c.375-7650C>T	18/19
49,099,629	A/T	0.0083%‡	Absent	rs1670951031	*FSHR* (intron 1 of 9)	c.153-31339T>A	22/25
49,234,481	C/T	0.0109%	0.0013%	rs902839622	intergenic	NA	21/23
49,437,430	C/A	0.0032%	Absent	rs1669735567	intergenic	NA	23/30
50,014,470	T/C	0.0058%	Absent	NA	*NRXN1* (intron 21 of 22)	c.4128+38801A>G	24/31
50,037,384	C/T	0.0959%	0.0507%	rs761040510	*NRXN1* (intron 21 of 22)	c.4128+15887G>A	22/25
50,095,187	A/G	0.0058%	Absent	NA	*NRXN1* (intron 18 of 22)	c.3547-3693T>C	20/30

References

1. Rhees, J., Arnold, M., and Boland, C.R. (2014). Inversion of exons 1-7 of the MSH2 gene is a frequent cause of unexplained Lynch syndrome in one local population. Fam Cancer *13*, 219-225. 10.1007/s10689-013-9688-x.
2. Moore, A.R., Yu, J., Pei, Y., Cheng, E.W.Y., Taylor Tavares, A.L., Walker, W.T., Thomas, N.S., Kamath, A., Ibitoye, R., Josifova, D., et al. (2023). Use of genome sequencing to hunt for cryptic second-hit variants: analysis of 31 cases recruited to the 100 000 Genomes Project. J Med Genet. 10.1136/jmg-2023-109362.
3. Loftus, S.K., Lundh, L., Watkins-Chow, D.E., Baxter, L.L., Pairo-Castineira, E., Nisc Comparative Sequencing, P., Jackson, I.J., Oetting, W.S., Pavan, W.J., and Adams, D.R. (2021). A custom capture sequence approach for oculocutaneous albinism identifies structural variant alleles at the OCA2 locus. Hum Mutat *42*, 1239-1253. 10.1002/humu.24257.
4. Shirts, B.H., Salipante, S.J., Casadei, S., Ryan, S., Martin, J., Jacobson, A., Vlaskin, T., Koehler, K., Livingston, R.J., King, M.C., et al. (2014). Deep sequencing with intronic capture enables identification of an APC exon 10 inversion in a patient with polyposis. Genet Med *16*, 783-786. 10.1038/gim.2014.30.
5. Xu, L., Wang, X., Lu, X., Liang, F., Liu, Z., Zhang, H., Li, X., Tian, S., Wang, L., and Wang, Z. (2023). Long-read sequencing identifies novel structural variations in colorectal cancer. PLoS Genet *19*, e1010514. 10.1371/journal.pgen.1010514.
6. Su, L.K., Steinbach, G., Sawyer, J.C., Hindi, M., Ward, P.A., and Lynch, P.M. (2000). Genomic rearrangements of the APC tumor-suppressor gene in familial adenomatous polyposis. Hum Genet *106*, 101-107. 10.1007/s004399900195.
7. Nakamura, W., Hirata, M., Oda, S., Chiba, K., Okada, A., Mateos, R.N., Sugawa, M., Iida, N., Ushiama, M., Tanabe, N., et al. (2024). Assessing the efficacy of target adaptive sampling long-

read sequencing through hereditary cancer patient genomes. NPJ Genom Med *9*, 11. 10.1038/s41525-024-00394-z.
8. Bozsik, A., Butz, H., Grolmusz, V.K., Polgar, C., Patocs, A., and Papp, J. (2023). Genome sequencing-based discovery of a novel deep intronic APC pathogenic variant causing exonization. Eur J Hum Genet *31*, 841-845. 10.1038/s41431-023-01322-y.
9. Weisschuh, N., Mazzola, P., Zuleger, T., Schaeferhoff, K., Kuhlewein, L., Kortum, F., Witt, D., Liebmann, A., Falb, R., Pohl, L., et al. (2023). Diagnostic genome sequencing improves diagnostic yield: a prospective single-centre study in 1000 patients with inherited eye diseases. J Med Genet. 10.1136/jmg-2023-109470.
10. Horton, A.E., Lunke, S., Sadedin, S., Fennell, A.P., and Stark, Z. (2023). Elusive variants in autosomal recessive disease: how can we improve timely diagnosis? Eur J Hum Genet *31*, 371-374. 10.1038/s41431-023-01293-0.
11. Brennan, B., Hemmings, C.T., Clark, I., Yip, D., Fadia, M., and Taupin, D.R. (2017). Universal molecular screening does not effectively detect Lynch syndrome in clinical practice. Therap Adv Gastroenterol *10*, 361-371. 10.1177/1756283X17690990.
