# Supplementary material for: The impact of inversions across 33,924 families with rare disease from a national genome sequencing project
Source: Am J Hum Genet. 2024 May 21;111(6):1140–64. doi: 10.1016/j.ajhg.2024.04.018 (PMC11179413; doi:10.1016/j.ajhg.2024.04.018)
Supplement: Document S2. Article plus supplemental information [file mmc4.pdf]

The impact of inversions across 33,924 families with rare disease from a national genome sequencing project

Alistair T. Pagnamenta,[1,*] Jing Yu,[1,2] Susan Walker,[3] Alexandra J. Noble,[4] Jenny Lord,[5,6] Prasun Dutta,[1] Mona Hashim,[1] Carme Camps,[1] Hannah Green,[7] Smrithi Devaiah,[8] Lina Nashef,[9] Jason Parr,[10] Carl Fratter,[7] Rana Ibnouf Hussein,[10] Sarah J. Lindsay,[11] Fiona Lalloo,[10] Benito Banos-Pinero,[7] David Evans,[12] Lucy Mallin,[12] Adrian Waite,[13] Julie Evans,[13] Andrew Newman,[14] Zoe Allen,[15] Cristina Perez-Becerril,[10] Gavin Ryan,[16] Rachel Hart,[17] John Taylor,[7] Tina Bedenham,[7] Emma Clement,[18] Ed Blair,[8] Eleanor Hay,[18] Francesca Forzano,[19] Jenny Higgs,[17] Natalie Canham,[17] Anirban Majumdar,[20] Meriel McEntagart,[21] Nayana Lahiri,[21] Helen Stewart,[8] Sarah Smithson,[22] Eduardo Calpena,[23,24] Adam Jackson,[10,25] Siddharth Banka,[10,25] Hannah Titheradge,[26] Ruth McGowan,[27] Julia Rankin,[28] Charles Shaw-Smith,[28] D. Gareth Evans,[10,25] George J. Burghel,[10] Miriam J. Smith,[10] Emily Anderson,[17] Rajesh Madhu,[29] Helen Firth,[11] Sian Ellard,[12] Paul Brennan,[30] Claire Anderson,[31] Doug Taupin,[32] Mark T. Rogers,[14] Jackie A. Cook,[33] Miranda Durkie,[34] James E. East,[4] Darren Fowler,[35] Louise Wilson,[18] Rebecca Igbokwe,[26] Alice Gardham,[18] Ian Tomlinson,[36] Diana Baralle,[5] Holm H. Uhlig,[1,4] and Jenny C. Taylor[1,*]

Summary

Detection of structural variants (SVs) is currently biased toward those that alter copy number. The relative contribution of inversions toward genetic disease is unclear. In this study, we analyzed genome sequencing data for 33,924 families with rare disease from the 100,000 Genomes Project. From a database hosting >500 million SVs, we focused on 351 genes where haploinsufficiency is a confirmed disease mechanism and identified 47 ultra-rare rearrangements that included an inversion (24 bp to 36.4 Mb, 20/47 *de novo*). Validation utilized a number of orthogonal approaches, including retrospective exome analysis. RNA-seq data supported the respective diagnoses for six participants. Phenotypic blending was apparent in four probands. Diagnostic odysseys were a common theme (>50 years for one individual), and targeted analysis for the specific gene had already been performed for 30% of these individuals but with no findings. We provide formal confirmation of a European founder origin for an intragenic *MSH2* inversion. For two individuals with complex SVs involving the *MECP2* mutational hotspot, ambiguous SV structures were resolved using long-read sequencing, influencing clinical interpretation. A *de novo* inversion of *HOXD11-13* was uncovered in a family with Kantaputra-type mesomelic dysplasia. Lastly, a complex translocation disrupting *APC* and involving nine rearranged segments confirmed a clinical diagnosis for three family members and resolved a conundrum for a sibling with a single polyp. Overall, inversions play a small but notable role in rare disease, likely explaining the etiology in around 1/750 families across heterogeneous clinical cohorts.

[1]Oxford Biomedical Research Centre, Centre for Human Genetics, University of Oxford, Oxford, UK; [2]Novo Nordisk Oxford Research Centre, Oxford, UK; [3]Genomics England, London, UK; [4]Translational Gastroenterology Unit, John Radcliffe Hospital, Oxford, UK; [5]School of Human Development and Health, Faculty of Medicine, University of Southampton, Southampton, UK; [6]Sheffield Institute for Translational Neuroscience, The University of Sheffield, Sheffield, UK; [7]Oxford Genetics Laboratories, Oxford University Hospitals NHS Foundation Trust, Oxford, UK; [8]Oxford Centre for Genomic Medicine, Oxford University Hospitals NHS Foundation Trust, Oxford, UK; [9]Department of Neurology, King's College Hospital, London, UK; [10]Manchester Centre for Genomic Medicine, Manchester University Hospitals NHS Foundation Trust, Health Innovation Manchester, Manchester, UK; [11]Wellcome Sanger Institute, Wellcome Genome Campus, Hinxton, UK; [12]Exeter Genomics Laboratory, Royal Devon University Healthcare NHS Foundation Trust, Exeter, UK; [13]Bristol Genetics Laboratory, North Bristol NHS Trust, Bristol, UK; [14]The All Wales Medical Genomics Service, University Hospital of Wales, Cardiff, UK; [15]North Thames Rare and Inherited Disease Laboratory, Great Ormond Street Hospital for Children NHS Foundation Trust, London, UK; [16]West Midlands Regional Genetics Laboratory, Central and South Genomic Laboratory Hub, Birmingham, UK; [17]Liverpool Centre for Genomic Medicine, Liverpool Women's NHS Foundation Trust, Liverpool, UK; [18]North East Thames Regional Genetic Service, Great Ormond Street Hospital for Children NHS Foundation Trust, London, UK; [19]Clinical Genetics Department, Guy's and St Thomas' NHS Foundation Trust, London, UK; [20]Department of Paediatric Neurology, Bristol Children's Hospital, Bristol, UK; [21]SW Thames Centre for Genomic Medicine, University of London & St George's University Hospitals NHS Foundation Trust, St George's, London, UK; [22]Department of Clinical Genetics, University Hospitals Bristol NHS Foundation Trust, Bristol, UK; [23]Clinical Genetics Group, MRC Weatherall Institute of Molecular Medicine, University of Oxford, Oxford, UK; [24]Grupo de Investigación en Biomedicina Molecular, Celular y Genómica, Unidad CIBERER (CB06/07/1030), Instituto de Investigación Sanitaria La Fe (IIS La Fe), Valencia, Spain; [25]Division of Evolution, Infection and Genomics, School of Biological Sciences, Faculty of Biology, Medicine and Health, University of Manchester, Manchester, UK; [26]Department of Clinical Genetics, Birmingham Women's and Children's NHS Foundation Trust, Birmingham, UK; [27]West of Scotland Centre for Genomic Medicine, Glasgow, UK; [28]Department of Clinical Genetics, Royal Devon University Healthcare NHS Trust, Exeter, UK; [29]Paediatric Neurosciences Department, Alder Hey Children's Hospital NHS Foundation Trust, Liverpool, UK; [30]Institute of Genetic Medicine, Newcastle University, International Centre for Life, Newcastle University, Newcastle, UK; [31]Canberra Clinical Genomics, Canberra Health Services and The Australian National University, Canberra, ACT, Australia; [32]Cancer Research, Canberra Hospital, Canberra, ACT, Australia; [33]Department of Clinical Genetics, Sheffield Children's NHS Foundation Trust, Sheffield, UK; [34]Sheffield Diagnostic Genetics Service, Sheffield Children's NHS Foundation Trust, North East and Yorkshire Genomic Laboratory Hub, Sheffield, UK; [35]Department of Cellular Pathology, Oxford University Hospitals NHS Foundation Trust, Oxford, UK; [36]Department of Oncology, University of Oxford, Oxford, UK
*Correspondence: alistair@well.ox.ac.uk (A.T.P.), jenny.taylor@well.ox.ac.uk (J.C.T.)
https://doi.org/10.1016/j.ajhg.2024.04.018.
© 2024 The Author(s). This is an open access article under the CC BY license (http://creativecommons.org/licenses/by/4.0/).

Introduction

Genomic inversions are segments of DNA where the sequence is present in the reverse orientation compared with the reference. In human populations, such rearrangements are spread all over the genome and have a wide range of sizes.[1] A range of complexity is typically seen,[2] and the mutational mechanisms underlying these types of rearrangement are becoming better understood.[3] Inversions differ in frequency, ranging from benign common polymorphisms to private *de novo* variants that predispose to disease. Inversions can cause disease through both loss- and gain-of-function mechanisms, the latter via creation of gene fusions or by changing regulatory landscape, which can lead to aberrant gene expression. Although inversions can be identified by traditional karyotyping approaches, such methods typically cannot identify rearrangements <10 Mb in size. Over the last 20 years, karyotyping has gradually been replaced by copy number variant (CNV) analysis using array-based testing or by multiplex ligation-dependent probe amplification (MLPA, Note S1). Initially, microarrays were constructed using large insert clones spaced at ~1-Mb intervals.[4] Gradual improvements in resolution/throughput combined with reductions in cost have meant that array testing has been adopted by the majority of clinical genetics laboratories, often as the first-line genetic test.[5] Therefore, although the rapid technical progress in array technologies has led to substantial improvements in diagnostic yields (and more generally in our understanding of the role of CNVs in human disease), copy-neutral structural variants (SVs), such as inversions, have in comparison been overlooked by clinical testing laboratories. Consequently, the relative importance of inversions and other types of balanced rearrangement remains elusive.

The UK's 100,000 Genomes Project (100kGP) was a large national study that aimed to uncover the genetic basis of disease for individuals with rare disease (RD) and cancer in whom a diagnosis had not been obtained by standard-of-care testing.[6,7] A secondary aim was to build upon evidence from previous studies[8] and demonstrate the feasibility of genome sequencing as part of standard clinical practice within the National Health Service (NHS). More background about the 100kGP is provided in Note S2. The diagnostic yield obtained across 2,183 families with RD in the initial pilot phase of the 100kGP was 25%.[6] However, this was only after significant input from the research community, which included the detection of 22 non-coding variants and a total of 40 SVs. Although SV calls from Manta[9] (a split-read variant caller) were reviewed, none of the 40 SVs were inversions or complex SVs. For the four duplications identified, it was also not reported whether these were direct tandem repeats or whether the additional DNA segments were in another configuration, as is typically seen in ~20% of cases.[10] These findings led us to question whether cryptic SVs (such as inversions) are ultra-rare in heterogeneous clinical cohorts such as the RD arm of the 100kGP, or if they are overlooked due to the SV filtering strategies employed.

Now that genome sequencing is more widespread, case reports describing inversions are becoming increasingly common in the literature.[11–14] Larger studies involving pre-selected cohorts (i.e., ascertainment due to the presence of a balanced cytogenetic abnormality) have shown that short-read genome sequencing can find the breakpoints for >90% of individuals, with direct gene disruption/imbalance playing a likely role in disease etiology for 27%.[15] However, to our knowledge there have been only limited systematic analyses performed across large unselected/heterogeneous RD cohorts. Thus, the overall impact of such variants in clinical settings is less certain.

In an earlier study using data from the main 100kGP program, we assessed the clinical impact of inversions by focusing on 43 genes linked to autosomal dominant musculoskeletal syndromes.[16] Presumed pathogenic inversions were detected in 10 individuals from three independent families. The aim of the present study was to extend our analysis to all 33,924 families from the 100kGP and across all RD areas by assessing an established set of genes that cause disease through haploinsufficiency (HI). As well as gaining a better understanding of the overall incidence of this class of variant on human disease, additional aims were to determine how commonly the inverted segments detected were part of more complex structural rearrangements (those that involve >2 breakpoints) and to make recommendations for the interpretation of such changes.

Material and methods

The main clinical analysis pipeline utilized as part of the 100kGP has focused largely on small variants called by Platypus.[17] Variant prioritization is based on gene sets derived from PanelApp,[18] a tool that uses crowdsourcing to gather expert knowledge and establish a consensus for high-quality diagnostic gene panels. For the majority of 100kGP participants, DNA was extracted from EDTA blood. Library preparation used the TruSeq PCR-free high-throughput kit, and sequencing was performed using 150-bp paired reads on a HiSeqX machine (Illumina). SVs were called using both Canvas v.1.3.1[19] and Manta v.0.28.0[9] and combined into a single structural vcf file. As Canvas detects only CNVs, Manta calls were utilized for the present study. Genomic data available for the 100kGP cohort comprise a mix of genome builds (GRCh37 and GRCh38). For the inversion-positive individuals identified in the present study, 12/47 were originally analyzed on GRCh37.

In order to prioritize SV calls from the 100kGP, we used SVRare[20] (web resources) and a MySQL database that hosted 554,060,126 SVs from 71,408 participants across 33,924 families with RD. Coordinates for the SVs called on the GRCh37 subset of genomes were converted to GRCh38 using the LiftOver tool (web resources). Clustering the SV calls using an overlap threshold set to 80% allowed us to filter for ultra-rare inversion calls detected by Manta. Large multiplex families are uncommon in

the 100kGP, and so we prioritized variants observed with an apparent allele count of 5 or less.

Rare inversions were filtered for those likely to disrupt a set of 351 genes for which HI is a well-established disease mechanism. This gene set was taken from the NIH-funded Clinical Genome Resource (HI = 3 genes, downloaded November 2022; Table S1 and web resources). At least one breakpoint had to lie within the gene region. Where both breakpoints lay inside the same gene, the inversion call had to span at least one coding exon, such that the SV would likely disrupt gene function. Larger inversions that invert an HI gene but leave it fully intact were deprioritized. GRCh38 gene coordinates were based on ENSEMBL release v.105. For higher confidence, we utilized "PairSupport" information available from the Manta output. One additional inversion was ascertained due to an unusual pattern of clustering for a filtered set of "TIERED" single-nucleotide variants (SNVs).

Detailed manual review of around 250 bioinformatically filtered inversions involved: (1) assessing whether the submitted diagnosis and associated Human Phenotype Ontology terms for the 100kGP participants were consistent with what was known about the disorder, based on OMIM and information from the Clinical Genome Resource; (2) visually reviewing 150-bp read alignments at the locus of interest using the Integrative Genome Viewer (IGV)[21] to determine whether the SV was likely genuine and how likely it was to disrupt gene function (especially for complex SVs); (3) assessing the mode of inheritance for probands where data for other family members were available; and (4) using the TIERING table (a list of prioritized small variants based on inheritance, population allele frequency, predicted consequence, and whether the gene in question is in the gene panel linked to the participant's condition) and exit questionnaire data (a table containing summaries of clinical laboratories' final interpretation of the reported variant) to determine whether the genetic cause for the participant's condition had already been uncovered.

Following manual review, all positive findings went through an internal review process at Genomics England, and approved submissions were then entered into the "Diagnostic Discovery" pathway for feedback of the result to one of seven genomic laboratory hubs (GLHs) for clinical assessment, validation, and onward reporting. A summary of the clinical tiering and the clinical triage process for researcher-identified variants has been described previously.[16] Data analysis was performed inside the Genomics England research environment, a secure virtual desktop environment that hosts up-to-date genomic and clinical data.

Long-read sequencing

Release 17 of the 100kGP data contains Pacific Biosciences (PacBio) genome sequencing data for 91 participants from the RD arm of the project as an example dataset to help demonstrate the utility of HiFi technology. A subset of these 91 participants had been proposed for inclusion due to the presence of a complex SV, especially those that involved duplicated segments and were consequently ambiguous based on short-read genome sequencing data. Thus, in the PacBio pilot data release, 9/91 individuals were identified as part of the present study. Long-read sequencing was performed on the Sequel IIe system (2–4 SMRT cells per sample). Data analysis was performed by PacBio using a pipeline that utilized pbmm2 v.1.9.0 for aligning reads to GRCh38, pbsv v.2.8.0 for SV calling, and DeepVariant v.1.4 for small variant calling. GLnexus v.1.4.1 was used for joint calling. Phasing was based on SNVs in reads rather than by inheritance. The reads that contain these variants were classified into haplotype group 1 or 2 based on the occurrence of shared variants by WhatsHap v.1.0.[22] Specific long-read sequences from the locus of interest were compared to the GRCh38 reference with FlexiDot[23] using the settings wordsize = 50 and –plotting_mode = 1.

Transcriptomics

For a subset of individuals entered into the RD domain of the 100kGP, RNA was collected at the time of recruitment using PaxGene blood tubes. For individuals that remained unsolved, RNA-seq was performed. Following a standard RNA extraction procedure, whole-blood RNA samples were depleted for rRNA and globin. Samples were sequenced by Illumina using 100-bp paired-end reads, with a mean of 109 million mapped RNA-seq reads per individual. Alignment and transcript quantification were performed using Illumina's DRAGEN pipeline (v.3.8.4). Expression outlier analysis was performed using OUTRIDER,[24] which was run via the DROP pipeline (v.1.3.3)[25] using default settings in batches of 500 individuals. By chance, the probands from several families harboring inversions reported here had been included in this RNA-seq cohort, allowing us to assess whether the observed SVs impacted gene expression.

Haplotype sharing analysis and mutation age estimation

Locus-specific joint variant calling was performed using Platypus v.0.7.9.5 and the GRCh38 reference. Default settings were employed, except for minFlank = 0. We filtered for PASS variants, removed indels, and then calculated the absolute difference in B allele frequency (i.e., number of reads containing variant divided by number of reads covering variant). These values were plotted against genomic position using the ggplot package[26] in R (web resources), in order to identify regions where there was an absence of conflicting homozygosity. SNPs flanking the shared haplotype were assessed in IGV. Coordinates of the shared haplotype were converted to hg19 using the LiftOver tool and then run on the R Shiny app "Genetic Mutation Age Estimator." This is a widely used online tool that can be reliably applied to small numbers of samples and uses a method for estimating the age of a mutation based on the genetic length of ancestral haplotypes shared between individuals carrying the mutation.[27–29]

Ethics declaration

Ethics approval for 100kGP was from Cambridge South REC (14/EE/1112), and consent included a statement that "my samples can be used for collecting DNA for whole-genome sequencing." Approval for ongoing PacBio studies on Family 40 was from the North West 7–Greater Manchester Central Research Ethics Committee (10/H1008/74).

Results

Overall results summary

A total of 62 affected individuals from 47 independent families harboring 46 different SVs were detected on account of a MantaINV call (Table S2). For all these variants, "Researcher identified potential diagnosis" forms were submitted to be considered for entry to the diagnostic discovery pathway. Requests to contact the recruiting clinicians were made concurrently. Overall, six genes were hit twice, and these included: *MSH2* (exemplar 1, MIM: 609309), *MECP2* (exemplar 2, MIM: 300005), *GLI3*

(MIM: 165240), *AUTS2* (MIM: 607270), *CDKL5* (MIM: 300203), and *ARID2* (MIM: 609539). For 14/47 families (30%), the gene found to be disrupted had been strongly suspected, as evidenced by previous targeted sequencing or MLPA testing (Table S2).

The size of the inversions ranged over six orders of magnitude, with the largest two above 30 Mb in size (Figure 1A). The mean and median sizes were 3.51 Mb and 635 kb, respectively. The largest inversion was a *de novo* 36.4-Mb variant (chrX:18,472,998–54,910,892, GRCh38) that disrupts *CDKL5* in an individual with intellectual disability and seizures, consistent with a diagnosis of developmental and epileptic encephalopathy 2 (MIM: 300672). The second largest SV was a 30.7-Mb inversion with a proximal breakpoint in intron 13 of *MLH1* (GenBank: NM_000249.4; MIM: 120436). This variant had been identified in parallel with the 100kGP by karyotyping and has now been confirmed by fluorescence *in situ* hybridization (FISH) (Figure 1B). This individual (Family 19) had been recruited to 100kGP due to early-onset colorectal cancer (CRC, aged 27 years old), and this was assumed to be a cryptic form of Lynch syndrome due to the significant family history (Figure S1). Detection of the inversion resolved the diagnosis to Lynch syndrome 2 (MIM: 609310).

For 87% of individuals (41/47), the inversion was below 10 Mb in size, which is considered the typical resolution for being able to detect an inversion by karyotyping (Figure 1A). The smallest inversion involved a 24-bp segment in *NUS1* (GenBank: NM_138459.5; MIM: 610463), which inverts the splice acceptor site at the start of exon 3 (Figures 1C and S2). Notably, this was the only SV identified using the small variant caller. It was not called as an inversion but detected due to an unusual clustering of tiered SNVs. This inversion was seen in a male (Family 45) with slowly progressive myoclonic epilepsy, ataxia, and mild intellectual disability, and overall his phenotype was considered a good match to previous reports of intellectual developmental disorder, autosomal dominant 55, with seizures (MIM: 617831).[30] Prior testing had included assessment of repeat expansions in *HTT* (MIM: 613004)/ *ATN1* (MIM: 607462), a screen for common mtDNA mutations, and *POLG* (MIM: 174763) sequencing.

Across the 47 families, 20 of the variants were confirmed to have arisen *de novo*, and in 11 families, the variant was inherited from an affected parent (Figure 1D). Support from co-segregation was strongest for two previously reported quad families with inversions that disrupt *GLI3* and result in clinical features consistent with Greig cephalopolysyndactyly syndrome (MIM: 175700).[16] Other families from this cohort described previously include individuals with SVs that disrupt *KMT2E* (MIM: 608444),[31] *FBN1* (MIM: 134797),[16] and *TWIST1* (MIM: 601622).[32]

Different categories of SVs detected due to MantaINV call

Although all SVs were detected on account of a MantaINV call, there was a wide range of complexity seen across the cohort. Different categories of SV types identified are listed in full in Table S2 and summarized in Figure 1D.

Simple inversions

Overall, 24/47 SVs were relatively simple inversions with <10% loss at one end. An illustrative example of this class of SV was a 100kGP participant with lissencephaly 1 (MIM: 607432) harboring a 256-kb inversion (Figure S3) disrupting *PAFAH1B1* (MIM: 601545; Family 13).

Inversions with loss (>10%) at one end

Inversions with a significant loss (>10%) just at one end were seen in a further three individuals. Examples of this category include one boy with a phenotype consistent with Wiedemann-Steiner syndrome (MIM: 605130) in whom a *de novo* inversion/loss disrupting *KMT2A* (MIM: 159555) was detected (Family 5). Another individual with syndromic cleft palate (Family 10) harbored an inversion disrupting *MEIS2* (MIM: 601740), a gene linked to cleft palate, cardiac defects, and impaired intellectual development (MIM: 600987).

Deletions with small internal segment(s) retained

Some SVs picked up due to MantaINV calls were categorized as complex deletions with retained/inverted internal segments. Four such rearrangements were detected, which included Family 21, where a *de novo* SV disrupting *KMT2E* was identified in a female with delayed developmental milestones, autistic traits, postnatal macrocephaly, and tall stature, consistent with a diagnosis of O'Donnell-Luria-Rodan syndrome (MIM: 618512).[31] A complex deletion involving *EFTUD2* (MIM: 603892; Family 12), identified in an individual with a blended phenotype (Note S3), also fitted into this subgroup. An adult male with multiple renal and hepatic cysts harbored a similar complex deletion involving *PKD1* (MIM: 601313; Family 11), while another participant recruited due to intellectual disability and short stature (Family 14) carried a similar *de novo* rearrangement involving *KMT2B* (MIM: 606834).

Duplication-triplications

Another class of rearrangement, observed in two families, was the duplication-triplication structure. Although this type of SV appears complex, only two breakpoints are involved. Such SVs are also known as "Carvalho" type rearrangements[33] and can be hard to resolve, as four configurations can be possible. In this study, we identified only one such rearrangement with an unambiguous effect on gene function. This duplication-triplication involved *COL4A5* (MIM: 303630) and was found in two affected family members recruited to 100kGP with familial hematuria (Family 25). The SV was prioritized due to the 7-kb MantaINV call (exons 4–6 based on GenBank: NM_033380.3), which corresponds to the duplicated region at the proximal end of the SV (Figure S4). Inverted Alu elements present at the distal end of this rearrangement are notable given that duplication-triplication/inverted duplications are thought to often be mediated by pairs of inverted low-copy repeats.[34] As the SV was fully intragenic to *COL4A5* and involves multiple exons, it would likely disrupt gene function regardless of which configuration is correct and

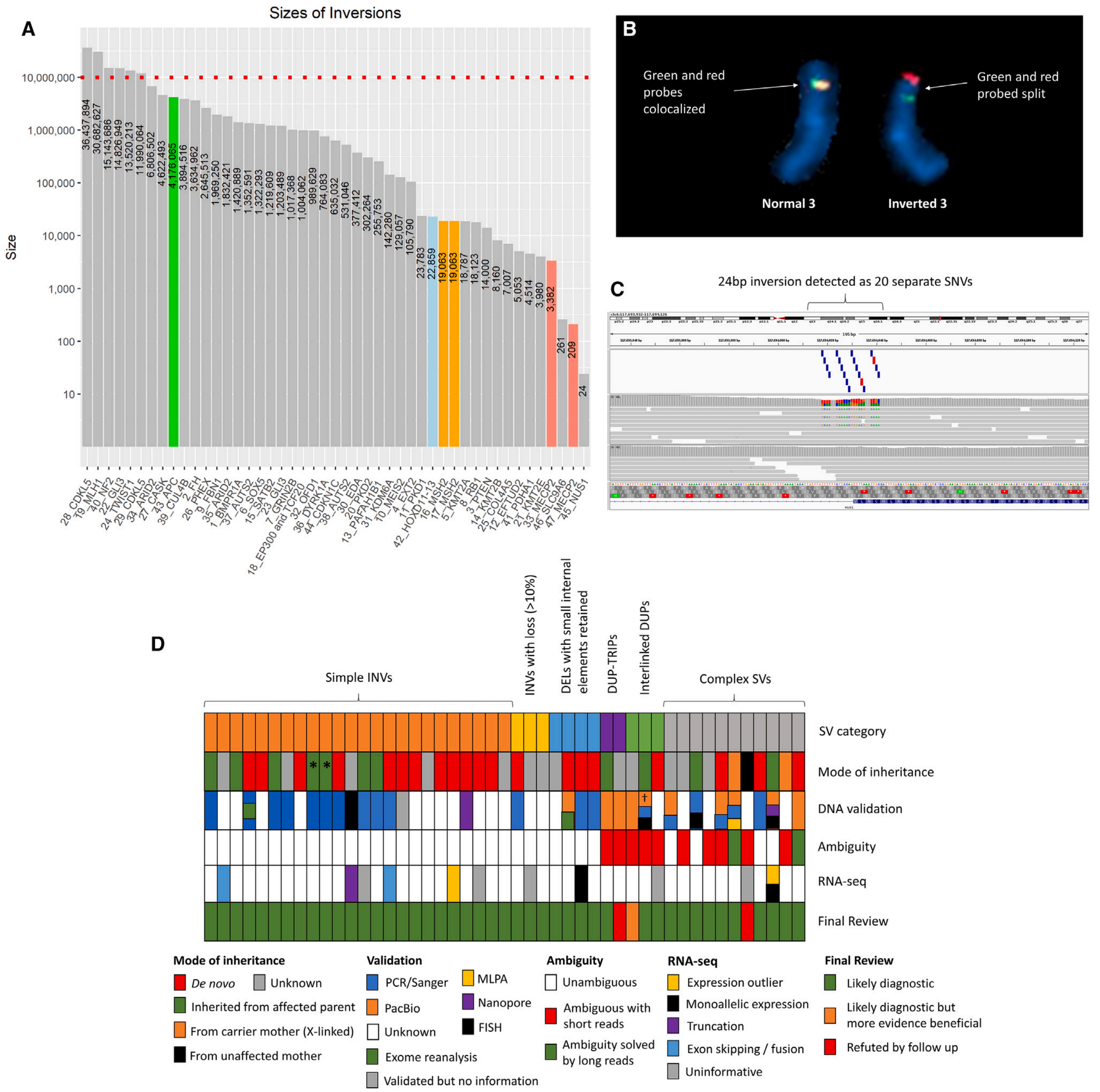

Figure 1. Size range and summary of detected inversions

(A) Size distribution of inverted genomic segments in 47 families from the 100,000 Genomes Project. For complex SVs, the largest of the MantaINV calls is plotted. Family ID and gene symbols are shown in x axis labels. The dotted red line represents a size threshold of 10 Mb, the typical limit below which karyotyping is unlikely to detect an inversion. Exemplars are highlighted in orange (*MSH2*), salmon (*MECP2*), blue (*HOXD*11-13 cluster), and green (*APC*).

(B) FISH images for metaphase spread showing normal and inverted chromosome 3, where commercial break-apart probes confirm disruption of *MLH1* in the proband from Family 19. Red and green probes hybridize adjacent to the 5′ and 3′ ends of *MLH1*, respectively.

(C) Data supporting 24-bp inversion (c.542−13_552inv [GenBank: NM_138459.5]) involving exon 3 of *NUS1* seen in an individual with epilepsy. GRCh38 coordinates are chr6:117,694,018–117,694,041. Upper track shows 20 "SNVs" called by Platypus, of which 15 had a predicted consequence type prioritized by the interpretation pipeline (2 stop-gain[highlighted in red], 2 splice acceptor, 5 missense, 6 splice region). *NUS1* was not on Genetic Epilepsy syndromes (v.1.13) or intellectual disability (v.2.597) panels applied at initial analysis, and so these variants were flagged as TIER3.[16] Middle track shows alignments from the proband in Family 45, and lower track are alignments for a control subject sequenced in the same batch. This variant was not detected by Manta or by using the Illumina small variant caller.

(D) Summary of SV type, inheritance pattern, validation status, structural ambiguity, RNA-seq data availability, and final assessment across all 47 families. Order of families is identical to Table S2. *For 2/10 families (Families 16 and 7), inheritance is inferred from haplotype studies due to the *MSH2* inversion being a founder variant. †PacBio analysis for Family 40 is ongoing.

so is consistent with a diagnosis of Alport syndrome 1, X-linked (MIM: 301050). A second duplication-triplication involving *PDHA1* (MIM: 300502; GenBank: NM_000284.4) in Family 41, prioritized due to a 4.5-kb inversion call that involved exons 6–9 (Figure S5), was harder to interpret (Note S4).

Interlinked duplications

Although most rare duplications are arranged in a direct tandem-repeat orientation, ~20% of the time there is a more complex structure.[10] Such rearrangements can also result in MantaINV calls, Family 44 being a good example. The proband was recruited to the 100kGP with a diagnosis of classical Beckwith-Wiedemann syndrome (MIM: 130650). Two Manta inversion calls of 635 kb and 334 kb were detected that lay close to *CDKN1C* (MIM: 600856; Figure S6). Closer scrutiny of the short-read data indicated that this *de novo* SV involved three interlinked duplications of 15–63 kb (Figure S7). Three possible configurations could explain the short-read data (Figure S8), only one of which lay within *KCNQ1* (MIM: 607542), which would likely disrupt methylation of the imprinting control region 2 (ICR2). To help resolve this structural ambiguity, the family was offered testing by Bionano optical mapping on a research basis, but declined. Despite having only short-read data for this family, phasing the *de novo* SV was possible using four informative SNPs that lie close to breakpoints (Figure S9). The SV was shown to have occurred on the maternal chromosome, which fits with the imprinted nature of this locus and the fact that *CDKN1C* is normally expressed exclusively from the maternal chromosome.[35] A similar pattern of three interlinked duplications was observed in Family 31 (*KDM6A*, MIM: 300128) and Family 40 (*NF2*, MIM: 607379), described below.

Other types of complex SV

Other complex SVs were identified that did not fit into discrete subtypes. One such SV was identified in a female proband (Family 30) recruited to 100kGP due to a clinical suspicion of ectodermal dysplasia. Other features included bilateral talipes (from birth), conical teeth, and slightly fine scalp hair. Height and head circumference were within the normal range. Nails were noted to be slightly thin, especially those of her great toes. Overall, there were clear though mild features of ectodermal dysplasia, and *EDAR* (MIM: 604095) variants had been excluded prior to recruitment to the 100kGP. A resemblance to the X-linked hypohydrotic ectodermal dysplasia form (MIM: 305100) was noted, despite episodes of heat intolerance not being reported. Two MantaINV calls of 377 kb and 340 kb were identified, each with a breakpoint lying in intron 1 of *EDA* (MIM: 300451). Closer scrutiny of the data showed that the rearrangement involved two non-tandem duplications that had been inserted into *EDA* at the site of a 42-kb deletion (Figures S10A and S10B). The rearrangement was ambiguous with short-read data, with three possible configurations (Figure S10C), although all would likely be disruptive for *EDA* function. The SV was also shown to have arisen *de novo* and occurred on the paternal chromosome, a finding that could be of value if accurate recurrent risk estimates are needed.[36]

Another unusual SV was identified in Family 39, where two immediately adjacent inversions of 3.9 Mb and 1.4 Mb had been detected. The middle of the three breakpoints disrupted intron 20 of *CUL4B* (MIM: 300304; GenBank: NM_003588.4) (Figure S11). This boy had been recruited to the DDD study[37] (which did not pick up any likely pathogenic variants) and then to the 100kGP, with a diagnosis of intellectual disability, severe global delay, and seizures. His phenotype was considered to be consistent with "intellectual developmental disorder, X-linked syndromic, Cabezas type" (MIM: 300354). Although the father had been recruited to the 100kGP coded as affected, from the limited information available his condition appeared to be much less severe than that seen in his son. Given the *de novo* disruption of *CUL4B*, it may now be appropriate for the genome sequencing data for the father to be re-examined as a singleton, to search for an alternative genetic diagnosis.

The most complex SV in the present study was the translocation involving chromosomes 5 and 11, with nine rearranged segments (Family 43) that disrupted *APC* (MIM: 611731). This rearrangement and how it resolved a clinical dilemma is described below (exemplar 4).

Validation status

Our analysis was performed on a clinical cohort spread across the UK and involving many different GLHs. The experience and resources available for validation and reporting of complex SVs from the 100kGP varied across these GLHs. These efforts are therefore ongoing, and data on these aspects are incomplete. To the best of our knowledge, ~18 months after reporting this set of variants, 28/47 (60%) of the SVs reported here have now been confirmed at the DNA level, with a range of orthogonal approaches (Tables S2 and S3). The most commonly used approach was PCR/Sanger sequencing, which was undertaken in 17/47. For some individuals, more than one method was used (Figure 1D).

Retrospective analysis of exomes

For six families (6, 12, 29, 36, 37, and 39), exome sequencing had been performed previously as part of the DDD study,[37] and read alignment data were available for review. For 2/6 participants, retrospective analysis of these exome data was able to validate the SV. The first of these was the individual with a blended phenotype (Family 12, Note S3). The complex SV involving *EFTUD2* (GenBank: NM_004247.4) removes exons 3–6 and partially deletes exon 7. As the exon 7 breakpoint lies right in the middle of the exon (Figures S12A and S12B), it is not surprising that it was captured by the exome data (Figure S12C).

The second participant where exome data could be used to confirm an inversion was a female recruited to 100kGP due to severe intellectual disability seizures (Family 6). Before the DDD study/100kGP, prior testing had included

TCF4 (MIM: 602272) and array-CGH. We identified a *de novo* 1.3-Mb inversion with a proximal breakpoint lying in intron 3 of *SOX5* (MIM: 604975; GenBank: NM_006940.6), which resolved the diagnosis to that of Lamb-Shaffer syndrome (MIM: 616803).[38] Although that breakpoint lay almost 400 bp from the exon boundary and despite only 5–6× coverage at the breakpoint, we identified one read mapping to the distal end of the inversion that contained an inverted sequence from the proximal end (Figure S13). This helped confirm the findings from the genome sequencing data and resolved a 41-year odyssey. Clinical utility extends to the brother, as we can now confirm low recurrence risk to his offspring.

Array testing

Three complex SVs involving duplicated segments had already been identified by array testing prior to 100kGP recruitment, although the additional complexity had not previously been realized. This included the duplication-triplication involving *PDHA1* (Family 41, described below), a participant with three interlinked duplications involving *KDM6A* (Family 31), and a rare 721-kb duplication in a female with suspected Rett syndrome (Family 29), which was assumed to be a tandem event but which the sequence data show has been inserted into *CDKL5*.

Long-read genome sequencing

Of the 10/47 individuals followed up using long-read genome sequencing, nine had been included in Genomics England's pilot study using PacBio HiFi technology. For another participant, long-read genome sequencing was performed independently with nanopore sequencing. In every instance, all SV breakpoints were validated, with no additional complexity being identified.

In 4/10 individuals where the structure of the SVs had been interpreted as unambiguous from short-read data, long-read data helped confirm the prior SV interpretation. These included Family 43 with a complex but copy-neutral translocation disrupting *APC* (exemplar 4), Family 12 described above (*EFTUD2*), and Family 2 with a translocated inversion disrupting *FH* (MIM: 136850) (described below). For Family 37, unsolved following the Scottish Genomes Partnership's analysis of 100kGP short-read data,[39] a *de novo* balanced 1.35-Mb inversion disrupting *AUTS2* was identified as part of the present study and subsequently validated with nanopore genome sequencing (to be described elsewhere).

In contrast, for the remaining 6/10 participants where long-read data were available, there had been ambiguity regarding the precise SV configuration based on short-read data alone, due to duplicated segments. For two of these, the SV has now been resolved due to the long-read data. This includes two individuals (Families 33 and 47) with complex SVs involving the final exon of *MECP2*, where PacBio data resolved the SVs, and these findings directly influenced clinical interpretation. These results are described in more detail below (exemplar 2). The four remaining complex SVs could not be resolved with PacBio data. This was due to the presence of large duplicated segments that could not be spanned by HiFi reads, which typically extend to just above 20 kb in length. These included complex SVs in Families 25, 30, 31, and 41, involving *COL4A5*, *EDA*, *KDM6A*, and *PDHA1*, respectively. In the future, we anticipate that these latter SVs could be investigated using Bionano optical genome mapping or by using long-read sequencing approaches, where library preparation methods are optimized to achieve ultra-long DNA fragments.

Support from RNA-seq studies

RNA-seq data were available for 11/47 participants, and in six, these data helped further support pathogenicity (Table 1; Figure 1D). Most notable among these was Family 36, where the proximal breakpoint of a 764-kb inversion (Figure S14) lay in intron 1 of *DYRK1A* (MIM: 600855; GenBank: NM_001347721.2). This individual was recruited to the 100kGP with a diagnosis of intellectual disability, but additional features included deep-set eyes, a prominent nasal bridge, short stature, hirsutism, and epilepsy. There was significant microcephaly (between -4 and -5 SDs), and MRI investigations demonstrated delayed myelination, especially subcortical white matter atrophy, pons, and cerebellum. The SV had arisen *de novo*, and the clinical features were thought to be consistent with intellectual developmental disorder, autosomal dominant 7 (MIM: 614104). However, because the translation start site lies in exon 2, the coding sequence is intact, and thus some diagnostic uncertainty remained. We speculated that the dislocation of the evolutionarily conserved 5′ UTR exon from the remainder of the gene would result in reduced expression. There were no informative coding SNPs, and so allelic imbalance analysis was not possible. However, OUTRIDER analysis indicated a 0.57-fold change in expression (adjusted p value 3.7×10^{-27}), helping confirm our hypothesis.

RNA-seq data were available for an individual (Family 26) with suspected hypophosphatemic rickets (MIM: 307800) in whom a *de novo* 2.6-Mb inversion involving *PHEX* (MIM: 300550; GenBank: NM_000444.6) had been identified. Given the position of the proximal breakpoint in intron 15 (Figure S15), this inversion is highly likely to disrupt gene function. Unfortunately, the low expression in blood (e.g., TPM = 0.03 in GTEx) and low data quality for this subject (only 35M mapped RNA-seq reads) meant that expression analysis was uninformative for *PHEX*. However, the distal breakpoint for this inversion falls within *SH3KBP1* (MIM: 300374), and OUTRIDER did detect a significant decrease of the expression of this gene (0.46-fold change, adjusted p value 6.6×10^{-21}), supporting the overall deleterious nature of this event. In two other individuals, heterozygous coding SNPs were used to demonstrate monoallelic expression (Figures 2A and S16), whereas for the *PTEN* (MIM: 601728) inversion described below, in-frame skipping of exons 6–8 was observed (Figure 2B).

In Family 35, we initially reported a 1.8-Mb inversion involving *ARID2* in a male with intellectual disability and delayed motor development. Although inherited

Table 1. **Summary of six 100kGP participants with inversions that are supported by RNA-seq data**

Family	GRCh38 coordinates of inversion	Segment size (bp)	Imbalance/complexity	Affected family members (structure, inheritance)	Gene (median blood TPM)	Recruitment diagnosis	RNA-seq result	Additional comments
3	chr10:87,949,156–87,963,156	14,000	N/A	1 (singleton, unknown)	*PTEN* (40.27)	genodermatoses with malignancies	skipping of exons 6–8 supported by 38 reads/read pairs (Figure 2B); OUTRIDER expression not an outlier	consistent with the inversion of exons 6–8 of this 9-exon gene (GenBank: NM_000314.8, Figure S22); NMD not expected as reading frame maintained and prediction is of 178 deleted amino acids (p.Gly165_Lys342del [c.493_1026del]).
14	chr19:35,717,452–35,725,612	8,160	deletion of 8,185 bp (chr19:35,717,427–35,725,612) and 1 retained/inverted internal segment of 94 bp at proximal end (chr19:35,717,452–35,717,546)	1 (trio, *de novo*)	*KMT2B* (21.37)	intellectual disability	monoallelic expression for rs11670414 (T, maternal) and rs231591 (G, unphased); SV occurred on paternal chromosome; variant read support in RNA data is 47T/0C and 13G/0A (Figure 2A)	breakpoint delineating end of deleted region in exon 12 of this 37-exon gene (GenBank: NM_014727.3); coding SNPs in exons 16 and 30; OUTRIDER expression data unavailable
19	chr3:6,352,714–37,035,341	30,682,627	N/A	1 (singleton, unknown)	*MLH1* (6.61)	familial colon cancer (Figure S1)	although OUTRIDER expression is not an outlier, manual review indicates reduced expression after exon 13 (breakpoint in intron 13)	there is transcript annotation ending in *MLH1* exon 13 (ENST00000674107.1), potentially explaining the stability of this truncated transcript; manual review suggests fusion transcript
26	chrX:19,564,733–22,210,246	2,645,513	11/12 bp lost at each end	1 (trio, *de novo*)	*PHEX* (0.03)	renal tract calcification	OUTRIDER expression uninformative for *PHEX*; distal breakpoint disrupts *SH3KBP1* (GTEx TPM = 49.34), for which OUTRIDER did detect a decrease of expression (0.46-FC, padj 6.6×10^{-21})	*SH3KBP1-PHEX* fusion transcript detected by DRAGEN (Figure S15)[a]; *PHEX* expression elevated at 3′ end after breakpoint
36	chr21:37,377,983–38,142,066	764,083	N/A	1 (trio, *de novo*)	*DYRK1A* (7.42)	intellectual disability	OUTRIDER expression analysis shows a 0.57-FC, with padj 3.7×10^{-27}	breakpoint is in intron 1, and exon 1 is non coding 5′ UTR; therefore impact confirmed only with RNA data; however, truncation in this intron was reported previously in case with translocation[40]

(*Continued on next page*)

Table 1. *Continued*

Family	GRCh38 coordinates of inversion	Segment size (bp)	Imbalance/complexity	Affected family members (structure, inheritance)	Gene (median blood TPM)	Recruitment diagnosis	RNA-seq result	Additional comments
43	chr5:112,769,977–116,946,042	4,176,065	11.9-kb deletion (chr5:111,440,257–111,452,111), but otherwise this complex translocation involving 9 rearranged segments is balanced (Figure 6E)	3 (father and 2 sons, from affected parent)	*APC* (1.82)	multiple bowel polyps (Figures 6B and 6C)	OUTRIDER expression analysis shows a 0.65 FC, with padj 1.5×10^{-10}; monoallelic expression confirmed using six-SNP haplotype (rs2229992-rs351771-rs411115-rs42427-rs866006-rs465899; C-A-A-A-G-A; Figure S16); haplotype phasing with PacBio	OUTRIDER also detected significant downregulation of *CAMK4* (FC = 0.53, padj = 2.0×10^{-3}), which is disrupted directly (Figure 6D); downregulation of *DCP2* (FC = 0.75, padj = 2.7×10^{-5}), which lies 207 kb from the *APC* breakpoint, highlights potential for complex SVs to result in long-distance position effects

[a]Comparison of genomic and fusion transcript breakpoint available in UCSC session https://genome.ucsc.edu/s/AlistairP/PHEX_INV_RNA_coords. TPM, transcript per million (data obtained from GTEx v.8); DDD, Deciphering Developmental Disorders Study (www.ddduk.org); FC, fold change, padj, adjusted *p* value.

from his apparently unaffected mother, a recent report noted variable expressivity linked to a nonsense variant in this gene.[41] We therefore considered that incomplete penetrance could be plausible, and our initial interpretation was that the variant was likely to lead to gene disruption. However, closer scrutiny of read alignments supporting the MantaINV call (Figure S17), together with input from the diagnostic discovery review team, suggested that a more parsimonious explanation was that the SV was an inverted AluSc element integrated into intron 16 (GenBank: NM_152641.4). Long-read sequencing data were not available to confirm this alternative explanation, and RNA-seq did not obtain enough reads for OUTRIDER analysis to be possible.

Final yield of likely diagnostic SVs

In Family 41, the complex duplication-triplication involving *PDHA1* (mentioned above) was identified in a participant with exercise intolerance, intellectual disability, and white matter abnormalities and so compatible with pyruvate dehydrogenase E1-alpha deficiency (MIM: 312170). Near-normal enzyme activity and immunohistochemistry results together suggest that the duplication likely has no consequences (Note S4). Discounting this and the participant with the *ARID2* variant of uncertain significance (Family 35) takes the overall incidence of likely diagnoses linked to this class of SVs in the set of prioritized genes to 1/754 (i.e., 45 across the 33,924 families contained in the SVRare database). This reported incidence will have been influenced by the degree of prior testing performed before families were recruited to the 100kGP. For many disease areas, ruling out the most likely candidate gene(s) was a prerequisite for enrollment, and in the RD pilot, a median of 1 genetic test (range 0–16) had been undertaken.[6] A similar analysis of complex SVs using genome sequencing data as a first-line test would likely result in a lower incidence of such SVs, as the cohort would still contain many cases with non-cryptic variants in strong candidate genes. Such an ascertainment bias might be particularly strong for familial cancer syndromes where there are well-established lists of candidate genes and the current standard-of-care testing is typically extensive. The wider clinical impact of inversions across the full complement of human genes is also unknown (see discussion).

Clinical impacts

100kGP participants with blended phenotypes

Several clinical exome sequencing studies have highlighted that 4.6%–4.9% of diagnoses represent blended phenotypes due to variants in more than one gene.[42,43] In the cohort described here, there were four families where we propose that individuals express complex phenotypes due to involvement of two different genes. In two individuals, the genes involved lie at each end of the inversion, whereas the other two affected individuals harbor a second independent pathogenic SNV, unrelated to the inversion.

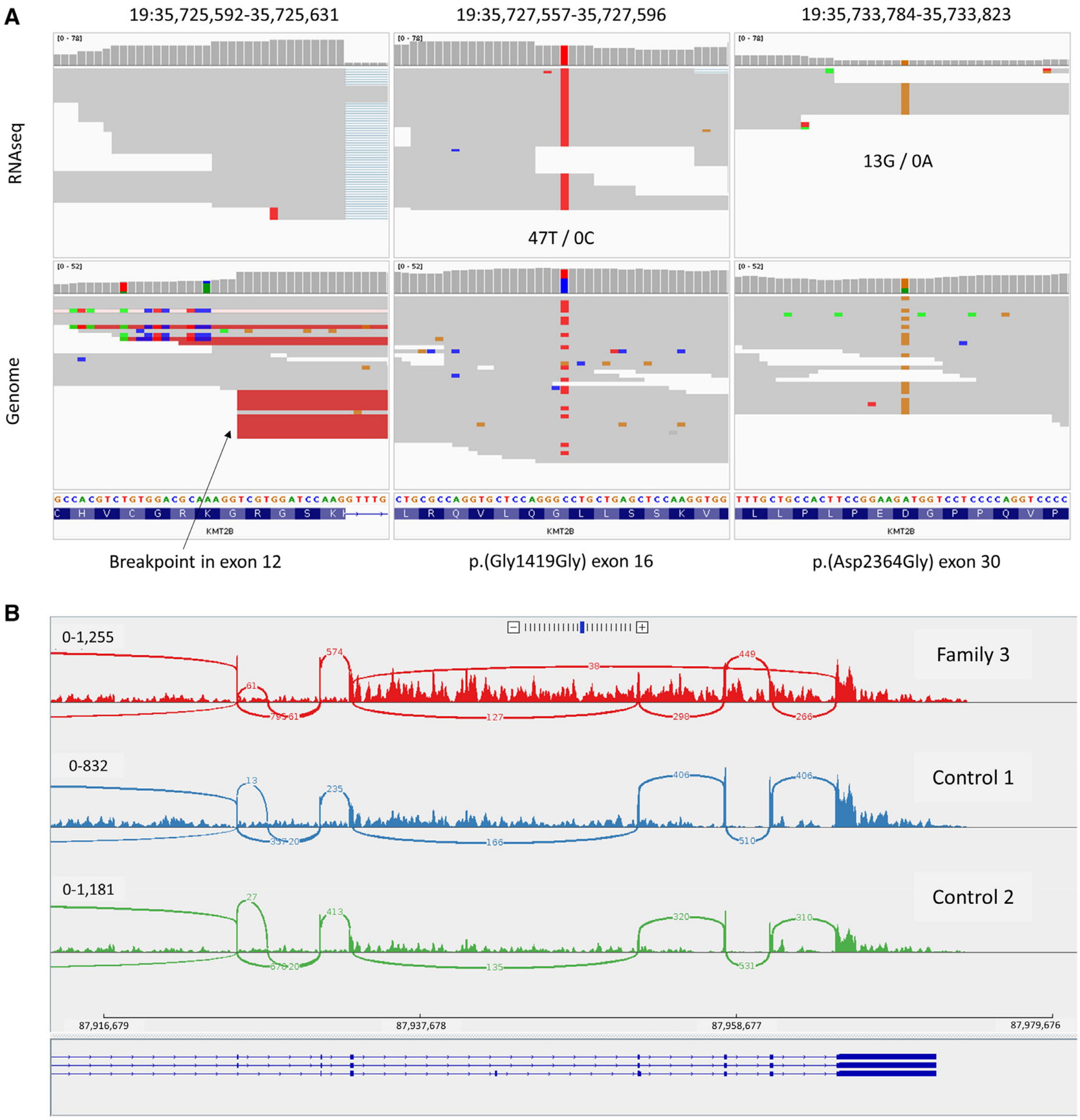

Figure 2. RNA-seq data for Families 14 and 3 showing examples of allele imbalance and exon skipping
(A) *De novo* deletion/inversion in Family 14 results in monoallelic expression of *KMT2B* (GenBank: NM_014727.3). RNA-seq data for the proband (upper track) is compared to the genome sequencing data (lower). Monoallelic expression is apparent for two common SNPs in exons 16 and 30, rs11670414 (T allele, phased as maternal by inheritance) and rs231591 (G allele, not possible to phase by inheritance as both parents are heterozygous). Both c.4257C>T (p.Gly1419=) and c.7091A>G (p.Asp2364Gly) are common SNPs and have been assessed as benign in multiple submissions to ClinVar (VCV001230475.12; VCV001262833.10).
(B) Sashimi plot showing that the inversion in Family 3 leads to skipping of *PTEN* exons 6–8. Viewing settings are minimum ten reads, and only junctions in the forward direction are shown. There are 38 reads/read pairs that support the exon 5–9 junction, and this pattern is not seen in two other representative control RNA-seq datasets analyzed using an identical pipeline. Genome sequencing data for this proband are shown in Figure S22, and the inversion involves the same three exons. The HGVS annotation and predicted consequence of this change is therefore c.493_1026del (GenBank: NM_000314.8) (p.Gly165_Lys342del). In-frame skipping would not be expected to activate the NMD process and explains the normal OUTRIDER expression results seen for this gene in this individual.

The first participant with a suspected blended phenotype involves *GLI3* and *HOXA13* (MIM: 142959; Family 22, described previously[16]). The 14.8-Mb inversion disrupts *GLI3*, and the affected individuals had features consistent with Greig cephalopolysyndactyly syndrome. However, several other features were more in keeping with hand-foot-genital syndrome (MIM: 140000), and prior testing had focused on *HOXA13*. The distal breakpoint of the inversion lay ~45 kb upstream of *HOXA13*. Positional effects for *HOXA13* have been proposed to act over much longer distances.[44]

Another participant with a suspected blended phenotype was identified, and this involved a *de novo* inversion on 22q13.2 in a girl (Family 18) recruited to 100kGP due to ocular coloboma and an abnormality of ocular abduction. As well as visual impairment, the participant had

mild global developmental delay, kyphoscoliosis, morphological abnormality of the semi-circular canal, and syndactyly. The proximal and distal breakpoints of this 1.0-Mb inversion disrupt *EP300* (MIM: 602700) and *TCF20* (MIM: 603107), respectively (Figure S18). As these are both well-established OMIM morbidity genes, and both are intolerant to loss-of-function variations (pLI = 1), we considered that the individual's phenotype could be due to HI of both genes. At the time of entry into the 100kGP, the individual was too young for it to be clear whether typical features of Rubinstein-Taybi syndrome 2 (MIM: 613684) were present, but subsequent assessment has confirmed typical facial features and broad hallux.

Family 4 was recruited to the 100kGP as a parent-child trio, the proband and father with a diagnosis of ear malformations and hearing impairment. This family was already considered to be solved, due to an inherited likely pathogenic c.662A>G (p.Asn221Ser) (GenBank: NM_000248.4) variant in *MITF* (MIM: 156845), a gene associated with Waardenburg syndrome, type 2A (MIM: 193510). However, bone exostoses were an additional complication in both affected individuals, and this feature is not typically observed in individuals with Waardenburg syndrome. Both individuals shared a 106-kb inversion (Figure S19) that involves exons 2–10 of the 14-exon *EXT2* gene (MIM: 608210; GenBank: NM_207122.2) and hence is highly likely to disrupt gene function and result in the bone phenotype. This finding will facilitate genetic testing of an affected brother (not in the 100kGP) who has multiple exostoses, but not deafness. Confirmation of the inversion in this individual would help delineate the components of this blended phenotype. An additional example of a blended phenotype involving *EFTUD2* (Figure S12) and an inherited missense variant in *FGFR3* (MIM: 134934) is described in Note S3.

Inversions disrupting tumor suppressor genes

A notable subset of the cohort (10/47 families) harbored germline inversions that disrupted tumor suppressor genes and resulted in well-known cancer susceptibility type conditions.

The 30.7-Mb inversion disrupting *MLH1* (described above) will facilitate cascade testing in the 100kGP participant's son and other at-risk family members in this large kindred. Positive history for cancer had gone back at least three generations and included an affected brother (CRC, 28 years old) and paternal uncle (CRC, 42 years old; Figure S1). Microsatellite instability had also been confirmed in two family members. Although immunohistochemistry indicated loss of MLH1, previous germline testing had not yielded a pathogenic variant. Original genome sequence data, generated in March 2016, were initially analyzed using build GRCh37, and the 100kGP clinical pipeline had flagged only c.86G>C (p.Gly29Ala) (GenBank: NM_000535.7) in *PMS2* (MIM: 600259), which is likely benign (VCV000041721.56).

The proband in Family 1 was a young female with a classical juvenile polyposis phenotype (MIM: 174900) who first presented with intussusception at 7 years. Recent colonoscopies demonstrated juvenile polyps. Targeted testing of *SMAD4* (MIM: 600993) and *BMPR1A* (MIM: 601299) had not identified any candidate germline susceptibility variants, and therefore the individual was recruited to the 100kGP together with her similarly affected mother. A 1.4-Mb inversion on 10q23 was identified in both affected individuals (Figure S20), for which the proximal breakpoint lies in intron 1 of *BMPR1A* (GenBank: NM_004329.3). As the first exon is 5′ UTR, we intend to perform follow-up studies using informative exonic SNPs (rs35572415 and rs7078571) for allelic imbalance experiments to confirm the effect of this SV on transcription. The inversion has been validated using a triple-primer multiplex PCR assay, and thus cascade testing can now be offered to at-risk family members.

Family 2 comprised a proband and her daughter both recruited to the 100kGP due to cutaneous and uterine leiomyomas. Although no renal cancer had been reported in this family, there was a strong suspicion of an underlying germline variant in *FH*. Prior testing with targeted sequencing and MLPA did not uncover any likely diagnostic variants. We detected a complex SV with a breakpoint in the in the final intron of *FH* (Figure S21A), consistent with leiomyomatosis and renal cell cancer (MIM: 150800). Although our prioritization was due to the Manta algorithm, which called this SV as a 3.6-Mb inversion, the actual structure was an intrachromosomal translocation of a 193-kb segment, in combination with a 188-kb deletion (Figures S21B and S21C). The consequence of the insertion on the *FH* transcript would require RNA studies. However, the inserted sequence contains one positive-strand RefSeq annotation for "POTE ankyrin domain family, member F pseudogene" (GenBank: NR_027247.2), and so we speculate that the SV may result in a fusion transcript. As the variant was also detected in the proband's affected daughter, there is support from co-segregation. Cascade testing is now being extended to include other affected family members. We anticipate that this finding will be important for prioritizing renal cancer surveillance, especially for male relatives where the endometrial phenotype cannot act as a "giveaway" clue about whether individuals are likely carriers and thus at risk of the more aggressive cancer type.

Family 3 comprised an individual entered into the 100kGP as a singleton with a diagnosis of "genodermatoses with malignancies." Other features included punctate palmoplantar hyperkeratosis, thyroid adenoma, macrocephaly, and tongue nodules. Although the phenotype was considered classic for Cowden syndrome (MIM: 158350), previous testing of *PTEN* using targeted sequencing and MLPA approaches had not picked up any variants of significance. We identified a balanced 14-kb inversion involving exons 6–8 of *PTEN* (Figure S22) (GenBank: NM_000314.8). As above, it is anticipated that this finding will facilitate cascade testing for other at-risk family members.

Family 8 comprised an individual with multiple tumors recruited to the 100kGP as a singleton. She was diagnosed with unilateral retinoblastoma (MIM: 180200) in 1971 and subsequently osteogenic sarcoma of the left tibia in 1981. Malignant melanoma and uterine leiomyosarcoma were also reported more recently. Although the pedigree was not available to review, extensive family history of osteosarcoma, retinoblastoma, and breast cancer was noted. Several malignancies were reported to have somatic mutations in *RB1* (MIM: 614041), but targeted sequencing of *RB1* in constitutional DNA did not pick up any pathogenic variants. Our analysis identified an 18.1-kb inversion, with the distal breakpoint lying in intron 2 of *RB1* (GenBank: NM_000321.3), and a 2,246-bp loss at the proximal end (Figure S23). Unfortunately, the individual is now deceased, which makes it difficult to re-contact this family to offer validation/cascade testing.

Family 40 comprised a male recruited as singleton to 100kGP under a diagnosis of familial tumor syndromes of the central and peripheral nervous system. SVRare had prioritized a 15.1-Mb inversion call that involved *NF2*. Closer scrutiny of the MantaINV call and read alignments around this locus indicated that the SV comprised three interlinked duplications of 19.5 kb, 10.1 kb, and 1,329 bp in size (Figure S24). Although linkage analysis had already revealed a risk haplotype for markers around *NF2*, detection of this SV will facilitate cascade testing in other at-risk family members. This rearrangement had already been uncovered independently by the clinical team responsible for this individual, following discussions at an MDT meeting, and has been described in more detail elsewhere.[45]

As described above, Family 4 harbored an inversion that disrupts *EXT2*, likely resulting in multiple bone exostoses described in three family members. Although multiple bone exostoses (MIM: 133701) are typically benign and often asymptomatic, in some affected individuals they can result in pain/deformities and can be surgically removed. One important complication is the increased risk of malignant transformation to a secondary chondrosarcoma,[46] and so our findings could help inform surveillance. One such (unrelated) participant with a germline deletion-inversion in *EXT2* from the cancer arm of the 100kGP is shown in Figure S25.

The last three families with inversions disrupting tumor suppressor genes include a founder inversion involving exons 2–6 of *MSH2* in two apparently unrelated individuals (Families 16 and 17) and the complex translocation disrupting *APC* (Family 43). These are described in more detail below.

Selection of illustrative exemplars

Exemplar 1: An intragenic MSH2 *founder inversion*

We identified two apparently unrelated individuals from the 100kGP harboring a 19.1-kb inversion that involves exons 2–6 of *MSH2*, with a 1.2-kb loss at the distal end (Figure 3A). The first individual (Family 16) is a male recruited to the 100kGP with reported transitional cell cancer of bladder at 42 years and cecal cancer at 45 years. Positive family history of early-onset cancer stretched back at least three generations (Figure 3B) and led to a strong clinical suspicion for Lynch syndrome. Microsatellite instability was demonstrated in DNA from cecal tumor (5/5 markers). Immunohistochemistry highlighted a complete loss of MSH2 expression and reduced MSH6 expression in both bladder TCC and cecal tumors. Germline testing using both targeted sequencing and MLPA approaches was unrevealing. Karyotyping (46XY) and array-CGH had also returned normal results. There was no evidence for loss of heterozygosity at any of the *MSH2*, *MLH1*, or *MSH6* loci. However, somatic testing identified a single *MSH2* variant in 20% of reads. This c.508C>T (GenBank: NM_000251.3) transition predicts a nonsense allele, p.Gln170*, and has been reported multiple times in ClinVar (VCV000091117.20). In summary, despite the strong clinical suspicion for a germline variant in *MSH2*, i.e., Lynch syndrome 1 (MIM: 120435), both prior testing and the initial report from the 100kGP had not identified any variants of significance.

The second 100kGP participant who carried the same inversion (Family 17) was a female with leg sarcoma at 44 years, endometrial cancer at 55 years, and renal pelvis cancer at 60 years. Bowel cancer was also reported in her now-deceased father and brother (Figure 3B), and so the family background was again considered to be classic for Lynch syndrome. Given the complexity of the rearrangement and the identical breakpoints shared between both individuals, we speculated that the variant was a founder variant. Although haplotype analysis was limited by the fact that genome sequencing in each family was performed on just the proband, a 3.2-Mb region where there is absence of conflicting homozygosity (Figure 3C) was identified, supporting a founder origin. The maximal coordinates of this shared region are defined by A>C transversions at rs115321698 and rs13420048, where the alternate allele was homozygous in Family 16 but not detected in Family 17 (Figure S26). Analysis of ultra-rare variants further supported the founder origin hypothesis, and the 13 shared SNVs identified (Table S4) may be useful to screen for other individuals carrying this inversion. Assuming a "correlated" genealogy, we estimate that the mutation arose 29.9 generations ago (95% CI: 9–112.7). Neither the founder *MSH2* inversion nor any of the inherited SVs reported here were detected in the gnomAD SV v.4 database.

Exemplar 2: Complex MECP2 *rearrangements resolved by PacBio sequencing*

Rett syndrome (MIM: 312750) is a neurodevelopmental disorder caused by *MECP2* variants that mainly affects females. After a period of normal or slow development (7–18 months), individuals show arrested development, regression of acquired skills, and loss of speech. Ataxia, stereotypic movements (e.g., distinctive uncontrolled hand clapping/rubbing), acquired microcephaly, seizures, intellectual disability and autistic-like behaviors are often

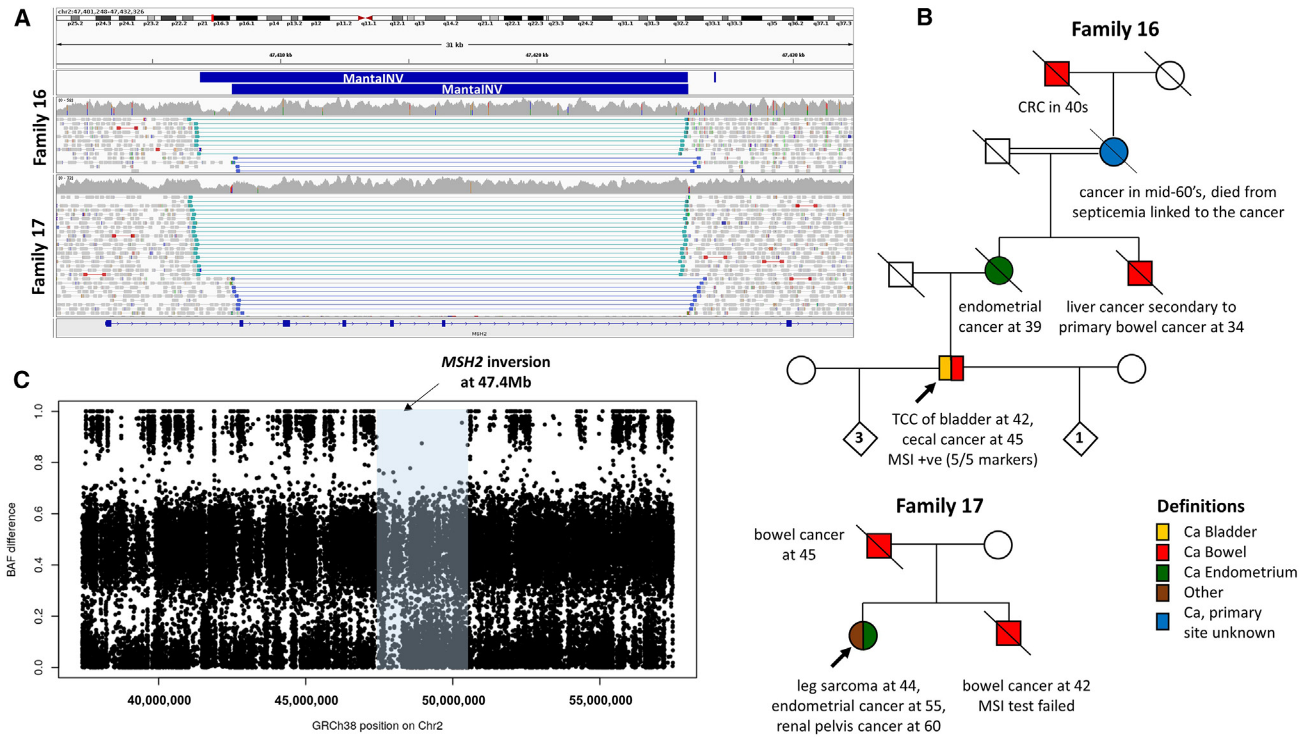

Figure 3. Identification and haplotype analysis of founder *MSH2* inversion
(A) IGV screenshot showing read alignments supporting inversion of *MSH2* exons 2–6 in Families 16 (upper) and 17 (lower), viewed using the "view as pairs" and "collapsed" options. Reads are sorted by insert size. Coordinates for two MantaINV calls (blue) are chr2:47,406,871–47,425,914 and chr2:47,408,111–47,425,934 (GRCh38). A drop in coverage at the distal end reflects a 1.2-kb deletion, which was not called by Canvas. Transcript shown is GenBank: NM_00251.3.
(B) Pedigree and clinical information for Families 16 and 17. Symbol shading is only for cancer onset under the age of 70. Cascade testing was not possible for deceased individuals.
(C) Conflicting homozygosity analysis for high-confidence SNVs shows evidence for a shared ~3-Mb haplotype (blue shading) surrounding the *MSH2* locus. The region shown corresponds to the *MSH2* locus, with 10 Mb added at each end (chr2:37,401,067–57,485,228).

reported. The same variants that cause Rett syndrome in females are considered lethal or else result in a much more severe presentation in males. Classically affected males have been described harboring mosaic variants or in individuals with an extra X chromosome (i.e., Klinefelter syndrome, 47,XXY). In this study, two 100kGP participants with MantaINV calls were identified in which small inverted segments were part of more complex rearrangements, and both involved the final coding exon of *MECP2*.

For Family 33, the male proband was recruited to the 100kGP with a diagnosis of intellectual disability. Other features included microcephaly, seizures, delayed motor development, and ataxia. Autistic behavior, recurrent hand flapping, and inappropriate laughter were also noted. Although Manta detected two overlapping inversions, closer scrutiny of the locus suggested a complex maternally inherited SV involving a 1,130-bp deletion and a 681-bp duplication (Figure 4A). Due to the size of the duplication, which could not be spanned using short paired-read data, the configuration of this SV was ambiguous. Using HiFi long-read genome sequencing data, we showed that the correct configuration was the one that led to the later truncation, p.Leu336Profs*18. A dot plot showing a representative 22-kb read is shown in Figure 4B. A similar dot plot (using a hypothetical sequence) shows the alternative structure that would also have been a potential solution to the short-read data (Figure 4C), and this would have led to much earlier truncation of *MECP2* at codon 137, within the methyl-CpG-binding domain (MBD).

In contrast, Family 47 comprises a female who underwent an initial period of normal development followed by regression, especially of speech, from age 3 onwards. There was significant global developmental delay and microcephaly. She demonstrated periods of fast irregular breathing, and there were no purposeful hand movements. At the time of last review, age 8, she was able to walk a few steps aided. She had no meaningful words and communicates through picture exchange. She developed multi-focal seizures from 5 years of age. Array testing in 2013 (80 × 60K v.2.0, ISCA platform) had identified a maternally inherited microduplication at 7q36 considered not to be significant. Sequence analysis and MLPA of *MECP2* a year later identified no pathogenic variants. In 2015, a differential diagnosis of Angelman syndrome was pursued, which included testing with methylation-specific PCR methods. However, there was no evidence of a deletion, uniparental disomy, or an imprinting defect at the *UBE3A* locus. The family was then recruited to the 100kGP as a parent-child trio,

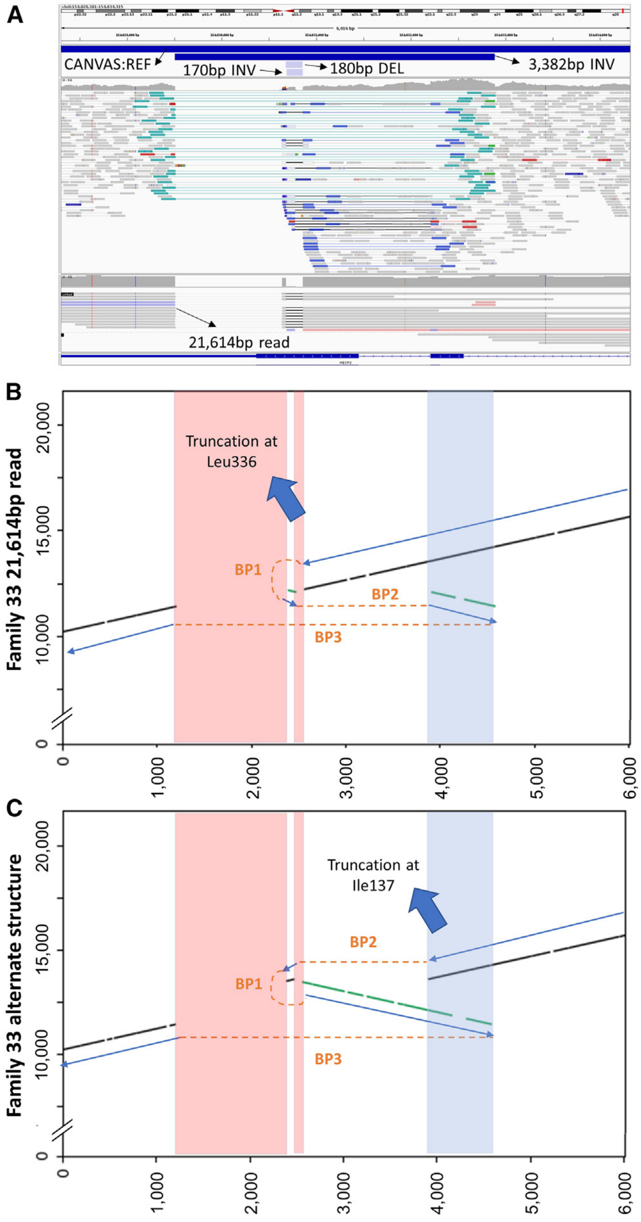

Figure 4. Complex rearrangement involving *MECP2* solved by long-read sequencing

(A) Read alignments from short-read (150-bp pared-end, upper) and long-read (PacBio, lower) analysis supporting complex DEL-INV-DUP involving *MECP2* in Family 33. Reads are shown in IGV using the collapsed setting. Illumina data are shown using the "view as pairs" option, while PacBio reads are shown using the "link supplementary alignments" option. The SV was called by Manta as a deletion and two overlapping inversions but was missed by Canvas. The transcript shown is GenBank: NM_001110792.2.

(B) Dot plot constructed using a single representative positive-strand PacBio read of 21,614-bp shown in (A), compared to the GRCh38 reference. Red shading represents deleted regions; blue shading indicates a duplicated region.

(C) Dot plot (as above) showing a hypothetical rearrangement that highlights the alternative structure that would have been possible from the short-read data alone. The x axis in all panels corresponds to chrX:154,028,301–154,034,315 (GRCh38). Gray and green lines indicate sense/antisense matches to the reference; the blue arrows (sequence present) and orange lines (junctions) help explain how these segments are connected. BP, breakpoint.

and genome sequencing was performed in 2017. The 100kGP clinical pipeline picked up a maternally inherited c.1210G>A (p.Glu404Lys) (GenBank: NM_001099922.3) in *ALG13* (MIM: 300776) of uncertain significance (VCV000982621.4), for which further co-segregation/glycosylation studies had been considered. Although the MantaINV call in *MECP2* prioritized by SVRare suggested an inversion of 209 bp, close scrutiny of the locus with the short- and long-read data available suggested a complex rearrangement comprising a 76-bp inverted duplication close to a similar-sized deletion. However, MantaBND calls also showed the presence of a 14.5-kb duplicated segment from 19qter, which had inserted into the middle of the SV (Figures S27A and S7B). Similar to the situation for Family 33, this complex SV was ambiguous with short-read genome sequencing data alone (Figure S27C), as the rearrangement could also be a result of a translocation (i.e., two derivative chromosomes with a duplication at the site of the breakpoint). The presence of two PacBio reads of >20 kb spanning the 14.5-kb duplicated region (Figures S27A and S28) confirmed the structure to be an inter-chromosomal duplication and not a translocation. This finding helps confirm the disruption to *MECP2* and alters the clinical interpretation of this variant.

Exemplar 3: Resolving a long diagnostic odyssey—Regulatory inversion in the HOXD *cluster*

Family 42 were recruited to the 100kGP due to mesomelic limb shortening, most pronounced in the upper limbs. Radiological findings included severe shortening of radius/ulna, with bowing of the radius and dislocation of the radial head. Detailed clinical information for this family was reported in 2004, and at that time the authors suggested this to be only the second family ever described with mesomelic dysplasia, Kantaputra type (MIM: 156232).[47] The molecular basis for the *ulnaless* mouse model was shown in 2003 to be a 770-kb inversion of the *HOXD* gene cluster.[48] These mice show striking mesomelic shortening, especially in the forelimbs, and this prompted Sanger sequencing of *HOXD11* (MIM: 142986) in Family 42, which (along with karyotype testing) had not detected any variants of significance.[47] In addition, 244k Agilent array-CGH had not detected any gross structural changes.[49] We identified a 22.9-kb inversion involving the *HOXD* gene cluster (*HOXD11-13*) that had arisen *de novo* in the proband and had been transmitted to the proband's similarly affected son (Figure 5A). It is notable that twice in the literature it has been suggested that this family may harbor a regulatory SV involving the *HOXD* cluster,[47,49] but at those times, genome sequencing technologies were not available. Even after the genome sequencing data became available in 2018, it took another 3 years before this inversion was uncovered (Figure 5B). In addition to the resolution of a long diagnostic odyssey, this finding could be of direct clinical utility for the son in terms of family planning.

In contrast to Family 42, the original family with mesomelic dysplasia, Kantaputra type, first described in 1992,[50]

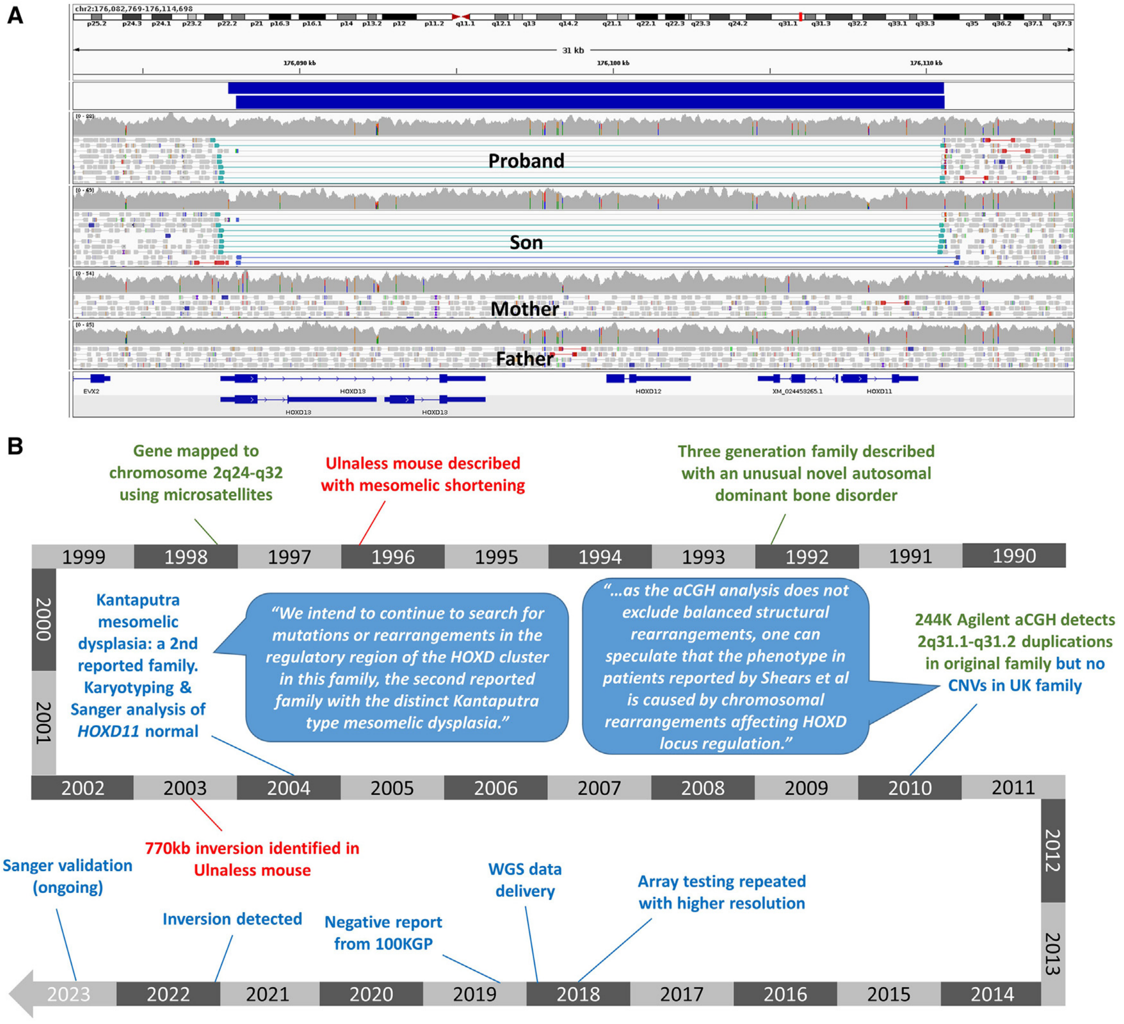

Figure 5. A *de novo* inversion of the *HOXD* cluster linked to a historical description of mesomelic dysplasia, Kantaputra type
(A) Read alignments supporting an inversion of *HOXD* gene cluster present in the proband and her son but not in the proband's parents. Coordinates for two MantaINV calls (blue) are chr2:176,087,987–176,110,607 and chr2:176,087,748–176,110,599. Although the rearrangement does not disrupt the MANE transcript for *HOXD13* (ENST00000392539.4/GenBank: NM_000523.4), the other annotated transcripts displayed (GenBank: XM_011511069.2 and GenBank: XM_011511068.2) are disrupted. The inversion overlaps one of the duplicated segments identified in the original family; see https://genome.ucsc.edu/s/AlistairP/HOXD_cluster_SVs.
(B) Timelines relating to Family 42 (blue) and the original family (green) are shown alongside relevant mouse studies (red). Speech bubbles show quotes from Shears et al., 2004 and Kantaputra et al., 2010.[47,49].

was a much larger kindred, and linkage to 2q24-32 was demonstrated in 1998.[51] In 2010, two rare duplications involving 2q31.1-q31.2 and encompassing approximately 481 kb and 507 kb were identified. Without genome sequencing data or another method for breakpoint characterization, it is impossible to assess whether these two duplications are independent direct-tandem repeats or whether the two segments are interlinked. Nevertheless, we note that the 22.9-kb inversion described here overlaps the smaller of the two duplications in the earlier family.

Although the proximal breakpoint of the 22.9-kb inversion disrupts the 5′ UTR of non-canonical *HOXD13* transcripts (GenBank: XM_011511068.2, GenBank: XM_011511069.2), the canonical transcript (GenBank: NM_000523.4) remains intact, as does *HOXD11* (GenBank: NM_021192.3) and *HOXD12* (MIM: 142988; GenBank: NM_021193.4). We therefore hypothesize that the biological mechanism leading to the mesomelia dysplasia is due to a positional effect. The new chromosome structure would mean that *HOXD13* (MIM: 142989) is under the control of more proximal promoters, i.e., those that normally control *HOXD11* and *HOXD10* (MIM: 142984). This could result in the aberrant expression of *HOXD13*. It has been shown in the chick[52] and other models that mis-expression of *HOXD13* orthologs can result in shortening of the long bones.

Exemplar 4: A complex translocation disrupting APC *helps resolve a clinical conundrum*

The proband in Family 43 (I-1; Figure 6A) first presented with tiredness and shortness of breath aged 25 and lost considerable weight over the next 5 years. He had stomach cramps and was diagnosed later with hundreds of polyps

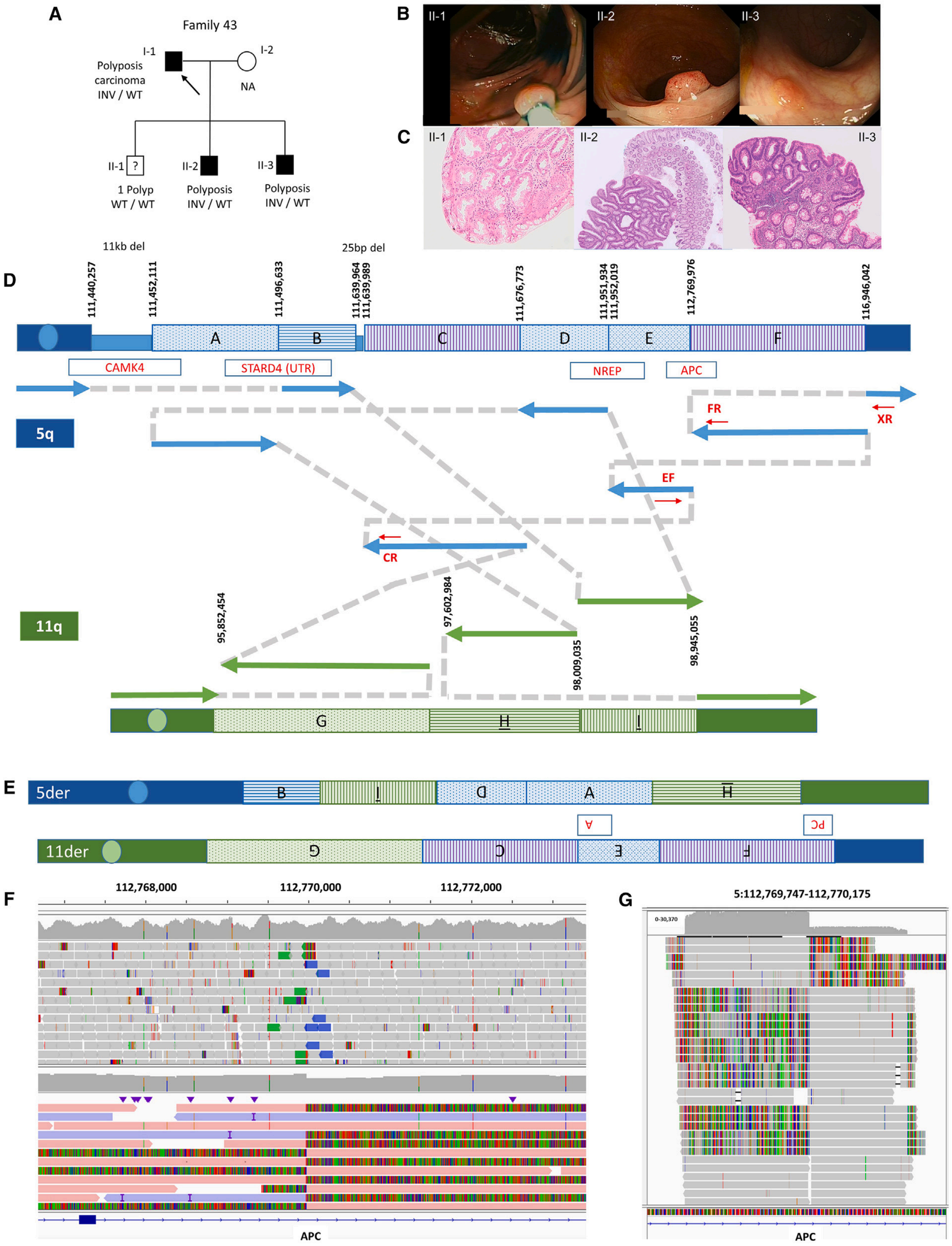

Figure 6. Clinical and genetic characteristics of Family 43 with a complex translocation involving the *APC* locus

(A) Pedigree including proband and the three male offspring, of whom two share the complex translocation (INV). NA, not tested; WT, wild type.

(B) Endoscopy images showing polyps in all three siblings, II-1, II-2, and II-3. For individual II-1, endoscopy detected just a single sessile serrated polyp, and so affection status was clinically uncertain. For II-2 and II-3, a single representative polyp is shown.

(legend continued on next page)

and colorectal cancer, requiring colectomy. This presentation was thought to be in alignment with familial adenomatous polyposis syndrome (FAP [MIM: 175100]). This was investigated using MLPA and an 18-gene bowel panel test, but no likely-pathogenic variants were found. Although the proband's oldest son (II-1) was asymptomatic, at age 15 years an endoscopy was performed to exclude any polyposis similar to that seen in his father. At the hepatic flexture, a single sessile serrated polyp was detected. No other polyps were identified, and so the significance of this single polyp was unclear. The second-oldest son (II-2) was diagnosed with type 1 diabetes mellitus (MIM: 222100) aged 3 years and autism spectrum disorder aged 11. At 13, he experienced intermittent bouts of diarrhea (no blood and no mucus) and sometimes reported stomach pain. Gastroscopy and colonoscopy were performed to investigate a polyposis syndrome as well as celiac disease for the diarrhea. The colonoscopy showed multiple small polyps. The microscopic pictures show some lymphoid tissue from the Peyer's patch in the terminal ileum, as well as several large colon mucosa focal tubular adenomas, and low-grade dysplasia identified in several biopsies. Over 100 adenomas were identified aged 15 years (including focal tubular adenoma). Follow-up endoscopies confirmed polyposis and tubular adenoma and low-grade dysplasia. A number of investigations to identify a genetic cause (in particular *APC* variants) were unrevealing. The youngest son (II-3) also underwent a colonoscopy aged 10, and >100 adenomas were identified, confirming polyposis with histology tubular adenoma and low-grade dysplasia.

In summary, the family pedigree (Figure 6A) strongly suggested a dominant genetic cause, and family history was consistent with a clinical diagnosis of FAP. The endoscopic (Figure 6B) and histological features (Figure 6C) were also consistent with FAP, but multiple genetic investigations, including the targeted testing described above as well as the initial analysis of genome sequencing data from the 100kGP, did not reveal a genetic cause. A further complication was the fact that the oldest son had just a single polyp, and so it was unclear whether this was a chance finding or represented delayed progression of a genetic polyposis syndrome. Given this uncertainty, this elder brother had not been recruited to the 100kGP.

Re-analysis of genome sequencing data using SVRare revealed a 4.2-Mb MantaINV call with a breakpoint in intron 4 of *APC* that was predicted to disrupt gene function. However, manual assessment of read-level data detected additional split-read pairs mapping to multiple junction sites (Figure 6D). Overall, the pattern of nine rearranged genomic segments was suggestive of a complex chromosomal translocation (Figure 6E). A translocation involving chromosomes 5 and 7 was subsequently confirmed by FISH. Long-read genome sequencing using PacBio HiFi reads helped validate all respective breakpoints (Figure 6F). Finally, PCR analysis using two sets of primers, followed by nanopore sequencing, confirmed the two clinically relevant breakpoint junctions (Figure 6G).

RNA-seq data available for the proband (II-2) indicated *APC* to have a 0.65-fold change in expression (adjusted p value 1.51×10^{-10}). Other genes disrupted or lying close to breakpoints were also downregulated (Table 1). The presence of a common six-SNP haplotype (rs2229992-rs351771-rs41115-rs42427-rs866006-rs465899) in the coding region of *APC* allowed us to assess allelic imbalance. RNA-seq for the proband (II-2) indicated that only the non-reference alleles were expressed (Figure S16). Phasing was not possible by inheritance, as the haplotype was heterozygous in all genome-sequenced family members. However, PacBio data (available for I-1) confirmed that this non-reference SNP haplotype involved the non-rearranged copy of *APC*, i.e., with the complex translocation lying in *trans*.

The finding of a complex translocation, now validated using multiple methods, allowed us to make a definitive genetic diagnosis in the three affected individuals from Family 43. Furthermore, the diagnosis facilitated accurate genetic counseling of the family and the initiation of screening, which then excluded this genetic polyposis condition in the family member (II-1) who had presented with a single polyp. This finding will inform clinical management in terms of the regularity/necessity for ongoing colorectal cancer surveillance.

Discussion

In this study, we utilized data from 33,924 families in the RD program of the 100kGP with the aim of investigating the role of inversions in genetic disease via HI. Our study

(C) Histological images showing H&E staining of a solitary polyp without dysplastic changes in II-1 and an example of APC-like polyps II-2 and II-3.
(D) Subway plot showing the complex structure of the translocation. The rearrangement involves nine segments and is largely balanced, with the exception of 11-kb and 25-bp deletions. Breakpoint positions on chromosomes 5 and 11 are labeled using hg19 coordinates (GRCh38 coordinates are in Table S2). Segment sizes are not to scale. Segment "F" was called as a 4.18-Mb inversion by Manta, which is how the SV was first identified. Approximate positions of PCR primers used to validate the clinically relevant breakpoints BP1 (EF-CR) and BP2 (XR-FR) are shown by red arrows. Genes disrupted by breakpoints are highlighted.
(E) Schematic diagram of the derivative chromosome structures. The position of the *APC* disruption is indicated.
(F) Comparison of Illumina and PacBio read alignments shown using IGV and the "show soft-clipped reads" option. The breakpoint in intron 4 of *APC* (GenBank: NM_000038.6) is indicated.
(G) Read alignments from nanopore sequencing of PCR products using two junction-specific primers and DNA from individual II-3. Sequence was generated for both breakpoint 1 (406 bp) and breakpoint 2 (361 bp), and reads were merged into a single BAM file. Results were consistent with Illumina/PacBio data.

also included complex SVs that were detected due to an inversion call. Although 62 individuals from 47 families were nominated for inclusion into the diagnostic discovery pathway for follow-up, two were subsequently refuted, and so the final yield was 45/33,924 families. This represents only 1%–2% of the total diagnoses across the 351 genes assessed. A notable finding was that for the vast majority (87%) of SVs, the inversion was below 10 Mb in size and thus would be too small to be detected by karyotyping (Figure 1A). We also found that 20/47 of the SVs reported here were confirmed to have arisen *de novo*. This was not a requirement for the initial SV prioritization steps (which only needed an allele count of 5 or lower), so this is strong evidence supporting pathogenicity for this collection of variants.

Our reported incidence (~1/750 families) likely represents an underestimate of the overall impact of inversions in RD for several reasons. Firstly, our analysis was limited to inversions that disrupted known HI genes. Our list of 351 genes (Table S1) represents <10% of the 4,836 genes known to harbor phenotype-causing mutations listed in OMIM (August 31, 2023) and was taken from the Clinical Genome Resource, whose curations are conservative and do not include disease-gene associations described recently. For instance, *SRRM2* (MIM: 606032) was not included in this list despite good evidence for a role in intellectual disability via HI,[53,54] and we note that three participants with deletion-inversions (likely palindrome-mediated) involving this gene were identified recently in the 100kGP.[55]

Secondly, this study did not scrutinize SVs that disrupt genes linked to autosomal recessive conditions. Two such examples from the 100kGP are described in Note S5. Performing a systematic analysis of inversions across the entire 100kGP for genes linked to recessive conditions is confounded by several factors: (1) unless the SV is homozygous, one has to identify a second hit variant and assess pathogenicity for both variants in tandem; (2) even if one finds a strong second hit, not all families from 100kGP are parent-child trios, so with limited access to long-read sequencing data, phasing information is incomplete; and (3) allele frequency filtering would need a less stringent cut-off. In the future, long-read genome sequencing will largely solve the problem of phasing rare variants and may also help identify additional SVs where breakpoints lie in repetitive regions. The 150-bp reads used here could potentially have missed inversions where the breakpoints lie in Alu/LINE1 elements, pseudogenes, or any region where there is high sequence homology.

Several genes were recurrently impacted by inversions in this cohort. Families 37 and 38 both harbored inversions disrupting *AUTS2* (GenBank: NM_015570.4). The respective breakpoints in intron 2 and 5 are not nearby, and so there is no indication of a mutational hotspot (Figure S29). *AUTS2* has a large genomic footprint of 1.2 Mb and so simply represents a greater mutational target for rearrangements to occur. Several structural rearrangements in this gene linked to intellectual developmental disorder, autosomal dominant 26 (MIM: 615834), have been reported previously.[15,56]

In contrast, the recurrent disruption of *MSH2* was due to the same founder variant being detected in two independent families from the UK. An identical *MSH2* rearrangement was identified in an Australian proband by cDNA sequencing and inversion PCR in 2016, with an AluY-mediated recombination model being hypothesized to explain its origin.[57] The same primers used in that study were used for validation for the individuals identified here. A second proband was subsequently detected as part of a replication effort that tested 55 individuals with unsolved Lynch syndrome,[57] and that individual was re-reported by Brennan et al.[58] The 3.2-Mb haplotype shared between Family 16 and Family 17 described here (Figure 3C; Table S4) represents formal confirmation of a founder origin. Recent genome sequencing of the second proband described by Liu et al.[58] has confirmed that the inversion in that individual lies on the same founder haplotype (Table S4). This exon 2–6 *MSH2* inversion was estimated to have arisen around 30 generations ago. As a rule of thumb, shared segments of 2–5 Mb are most likely inherited from a common ancestor >20 generations ago,[59] but this depends on local recombination rates. The two families reported here and the second individual reported by Liu et al.[57] all have recent ancestors from the same region in northern England. Further genealogy studies linking the two UK families with both Australian families or else performing additional analyses of identity-by-descent would give a more precise estimate. Given the founder origin and cryptic nature of this rearrangement, it is important that the prevalence of this inversion is assessed in other suitable populations where (1) Lynch syndrome is suspected and (2) loss of MSH2 has been confirmed at the protein level. In one follow-up study, Morak et al. screened 48 MSH2-deficient affected individuals from a German population but did not find this exon 2–6 inversion,[60] suggesting this founder variant to be of more westerly European origin. MRC Holland are investigating whether probes for this inversion can be incorporated into one of their probe-mixes in the future (J. van der Meer, personal communication). A larger overlapping 10-Mb inversion[61] involving *MSH2* exons 1–7 explained 60% of affected individuals in a cohort of suspected cryptic Lynch syndrome,[62] and consequently that inversion is now being captured by MLPA testing (Note S1).

The proband in Family 33, where a complex deletion and duplicated inversion was inherited from an unaffected mother, represents atypical (male) Rett syndrome. In contrast, the second *MECP2* family comprised an affected female, where classical Rett syndrome had long been suspected. In the second participant (Family 47), we found that a duplicated segment from 19qter had inserted into *MECP2*. Both SVs occurred at a similar position within the last exon of *MECP2* (Figures 4A and S27), a known mutational hotspot for complex SVs.[63] The structural

ambiguities for both of these SVs from the short-read data could be resolved with long-read PacBio data (Figures 4B and S28), and determining the precise nature of these rearrangements influenced the respective clinical interpretations. The major functional domain encoded by *MECP2* is the MBD, which involves residues 102–174 (GenBank: NM_001110792.2; or 90–162 on UniProt: P51608). To date, the reported truncating variants in affected males (non-mosaic) always lie after this domain. The earliest non-mosaic truncation to be described in a male individual that we are aware of is c.524_525del (p.Gly175Glufs*11) (GenBank: NM_001110792.2), and that was associated with a very severe presentation, involving neonatal encephalopathy and bilateral polymicrogyria, and the individual died at 13 months.[64,65] For Family 33, the alternative SV configuration would have led to an earlier truncation at codon 137 (Figure 4C) and hence would be expected to result in lethality for males. So a genuine germline truncation at p.Ile137 seems unlikely and may have led to a suspicion of mosaicism. In Family 47, the presence of a classical translocation would have altered how the family was counseled and determined which methods would be viable options for validation/cascade testing. Together with a similar case report,[66] our work poses the question of whether other unsolved individuals with Rett syndrome might be explained by cryptic SVs involving *MECP2*.

Although the present study only aimed to identify inversions that disrupt the coding sequence of genes known to cause disease through HI, large SVs can also alter chromatin structure, which in turn can lead to changes in gene expression. A good example of this phenomenon from the 100kGP includes several families with complex duplication-inversions on chromosome 17 that create new topological-associated domains and result in a dominant form of retinitis pigmentosa.[67] The bioinformatic filtering steps performed in our study meant that for most participants, the inversion breakpoints lay between coding exons. For this class of balanced inversion, the ACMG criteria PVS1[68] can typically be applied. However, there were notable exceptions to this rule. For the individual with craniosynostosis 1 (MIM: 123100) and an inversion involving *TWIST1* (Family 24), the closest breakpoint lay 18 kb downstream of the disease-relevant gene, and so a position effect needs to be invoked. Pathogenicity was supported from a combination of co-segregation together with clinical specificity.[32] In the present study, this inversion was identified due to a longer overlapping transcript isoform (ENST00000443687.5) in the ENSEMBL v.105 annotation set. Similarly, the proximal breakpoint for the inversion involving *HOXD11-13* (Family 42) was exonic only for non-canonical *HOXD13* isoforms. This variant was supported by *de novo* occurrence, onward transmission to an affected son, and a high degree of clinical specificity. Several studies have investigated the chromatin domain boundaries at the *HOXD* locus. Detailed characterization of CTCF sites suggests this locus is divided into two, a proximal domain and a distal domain. Division between both likely occurs between *HOXD13* and *HOXD11*.[69] The inversion described here would turn the CTCF sites around and would likely have an impact on gene expression during critical stages of limb development. A third exception was the complex SV in Family 44 involving interlinked duplications on 11p15.4. *CDKN1C* is a well-known locus for Beckwith-Wiedemann syndrome-associated rearrangements—the first balanced rearrangements clustering 100-kb from *CDKN1C* were described nearly 30 years ago.[70] Our hypothesis is that this SV disrupts the maternal methylation status of the IC2 region, upregulating expression of *KCNQ1OT1* (MIM: 604115; KCNQ1 opposite strand/antisense transcript 1), and consequential repression of *CDKN1C*. Although no epigenetic data were available for this individual, studies have shown that methylation signatures can be critical for helping confirm or propose new diagnoses for a range of genetic conditions.[71] In general, the effect of complex SVs on methylation status is poorly understood. However, a recent study used array and nanopore technologies to confirm that a DUP-TRP/INV-DUP on chromosome 14 resulted in a methylation pattern consistent with UPD(14)mat (Temple syndrome, MIM: 616222).[72] For our last two families (Families 42 and 44), the target genes are small, and so there were far fewer SVs listed in the SVRare report. In combination with the highly specific phenotypes, these SVs were able to be detected prior to the final filtering step, in which unequivocal gene disruption was a requirement. These highlight the importance of future work toward prioritizing SVs with position effects in a more systematic fashion.

The balanced rearrangement involving *APC* (Family 43, Figure 6A) resolved a long-standing clinical conundrum and allowed us to determine that the single polyp seen in the elder brother was simply a chance finding. In addition to impacting disease surveillance decisions, the finding may be useful in future for family planning. We note that a recent study describes a largely balanced chromothripsis event involving the *APC* locus as a germline cause of a colon cancer predisposition.[73] Although that rearrangement involved the translocation of ten fragments from 5q22.1q22.3 into 10q21.3 and showed a level of complexity similar to the rearrangement seen here, it was not a translocation in the classical sense and so would not have been identified by karyotyping. Other examples of cryptic *APC* variants are provided in Note S6. Our results therefore strengthen the rationale that individuals with a strong clinical suspicion for a germline *APC* variant that remain unsolved following standard testing approaches should be considered for genome sequencing to uncover potential cryptic variants. The complex translocation identified here poses the related question of how often translocations uncovered by karyotyping have this degree of complexity. An earlier study found that 26% of balanced SVs detected by karyotyping involved three or more breakpoints.[15]

For 8/47 families, we are still not in touch with the recruiting clinician, despite repeated attempts to make contact over an ∼18 month period. Similar difficulties were also noted in a recent commentary, where a response rate of 20% was noted.[74] The reasons for this are often due to the time gap between recruitment and the research finding being uncovered (>5 years for several participants). In that time an appreciable turnover of clinicians is to be expected. At the time of writing, 60% of these SVs reported here have been confirmed by an orthogonal approach. The validation results summarized in Table S2 are intended to give a snapshot of an ongoing process. Our experience highlights variable levels of expertise and resources across different GLHs to perform validation, with many clinical laboratories having significant backlogs for validation of 100kGP findings considered non-urgent. Overall, validation efforts were split across research and clinical settings and involved a range of approaches. Once a complex SV is validated, interpretation also comes with difficulties. Although the ACMG/AMP variant classification guidelines have been adapted for single-gene CNVs,[75] clinical reporting guidelines currently do not cover complex SVs in detail.

Although exome sequencing can detect SVs in a small fraction of affected individuals,[76] the non-uniform coverage makes this approach non-optimal. For 100kGP participants who had previously been entered into the DDD study, retrospective analysis was only able to confirm SV breakpoints for 2/6 of these. We note that for the *SOX5* rearrangement (Family 6), exome data only captured the breakpoint for 1/6 reads, and so the SV would not have been called robustly without prior knowledge. Although the inversion disrupting *DYRK1A* (Family 36) was not captured by exome data due to breakpoints lying in intronic regions, a different inversion disrupting the same gene was identified in another individual from the DDD study as both breakpoints happened to lie in exonic regions.[77]

For one of the two families with SVs involving *ARID2*, the SV was interpreted as an inversion at the time of initial review but was later considered more likely to be an integration of an S group subfamily Alu element. The pattern of read alignments was very similar to that seen for a putative inversion involving *CASK* (MIM: 300172), from a recent study using genome sequencing on 465 families with neurodevelopmental short-read data.[78] In that study, the SV could not be confirmed by long-range PCR or by long-read genome sequencing, suggesting it to be a false positive. Other examples of complex SVs with incomplete interpretation are provided in Note S7. In combination, these reports highlight that even with full genome sequencing data, careful scrutiny of read alignments is critical. Together with the initially ambiguous SVs involving *MECP2* described in Families 33 and 47, these data highlight that, where duplicated segments are involved, genome analysts should always consider whether other potential SV configurations could explain the short-read data. In situations where gene disruption (i.e., PVS1) cannot conclusively be inferred, additional testing is often needed to achieve diagnostic levels of certainty. The 681-bp duplicated segment found in Family 33 was not spanned by Illumina read pairs, and so clinical interpretation was uncertain until PacBio data were obtained (Figure 4B). In contrast, a similar complex SV involving a 406-bp duplication in *SCN5A* (MIM: 600163), found in a participant from the 100kGP pilot study with suspected Brugada syndrome and who had remained unsolved from initial analysis,[6] was resolved (Figure S30). The threshold for resolving duplicated segments in complex SVs using current Illumina 150-bp genome sequencing data is likely around 500 bp. In contrast, we showed that for a proportion of individuals, the ability of long-read genome sequencing to span larger segments can help resolve complex SVs. The duplicated segment of 14.5 kb in Family 47 was able to be spanned using PacBio HiFi data, and this influenced how the SV was interpreted clinically. Due to limitations of current HiFi technology, segments of >20 kb would be difficult to resolve, and other methods may need to be employed. We recently resolved a complex SV using a combination of simple RNA methods and Bionano optical genome mapping, the latter in which a high fraction of molecules >500 kb were obtained.[79]

Although the majority of variants described here were identified via bioinformatics prioritization steps, Families 23 and 40 were initially identified as a consequence of MDT review meetings, which led to manual scrutiny of read alignment data. In clinical laboratories with limited bioinformatics capabilities, this can be a fruitful way to reanalyze data from affected individuals where there is a strong clinical suspicion pointing to a specific gene. It is important that clinical laboratories gain extensive experience with complex variants to maximize the utility of whole-genome sequencing data, and some useful tips are provided in Note S8.

In clinically accredited NGS laboratories, studies have shown that some classes of SNV/indel have such high accuracy that validation using an orthogonal approach is not essential for the variant to be reported.[80–82] We speculate that the same will soon be true for SVs. Genome sequencing is the optimal approach to detect complex rearrangements, and so in our view, the failure of a poorly designed PCR-Sanger assay (or the inability to design suitable primers due to repeats) should ideally not delay reporting. At least two 100kGP participants were deceased before the results were able to be reported, making it hard to recontact families for appropriate follow-up. Also relevant to this discussion is one 100kGP participant (Family 15) harboring a *de novo* 1.22-Mb inversion that disrupts *SATB2* (MIM: 608148; GenBank: NM_001172509.2) in intron 2. This result was returned to the family before validation, on the basis of inspection in IGV (Figures S31A and S31B) and a phenotype (absent speech, history of persistent drooling, and dental abnormalities that include a gap between the maxillary central incisors) that was felt to be

a strong match with published cohorts of individuals with Glass syndrome (MIM: 612313).[83] Future studies measuring specificity and sensitivity of SVs across large cohorts such as the one described here will shed light on whether validation is always necessary.

In conclusion, our study demonstrates that, although relatively rare in comparison with other classes of variant, genomic inversions play an important role across a range of RDs. As well as many instances where our findings end long diagnostic odysseys, several results impact immediately on clinical management. This is most notable for the subset of diagnoses involving tumor suppressor genes, where cascade testing and disease surveillance are now being implemented. In 30% of families, the disrupted gene had previously been nominated as a strong candidate gene, as evidenced by prior testing using targeted methods. The lengthy delays in obtaining a diagnosis (even after genome sequencing became available) are therefore notable, given that detection of complex SVs is a significant raison d'être for genome sequencing. Consequently, it is important that future clinical analysis pipelines in the NHS and in similar programs worldwide are adapted to prioritize these types of variant (in combination with better workflows for confirmation and reporting) as genome sequencing becomes commonplace. Critical to the success of this project was the development of the SVRare and a large database of SVs called in a consistent way that facilitated the aggregation and subsequent filtering steps to minimize the number of candidate variants that required manual review.

Data and code availability

Illumina and PacBio (HiFi) genome sequencing and RNA-seq data relating to this study are held in the National Genomic Research Library (https://doi.org/10.6084/m9.figshare.4530893.v7). Details of how to access these data are available at www.genomicsengland.co.uk/research/academic/join-gecip. Access is currently provided via Amazon WorkSpaces. For academic researchers, host institutions also need to sign a formal agreement. SVRare code is available on github (see web resources).

Supplemental information

Supplemental information can be found online at https://doi.org/10.1016/j.ajhg.2024.04.018.

Acknowledgments

We thank all 100kGP participants for their involvement in this study. This manuscript is dedicated to Maria Bitner-Glindzicz, who recruited several of the families reported here. We also thank Stefan Mundlos for reviewing the *HOXD11-13* inversion. This research was made possible through access to the data and findings generated by the 100,000 Genomes Project. The 100,000 Genomes Project is managed by Genomics England Limited. The 100,000 Genomes Project is funded by the National Institute for Health Research and NHS England. The Wellcome Trust, Cancer Research UK, and the Medical Research Council have also funded research infrastructure. The 100,000 Genomes Project uses data provided by participants and collected by the NHS as part of their care and support. Additional funding was from the MRC (MR/W01761X/1) and the Oxford and Manchester NIHR Biomedical Research Centres (NIHR203308). H.H.U. is supported by The Leona M. and Harry B. Helmsley Charitable Trust, and support for M.J.S. is from USAMRAA CDMRP Neurofibromatosis Research Program, Investigator-Initiated Research Award (W81XWH1910334). We acknowledge the contributions of the Oxford GI biobank and the Oxford IBD cohort investigators. D.G.E. is supported by the Manchester National Institute for Health Research (NIHR) Biomedical Research Center (IS-BRC-1215-20007). D.B. and J.L. are supported by an NIHR Research Professorship to D.B. (RP-2016-07-011). J.L. is also supported by an Anniversary Fellowship awarded by the University of Southampton. A.J. and S.B. acknowledge the support of Solve-RD. The Solve-RD project received funding from the European Union's Horizon 2020 research and innovation program (779257).

Declaration of interests

H.H.U. declares research support or consultancy fees from Janssen, UCB Pharma, GSK, Eli Lilly, Bristol Myers Squibb BMS, OMass and Mestag. J.Y. is now employed by Novo Nordisk.

Received: November 12, 2023
Accepted: April 25, 2024
Published: May 21, 2024

Web resources

Clinical Genome Resource, https://clinicalgenome.org
DRAGEN pipeline, https://emea.illumina.com/products/by-type/informatics-products/basespace-sequence-hub/apps/edico-genome-inc-dragen-rna-pipeline.html
Genetic Mutation Age Estimator, https://shiny.wehi.edu.au/rafehi.h/mutation-dating
Genomic Laboratory Hubs, https://www.england.nhs.uk/genomics/genomic-laboratory-hubs
The Genotype-Tissue Expression (GTEx) Project, https://gtexportal.org
LiftOver tool, https://genome.ucsc.edu/cgi-bin/hgLiftOver
Manta, https://github.com/Illumina/manta
OMIM, https://www.omim.org
PanelApp, https://panelapp.genomicsengland.co.uk
Participant perspective for Family 16 (Genomics England Research Summit, May 2022), https://vimeo.com/709549654#t=13m40s
The R Project, https://www.r-project.org
Research registry, https://www.genomicsengland.co.uk/research/members/research-registry
SVRare, https://github.com/Oxford-Eye/SVRare-GEL
UniProt, https://www.uniprot.org

References

1. Feuk, L., Carson, A.R., and Scherer, S.W. (2006). Structural variation in the human genome. Nat. Rev. Genet. *7*, 85–97.
2. Pettersson, M., Grochowski, C.M., Wincent, J., Eisfeldt, J., Breman, A.M., Cheung, S.W., Krepischi, A.C.V., Rosenberg, C.,

Lupski, J.R., Ottosson, J., et al. (2020). Cytogenetically visible inversions are formed by multiple molecular mechanisms. Hum. Mutat. *41*, 1979–1998.

3. Burssed, B., Zamariolli, M., Bellucco, F.T., and Melaragno, M.I. (2022). Mechanisms of structural chromosomal rearrangement formation. Mol. Cytogenet. *15*, 23.
4. Jacquemont, M.L., Sanlaville, D., Redon, R., Raoul, O., Cormier-Daire, V., Lyonnet, S., Amiel, J., Le Merrer, M., Heron, D., de Blois, M.C., et al. (2006). Array-based comparative genomic hybridisation identifies high frequency of cryptic chromosomal rearrangements in patients with syndromic autism spectrum disorders. J. Med. Genet. *43*, 843–849.
5. Miller, D.T., Adam, M.P., Aradhya, S., Biesecker, L.G., Brothman, A.R., Carter, N.P., Church, D.M., Crolla, J.A., Eichler, E.E., Epstein, C.J., et al. (2010). Consensus statement: chromosomal microarray is a first-tier clinical diagnostic test for individuals with developmental disabilities or congenital anomalies. Am. J. Hum. Genet. *86*, 749–764.
6. 100000 Genomes Project Pilot Investigators, Smedley, D., Smith, K.R., Martin, A., Thomas, E.A., McDonagh, E.M., Cipriani, V., Ellingford, J.M., Arno, G., Tucci, A., et al. (2021). 100,000 Genomes Pilot on Rare-Disease Diagnosis in Health Care - Preliminary Report. N. Engl. J. Med. *385*, 1868–1880.
7. Turnbull, C., Scott, R.H., Thomas, E., Jones, L., Murugaesu, N., Pretty, F.B., Halai, D., Baple, E., Craig, C., Hamblin, A., et al. (2018). The 100 000 Genomes Project: bringing whole genome sequencing to the NHS. BMJ *361*, k1687.
8. Taylor, J.C., Martin, H.C., Lise, S., Broxholme, J., Cazier, J.B., Rimmer, A., Kanapin, A., Lunter, G., Fiddy, S., Allan, C., et al. (2015). Factors influencing success of clinical genome sequencing across a broad spectrum of disorders. Nat. Genet. *47*, 717–726.
9. Chen, X., Schulz-Trieglaff, O., Shaw, R., Barnes, B., Schlesinger, F., Källberg, M., Cox, A.J., Kruglyak, S., and Saunders, C.T. (2016). Manta: rapid detection of structural variants and indels for germline and cancer sequencing applications. Bioinformatics *32*, 1220–1222.
10. Newman, S., Hermetz, K.E., Weckselblatt, B., and Rudd, M.K. (2015). Next-generation sequencing of duplication CNVs reveals that most are tandem and some create fusion genes at breakpoints. Am. J. Hum. Genet. *96*, 208–220.
11. Chandrasekhar, A., Mroczkowski, H.J., Urraca, N., Gross, A., Bluske, K., Thorpe, E., Hagelstrom, R.T., Schonberg, S.A., Perry, D.L., Taft, R.J., et al. (2023). Genome sequencing detects a balanced pericentric inversion with breakpoints that impact the DMD and upstream region of POU3F4 genes. Am. J. Med. Genet. *194*, e63462.
12. Geng, C., Zhang, C., Li, P., Tong, Y., Zhu, B., He, J., Zhao, Y., Yao, F., Cui, L.Y., Liang, F., et al. (2023). Identification and characterization of two DMD pedigrees with large inversion mutations based on a long-read sequencing pipeline. Eur. J. Hum. Genet. *31*, 504–511.
13. Horton, A.E., Lunke, S., Sadedin, S., Fennell, A.P., and Stark, Z. (2023). Elusive variants in autosomal recessive disease: how can we improve timely diagnosis? Eur. J. Hum. Genet. *31*, 371–374.
14. Zaum, A.K., Nanda, I., Kress, W., and Rost, S. (2022). Detection of pericentric inversion with breakpoint in DMD by whole genome sequencing. Mol. Genet. Genomic Med. *10*, e2028.
15. Redin, C., Brand, H., Collins, R.L., Kammin, T., Mitchell, E., Hodge, J.C., Hanscom, C., Pillalamarri, V., Seabra, C.M., Abbott, M.A., et al. (2017). The genomic landscape of balanced cytogenetic abnormalities associated with human congenital anomalies. Nat. Genet. *49*, 36–45.
16. Pagnamenta, A.T., Yu, J., Evans, J., Twiss, P., Genomics England Research Consortium; and Musculoskeletal GeCIP MDT, Offiah, A.C., Wafik, M., Mehta, S.G., Javaid, M.K., et al. (2023). Conclusion of diagnostic odysseys due to inversions disrupting GLI3 and FBN1. J. Med. Genet. *60*, 505–510.
17. Rimmer, A., Phan, H., Mathieson, I., Iqbal, Z., Twigg, S.R.F., WGS500 Consortium, Wilkie, A.O.M., McVean, G., and Lunter, G. (2014). Integrating mapping-assembly- and haplotype-based approaches for calling variants in clinical sequencing applications. Nat. Genet. *46*, 912–918.
18. Martin, A.R., Williams, E., Foulger, R.E., Leigh, S., Daugherty, L.C., Niblock, O., Leong, I.U.S., Smith, K.R., Gerasimenko, O., Haraldsdottir, E., et al. (2019). PanelApp crowdsources expert knowledge to establish consensus diagnostic gene panels. Nat. Genet. *51*, 1560–1565.
19. Roller, E., Ivakhno, S., Lee, S., Royce, T., and Tanner, S. (2016). Canvas: versatile and scalable detection of copy number variants. Bioinformatics *32*, 2375–2377.
20. Yu, J., Szabo, A., Pagnamenta, A.T., Shalaby, A., Giacopuzzi, E., Taylor, J., Shears, D., Pontikos, N., Wright, G., Michaelides, M., et al. (2022). SVRare: discovering disease-causing structural variants in the 100K Genomes Project. Preprint at medRxiv. https://doi.org/10.1101/2021.10.15.21265069.
21. Robinson, J.T., Thorvaldsdóttir, H., Winckler, W., Guttman, M., Lander, E.S., Getz, G., and Mesirov, J.P. (2011). Integrative genomics viewer. Nat. Biotechnol. *29*, 24–26.
22. Patterson, M., Marschall, T., Pisanti, N., van Iersel, L., Stougie, L., Klau, G.W., and Schönhuth, A. (2015). WhatsHap: Weighted Haplotype Assembly for Future-Generation Sequencing Reads. J. Comput. Biol. *22*, 498–509.
23. Seibt, K.M., Schmidt, T., and Heitkam, T. (2018). FlexiDot: highly customizable, ambiguity-aware dotplots for visual sequence analyses. Bioinformatics *34*, 3575–3577.
24. Brechtmann, F., Mertes, C., Matusevičiūtė, A., Yépez, V.A., Avsec, Ž., Herzog, M., Bader, D.M., Prokisch, H., and Gagneur, J. (2018). OUTRIDER: A Statistical Method for Detecting Aberrantly Expressed Genes in RNA Sequencing Data. Am. J. Hum. Genet. *103*, 907–917.
25. Yepez, V.A., Mertes, C., Muller, M.F., Klaproth-Andrade, D., Wachutka, L., Fresard, L., Gusic, M., Scheller, I.F., Goldberg, P.F., Prokisch, H., et al. (2021). Detection of aberrant gene expression events in RNA sequencing data. Nat. Protoc. *16*, 1276–1296.
26. Wickham, H. (2016). ggplot2: Elegant Graphics for Data Analysis (Springer-Verlag).
27. Grinton, B.E., Robertson, E., Fearnley, L.G., Scheffer, I.E., Marson, A.G., O'Brien, T.J., Pickrell, W.O., Rees, M.I., Sisodiya, S.M., Balding, D.J., et al. (2022). A founder event causing a dominant childhood epilepsy survives 800 years through weak selective pressure. Am. J. Hum. Genet. *109*, 2080–2087.
28. Pagnamenta, A.T., Kaiyrzhanov, R., Zou, Y., Da'as, S.I., Maroofian, R., Donkervoort, S., Dominik, N., Lauffer, M., Ferla, M.P., Orioli, A., et al. (2021). An ancestral 10-bp repeat expansion in VWA1 causes recessive hereditary motor neuropathy. Brain *144*, 584–600.

29. Gandolfo, L.C., Bahlo, M., and Speed, T.P. (2014). Dating rare mutations from small samples with dense marker data. Genetics *197*, 1315–1327.
30. Hamdan, F.F., Myers, C.T., Cossette, P., Lemay, P., Spiegelman, D., Laporte, A.D., Nassif, C., Diallo, O., Monlong, J., Cadieux-Dion, M., et al. (2017). High Rate of Recurrent De Novo Mutations in Developmental and Epileptic Encephalopathies. Am. J. Hum. Genet. *101*, 664–685.
31. Hashim, M., Stewart, H., Yu, J., Banos-Pinero, B., Genomics England Research Consortium, Pagnamenta, A.T., and Taylor, J.C. (2023). Genome sequencing identifies KMT2E-disrupting cryptic structural variant in a female with O'Donnell-Luria-Rodan syndrome. Clin. Genet. *104*, 390–392.
32. Hyder, Z., Calpena, E., Pei, Y., Tooze, R.S., Brittain, H., Twigg, S.R.F., Cilliers, D., Morton, J.E.V., McCann, E., Weber, A., et al. (2021). Evaluating the performance of a clinical genome sequencing program for diagnosis of rare genetic disease, seen through the lens of craniosynostosis. Genet. Med. *23*, 2360–2368.
33. Carvalho, C.M.B., Ramocki, M.B., Pehlivan, D., Franco, L.M., Gonzaga-Jauregui, C., Fang, P., McCall, A., Pivnick, E.K., Hines-Dowell, S., Seaver, L.H., et al. (2011). Inverted genomic segments and complex triplication rearrangements are mediated by inverted repeats in the human genome. Nat. Genet. *43*, 1074–1081.
34. Grochowski, C.M., Bengtsson, J.D., Du, H., Gandhi, M., Lun, M.Y., Mehaffey, M.G., Park, K., Höps, W., Benito-Garagorri, E., Hasenfeld, P., et al. (2023). Break-induced replication underlies formation of inverted triplications and generates unexpected diversity in haplotype structures. Preprint at bioRxiv. https://doi.org/10.1101/2023.10.02.560172.
35. Fontana, L., Tabano, S., Maitz, S., Colapietro, P., Garzia, E., Gerli, A.G., Sirchia, S.M., and Miozzo, M. (2021). Clinical and Molecular Diagnosis of Beckwith-Wiedemann Syndrome with Single- or Multi-Locus Imprinting Disturbance. Int. J. Mol. Sci. *22*, 3445.
36. Kay, A.C., Wells, J., Hallowell, N., and Goriely, A. (2023). Providing recurrence risk counselling for parents after diagnosis of a serious genetic condition caused by an apparently de novo mutation in their child: a qualitative investigation of the PREGCARE strategy with UK clinical genetics practitioners. J. Med. Genet. *60*, 925–931.
37. Wright, C.F., Campbell, P., Eberhardt, R.Y., Aitken, S., Perrett, D., Brent, S., Danecek, P., Gardner, E.J., Chundru, V.K., Lindsay, S.J., et al. (2023). Genomic Diagnosis of Rare Pediatric Disease in the United Kingdom and Ireland. N. Engl. J. Med. *388*, 1559–1571.
38. Tenorio-Castano, J., Gomez, A.S., Coronado, M., Rodriguez-Martin, P., Parra, A., Pascual, P., Cazalla, M., Gallego, N., Arias, P., Morales, A.V., et al. (2023). Lamb-Shaffer syndrome: 20 Spanish patients and literature review expands the view of neurodevelopmental disorders caused by SOX5 haploinsufficiency. Clin. Genet. *104*, 637–647.
39. Hocking, L.J., Andrews, C., Armstrong, C., Ansari, M., Baty, D., Berg, J., Bradley, T., Clark, C., Diamond, A., Doherty, J., et al. (2023). Genome sequencing with gene panel-based analysis for rare inherited conditions in a publicly funded healthcare system: implications for future testing. Eur. J. Hum. Genet. *31*, 231–238.
40. Moller, R.S., Kubart, S., Hoeltzenbein, M., Heye, B., Vogel, I., Hansen, C.P., Menzel, C., Ullmann, R., Tommerup, N., Ropers, H.H., et al. (2008). Truncation of the Down syndrome candidate gene DYRK1A in two unrelated patients with microcephaly. Am. J. Hum. Genet. *82*, 1165–1170.
41. Wang, X., Wu, H., Sun, H., Wang, L., and Chen, L. (2022). ARID2, a Rare Cause of Coffin-Siris Syndrome: A Clinical Description of Two Cases. Front. Pediatr. *10*, 911954.
42. Posey, J.E., Harel, T., Liu, P., Rosenfeld, J.A., James, R.A., Coban Akdemir, Z.H., Walkiewicz, M., Bi, W., Xiao, R., Ding, Y., et al. (2017). Resolution of Disease Phenotypes Resulting from Multilocus Genomic Variation. N. Engl. J. Med. *376*, 21–31.
43. Yang, Y., Muzny, D.M., Xia, F., Niu, Z., Person, R., Ding, Y., Ward, P., Braxton, A., Wang, M., Buhay, C., et al. (2014). Molecular findings among patients referred for clinical whole-exome sequencing. JAMA *312*, 1870–1879.
44. Watson, C.M., Crinnion, L.A., Harrison, S.M., Lascelles, C., Antanaviciute, A., Carr, I.M., Bonthron, D.T., and Sheridan, E. (2016). A Chromosome 7 Pericentric Inversion Defined at Single-Nucleotide Resolution Using Diagnostic Whole Genome Sequencing in a Patient with Hand-Foot-Genital Syndrome. PLoS One *11*, e0157075.
45. Perez-Becerril, C., Burghel, G.J., Hartley, C., Rowlands, C.F., Evans, D.G., and Smith, M.J. (2024). Improved sensitivity for detection of pathogenic variants in familial NF2-related schwannomatosis. J. Med. Genet. *61*, 452–458.
46. Bovee, J.V. (2008). Multiple osteochondromas. Orphanet J. Rare Dis. *3*, 3.
47. Shears, D.J., Offiah, A., Rutland, P., Sirimanna, T., Bitner-Glindzicz, M., and Hall, C. (2004). Kantaputra mesomelic dysplasia: a second reported family. Am. J. Med. Genet. *128A*, 6–11.
48. Spitz, F., Gonzalez, F., and Duboule, D. (2003). A global control region defines a chromosomal regulatory landscape containing the HoxD cluster. Cell *113*, 405–417.
49. Kantaputra, P.N., Klopocki, E., Hennig, B.P., Praphanphoj, V., Le Caignec, C., Isidor, B., Kwee, M.L., Shears, D.J., and Mundlos, S. (2010). Mesomelic dysplasia Kantaputra type is associated with duplications of the HOXD locus on chromosome 2q. Eur. J. Hum. Genet. *18*, 1310–1314.
50. Kantaputra, P.N., Gorlin, R.J., and Langer, L.O., Jr. (1992). Dominant mesomelic dysplasia, ankle, carpal, and tarsal synostosis type: a new autosomal dominant bone disorder. Am. J. Med. Genet. *44*, 730–737.
51. Fujimoto, M., Kantaputra, P.N., Ikegawa, S., Fukushima, Y., Sonta, S., Matsuo, M., Ishida, T., Matsumoto, T., Kondo, S., Tomita, H., et al. (1998). The gene for mesomelic dysplasia Kantaputra type is mapped to chromosome 2q24-q32. J. Hum. Genet. *43*, 32–36.
52. Goff, D.J., and Tabin, C.J. (1997). Analysis of Hoxd-13 and Hoxd-11 misexpression in chick limb buds reveals that Hox genes affect both bone condensation and growth. Development *124*, 627–636.
53. Cuinat, S., Nizon, M., Isidor, B., Stegmann, A., van Jaarsveld, R.H., van Gassen, K.L., van der Smagt, J.J., Volker-Touw, C.M.L., Holwerda, S.J.B., Terhal, P.A., et al. (2022). Loss-of-function variants in SRRM2 cause a neurodevelopmental disorder. Genet. Med. *24*, 1774–1780.
54. Kaplanis, J., Samocha, K.E., Wiel, L., Zhang, Z., Arvai, K.J., Eberhardt, R.Y., Gallone, G., Lelieveld, S.H., Martin, H.C., McRae, J.F., et al. (2020). Evidence for 28 genetic disorders discovered by combining healthcare and research data. Nature *586*, 757–762.
55. Pagnamenta, A.T., Yu, J., Willis, T.A., Hashim, M., Seaby, E.G., Walker, S., Xian, J., Cheng, E.W.Y., Tavares, A.L.T., Forzano, F., et al. (2023). A Palindrome-Like Structure on

16p13.3 Is Associated with the Formation of Complex Structural Variations and SRRM2 Haploinsufficiency. Hum. Mutat., 1–9.
56. Brand, H., Collins, R.L., Hanscom, C., Rosenfeld, J.A., Pillalamarri, V., Stone, M.R., Kelley, F., Mason, T., Margolin, L., Eggert, S., et al. (2015). Paired-Duplication Signatures Mark Cryptic Inversions and Other Complex Structural Variation. Am. J. Hum. Genet. *97*, 170–176.
57. Liu, Q., Hesson, L.B., Nunez, A.C., Packham, D., Williams, R., Ward, R.L., and Sloane, M.A. (2016). A cryptic paracentric inversion of MSH2 exons 2-6 causes Lynch syndrome. Carcinogenesis *37*, 10–17.
58. Brennan, B., Hemmings, C.T., Clark, I., Yip, D., Fadia, M., and Taupin, D.R. (2017). Universal molecular screening does not effectively detect Lynch syndrome in clinical practice. Therap. Adv. Gastroenterol. *10*, 361–371.
59. Speed, D., and Balding, D.J. (2015). Relatedness in the post-genomic era: is it still useful? Nat. Rev. Genet. *16*, 33–44.
60. Morak, M., Steinke-Lange, V., Massdorf, T., Benet-Pages, A., Locher, M., Laner, A., Kayser, K., Aretz, S., and Holinski-Feder, E. (2020). Prevalence of CNV-neutral structural genomic rearrangements in MLH1, MSH2, and PMS2 not detectable in routine NGS diagnostics. Fam. Cancer *19*, 161–167.
61. Wagner, A., van der Klift, H., Franken, P., Wijnen, J., Breukel, C., Bezrookove, V., Smits, R., Kinarsky, Y., Barrows, A., Franklin, B., et al. (2002). A 10-Mb paracentric inversion of chromosome arm 2p inactivates MSH2 and is responsible for hereditary nonpolyposis colorectal cancer in a North-American kindred. Genes Chromosomes Cancer *35*, 49–57.
62. Rhees, J., Arnold, M., and Boland, C.R. (2014). Inversion of exons 1-7 of the MSH2 gene is a frequent cause of unexplained Lynch syndrome in one local population. Fam. Cancer *13*, 219–225.
63. Lebo, R.V., Ikuta, T., Milunsky, J.M., and Milunsky, A. (2001). Rett syndrome from quintuple and triple deletions within the MECP2 deletion hotspot region. Clin. Genet. *59*, 406–417.
64. Villard, L. (2007). MECP2 mutations in males. J. Med. Genet. *44*, 417–423.
65. Geerdink, N., Rotteveel, J.J., Lammens, M., Sistermans, E.A., Heikens, G.T., Gabreëls, F.J.M., Mullaart, R.A., and Hamel, B.C.J. (2002). MECP2 mutation in a boy with severe neonatal encephalopathy: clinical, neuropathological and molecular findings. Neuropediatrics *33*, 33–36.
66. Beskorovainaya, T., Konovalov, F., Demina, N., Shchagina, O., Pashchenko, M., Kanivets, I., Pyankov, D., Ryzhkova, O., and Polyakov, A. (2021). Case Report: Complicated Molecular Diagnosis of MECP2 Gene Structural Rearrangement in a Proband with Rett Syndrome. J. Autism Dev. Disord. *51*, 2159–2163.
67. de Bruijn, S.E., Fiorentino, A., Ottaviani, D., Fanucchi, S., Melo, U.S., Corral-Serrano, J.C., Mulders, T., Georgiou, M., Rivolta, C., Pontikos, N., et al. (2020). Structural Variants Create New Topological-Associated Domains and Ectopic Retinal Enhancer-Gene Contact in Dominant Retinitis Pigmentosa. Am. J. Hum. Genet. *107*, 802–814.
68. Richards, S., Aziz, N., Bale, S., Bick, D., Das, S., Gastier-Foster, J., Grody, W.W., Hegde, M., Lyon, E., Spector, E., et al. (2015). Standards and guidelines for the interpretation of sequence variants: a joint consensus recommendation of the American College of Medical Genetics and Genomics and the Association for Molecular Pathology. Genet. Med. *17*, 405–424.
69. Amandio, A.R., Beccari, L., Lopez-Delisle, L., Mascrez, B., Zakany, J., Gitto, S., and Duboule, D. (2021). Sequential in cis mutagenesis in vivo reveals various functions for CTCF sites at the mouse HoxD cluster. Genes Dev. *35*, 1490–1509.
70. Hoovers, J.M., Kalikin, L.M., Johnson, L.A., Alders, M., Redeker, B., Law, D.J., Bliek, J., Steenman, M., Benedict, M., Wiegant, J., et al. (1995). Multiple genetic loci within 11p15 defined by Beckwith-Wiedemann syndrome rearrangement breakpoints and subchromosomal transferable fragments. Proc. Natl. Acad. Sci. USA *92*, 12456–12460.
71. Aref-Eshghi, E., Bend, E.G., Colaiacovo, S., Caudle, M., Chakrabarti, R., Napier, M., Brick, L., Brady, L., Carere, D.A., Levy, M.A., et al. (2019). Diagnostic Utility of Genome-wide DNA Methylation Testing in Genetically Unsolved Individuals with Suspected Hereditary Conditions. Am. J. Hum. Genet. *104*, 685–700.
72. Carvalho, C.M.B., Coban-Akdemir, Z., Hijazi, H., Yuan, B., Pendleton, M., Harrington, E., Beaulaurier, J., Juul, S., Turner, D.J., Kanchi, R.S., et al. (2019). Interchromosomal template-switching as a novel molecular mechanism for imprinting perturbations associated with Temple syndrome. Genome Med. *11*, 25.
73. Scharf, F., Leal Silva, R.M., Morak, M., Hastie, A., Pickl, J.M.A., Sendelbach, K., Gebhard, C., Locher, M., Laner, A., Steinke-Lange, V., et al. (2022). Constitutional chromothripsis of the APC locus as a cause of genetic predisposition to colon cancer. J. Med. Genet. *59*, 976–983.
74. Best, S., Inglehearn, C.F., Watson, C.M., Toomes, C., Wheway, G., and Johnson, C.A. (2022). Unlocking the potential of the UK 100,000 Genomes Project-lessons learned from analysis of the "Congenital Malformations caused by Ciliopathies" cohort. Am. J. Med. Genet. C Semin. Med. Genet. *190*, 5–8.
75. Brandt, T., Sack, L.M., Arjona, D., Tan, D., Mei, H., Cui, H., Gao, H., Bean, L.J.H., Ankala, A., Del Gaudio, D., et al. (2020). Adapting ACMG/AMP sequence variant classification guidelines for single-gene copy number variants. Genet. Med. *22*, 336–344.
76. Gardner, E.J., Sifrim, A., Lindsay, S.J., Prigmore, E., Rajan, D., Danecek, P., Gallone, G., Eberhardt, R.Y., Martin, H.C., Wright, C.F., et al. (2021). Detecting cryptic clinically relevant structural variation in exome-sequencing data increases diagnostic yield for developmental disorders. Am. J. Hum. Genet. *108*, 2186–2194.
77. Evers, J.M.G., Laskowski, R.A., Bertolli, M., Clayton-Smith, J., Deshpande, C., Eason, J., Elmslie, F., Flinter, F., Gardiner, C., Hurst, J.A., et al. (2017). Structural analysis of pathogenic mutations in the DYRK1A gene in patients with developmental disorders. Hum. Mol. Genet. *26*, 519–526.
78. Sanchis-Juan, A., Megy, K., Stephens, J., Armirola Ricaurte, C., Dewhurst, E., Low, K., French, C.E., Grozeva, D., Stirrups, K., Erwood, M., et al. (2023). Genome sequencing and comprehensive rare-variant analysis of 465 families with neurodevelopmental disorders. Am. J. Hum. Genet. *110*, 1343–1355.
79. Moore, A.R., Yu, J., Pei, Y., Cheng, E.W.Y., Taylor Tavares, A.L., Walker, W.T., Thomas, N.S., Kamath, A., Ibitoye, R., Josifova, D., et al. (2023). Use of genome sequencing to hunt for cryptic second-hit variants: analysis of 31 cases recruited to the 100 000 Genomes Project. J. Med. Genet. *60*, 1235–1244.
80. Lincoln, S.E., Truty, R., Lin, C.F., Zook, J.M., Paul, J., Ramey, V.H., Salit, M., Rehm, H.L., Nussbaum, R.L., and Lebo, M.S. (2019). A Rigorous Interlaboratory Examination of the Need

to Confirm Next-Generation Sequencing-Detected Variants with an Orthogonal Method in Clinical Genetic Testing. J. Mol. Diagn. *21*, 318–329.

81. Bauer, P., Kandaswamy, K.K., Weiss, M.E.R., Paknia, O., Werber, M., Bertoli-Avella, A.M., Yüksel, Z., Bochinska, M., Oprea, G.E., Kishore, S., et al. (2019). Development of an evidence-based algorithm that optimizes sensitivity and specificity in ES-based diagnostics of a clinically heterogeneous patient population. Genet. Med. *21*, 53–61.

82. Beck, T.F., Mullikin, J.C., NISC Comparative Sequencing Program, and Biesecker, L.G. (2016). Systematic Evaluation of Sanger Validation of Next-Generation Sequencing Variants. Clin. Chem. *62*, 647–654.

83. Zarate, Y.A., Smith-Hicks, C.L., Greene, C., Abbott, M.A., Siu, V.M., Calhoun, A.R.U.L., Pandya, A., Li, C., Sellars, E.A., Kaylor, J., et al. (2018). Natural history and genotype-phenotype correlations in 72 individuals with SATB2-associated syndrome. Am. J. Med. Genet. *176*, 925–935.

The American Journal of Human Genetics, Volume *111*

Supplemental information

The impact of inversions across 33,924 families with rare disease from a national genome sequencing project

Alistair T. Pagnamenta, Jing Yu, Susan Walker, Alexandra J. Noble, Jenny Lord, Prasun Dutta, Mona Hashim, Carme Camps, Hannah Green, Smrithi Devaiah, Lina Nashef, Jason Parr, Carl Fratter, Rana Ibnouf Hussein, Sarah J. Lindsay, Fiona Lalloo, Benito Banos-Pinero, David Evans, Lucy Mallin, Adrian Waite, Julie Evans, Andrew Newman, Zoe Allen, Cristina Perez-Becerril, Gavin Ryan, Rachel Hart, John Taylor, Tina Bedenham, Emma Clement, Ed Blair, Eleanor Hay, Francesca Forzano, Jenny Higgs, Natalie Canham, Anirban Majumdar, Meriel McEntagart, Nayana Lahiri, Helen Stewart, Sarah Smithson, Eduardo Calpena, Adam Jackson, Siddharth Banka, Hannah Titheradge, Ruth McGowan, Julia Rankin, Charles Shaw-Smith, D. Gareth Evans, George J. Burghel, Miriam J. Smith, Emily Anderson, Rajesh Madhu, Helen Firth, Sian Ellard, Paul Brennan, Claire Anderson, Doug Taupin, Mark T. Rogers, Jackie A. Cook, Miranda Durkie, James E. East, Darren Fowler, Louise Wilson, Rebecca Igbokwe, Alice Gardham, Ian Tomlinson, Diana Baralle, Holm H. Uhlig, and Jenny C. Taylor

Note S1: Additional background on MLPA testing.

In addition to array-based testing for genome-wide copy number alterations (CNV), multiplex ligation-dependent probe amplification (MLPA) is another technology commonly used in clinical testing laboratories. This targeted method is based on a ligation reaction, followed by multiplex PCR, with typically up to 40 probes in any one mix. The method is suitable for conditions where there are a small number of strong candidate genes. Although dependant on probe design, single exon CNVs can be picked up robustly. In some cases, where the precise nature of the rearrangement is known, probes that target copy-neutral SVs can also be incorporated. For instance, a common 10Mb inversion involving *MSH2*[1] is captured by the latest commercial MLPA product (e.g. SALSA probemix for MLH1/MSH2, P003-D1; MRC Holland). However, this remains an exception rather than the rule, and the vast majority of balanced rearrangements will be missed by MLPA, as well as by arrays.

Note S2: Additional background about the 100,000 Genomes Project.

The 100kGP is a national genome sequencing study run by Genomics England, a company owned by the Department of Health and Social Care. The project was initiated in 2012 and most of the sequencing was completed in 2018. Although many thousands of primary/secondary results have already been returned to participants, analysis of data is ongoing through Genomics England's diagnostic discovery route. There have now been over 1,000 approved research projects utilizing this data (https://research.genomicsengland.co.uk/research-registry), and over 1,100 researchers active within the framework of the National Genomic Research Library. Building on the success of the 100kGP, genome sequencing is now being offered routinely within the NHS for a wide range of clinical indications. In the main RD programme of the 100kGP, the diagnostic rate is estimated to be 20-25%, and varies according to the clinical indication, which further highlights the importance of improving variant detection/prioritisation strategies in the clinical analysis pipeline. The analysis described in this study is covered by project RR693 in the Genomics England Research Registry ("The impact of germline inversions in the rare disease arm of the 100,000 Genomes Project") which was submitted in March 2022 and has been approved by Genomics England. The majority of variants reported here were entered into the Diagnostic Discovery pathway over a 9 month period between September 2021 and June 2022.

Note S3: Additional example of individual with phenotype blending.

In Family 12, the proband was already known to have achondroplasia and prior testing of *FGFR3* had uncovered a maternal NM_000142.5:c.1138G>A, p.(Gly380Arg) variant. This is a well-known recurrent pathogenic mutation listed in ClinVar with over 40 submissions (VCV000016327.104). This family had been recruited to the 100kGP as it was felt that *FGFR3* did not explain all the individual's clinical features. Identification of a complex SV in *EFTUD2* led to the hypothesis that this variant may be acting together with the missense in *FGFR3* to result in the participant's phenotype. The SV in *EFTUD2* was shown to have arisen *de novo* and comprised a deletion of 6.5kb, with two retained internal segments of 252bp and 236bp (Figure S12).

Note S4: Enzyme and immunohistochemistry for *PDHA1.*

In Family 41, an inversion call involving exons 6-9 of *PDHA1* (NM_000284.4) was in fact the proximal 3x end of a duplication-triplication (Figure S5). This SV was identified in a 100kGP participant with exercise intolerance, intellectual disability and white matter abnormalities and so compatible with Pyruvate dehydrogenase E1-alpha deficiency [MIM #312170]. Interpretation was more complex due to the structural ambiguity. This SV had previously been picked up independently by a clinical laboratory using array-CGH and interpreted as a duplication of uncertain significance. Pyruvate

dehydrogenase activity was measured in cultured fibroblasts from the proband and the mean activity of 0.54 nmol/mg protein/min was marginally below the normal range of 0.6-0.9. In a female, there is always the possibility of normal, or near normal, activity with heterozygosity for a *PDHA1* mutation and a pattern of X-inactivation favouring expression of the normal X chromosome, however, this is relatively uncommon. In light of the Xp22 rearrangement in this patient, cells were also analysed with an antibody to the Ela subunit to see if there was any evidence of mosaicism. This method permits small populations of deficient cells to be detected. However, the cells were all uniformly positive with the antibody. Together with the near-normal enzyme activity, these results suggest that the duplication likely has no consequences as far as *PDHA1* is concerned.

Note S5: Examples from the 100kGP of complex SVs in autosomal recessive disease associated genes.

We recently described a 100kGP participant with generalized arterial calcification of infancy [MIM #208000] who harboured interlinked/inverted duplications that disrupt *ENPP1*.[2] The variant was identified following a manual search at a specific locus that was prompted by clinical suspicion. Another (unpublished) example from the 100kGP involves a previously reported complex deletion-inversion involving *OCA2*[3] found *in trans* with NM_000275.3:c.1441G>A (p.Ala481Thr, VCV000000954.45) in sisters with developmental macular and foveal dystrophy. In that case, the SV was identified via use of a SV-haplotype tagging SNV (rs374519281).

Note S6: Additional examples of cryptic *APC* variants.

Inversions that disrupt *APC* have been reported previously and these have been detected using a variety of methods such as by high coverage NGS capture of intronic regions[4] or by nanopore sequencing.[5] An earlier study also used a cDNA approach to detect structural variants in 4/49 potential FAP families,[6] suggesting that SVs involving this gene are not uncommon. Another study identified two individuals with adenomatous polyposis likely due to intronic SVA element insertions that affect *APC*.[7] Other recent reports highlight that genome sequencing can detect deep intronic variants that lead to the introduction of pseudoexons in the *APC* transcript.[8]

Note S7: Other examples of complex SVs reported with incomplete interpretation.

In a recent study on inherited eye diseases[9], Fig. 3 shows a complex deletion-inverted non-tandem duplication in *EYS*. Careful review of the images shown suggests that the authors interpretation may be incomplete. Due to the presence of a 31kb duplication which cannot be spanned with short reads, the middle "Segment C" could be both ways around and the split read pattern would be identical. In addition, the extra copy of exon 31 is on the other strand from the rest of the gene and we suspect is unlikely to be spliced into the RNA transcript.

In another recent report, a rearrangement disrupting *SMARCAL1*, found *in trans* with a frameshift variant in a patient with Schimke immune-osseous dysplasia, was interpreted as inversion.[10] Again, closer scrutiny of the published IGV image suggests that a non-tandem duplication inserted in an inverted orientation could also potentially explain that short-read data.

Note S8: Additional tips on manual review of read alignments.

Read alignments files (BAM or CRAM format) can be loaded up for manual analysis using IGV software that is freely available (https://igv.org/doc/desktop). When viewing read alignments, it is important to use the IGV setting "color alignments by insert size and pair orientation" or the more stringent "color alignments by pair orientation" such that +ve to +ve strand read-pair mappings are highlighted in teal, whilst -ve to -ve mappings are in blue. For balanced SVs, the green and blue should point inwards towards a discreet breakpoint. Where copy number changes are involved, split-reads should

point to the breakpoint but only in the direction going from higher to lower coverage. For large genes, it can also be helpful to load up structural vcf file in IGV as well, to help guide the analyst towards which regions of the gene are most critical to check in more detail. The “show mismatched bases” option can as also be turned off to further assist visualisation of SVs in read alignment data. We hope that the collection of IGV screenshots provided here, in combination with access to alignment data via the National Genomic Research Library, can be a useful learning resource for genome analysts new to structural variation.

Visualisation is critical to help facilitate the correct interpretation of complex SVs and many ways to illustrate such rearrangements have been proposed. Schematic diagrams showing the relative copy number states and the positions/directions of the split read-pairs were crucial in several cases for determining if additional configurations were potential solutions to the short-read data. For Family 43, we felt that instead of Circos plot, a “subway” plot gave a better representation and showed in an intuitive way that the complex SV structure was a translocation that could be confirmed by karyotyping, which proved to be correct. In contrast, for the cases involving *MECP2,* annotated dotplots generated from single PacBio reads were used to help demonstrate the precise configuration of the rearrangement and aid interpretation. This worked well for Family 33 (Figure 4B) and comparison to a similar plot produced for Family 47 (Figure S28) helped confirm that the same hotspot region was involved. We anticipate that future developments in this area should help automate SV reconstruction (i.e. variant calling algorithms that report complete structures, not just breakpoints) and aid conceptualisation of complex SVs.

Supplemental Figures

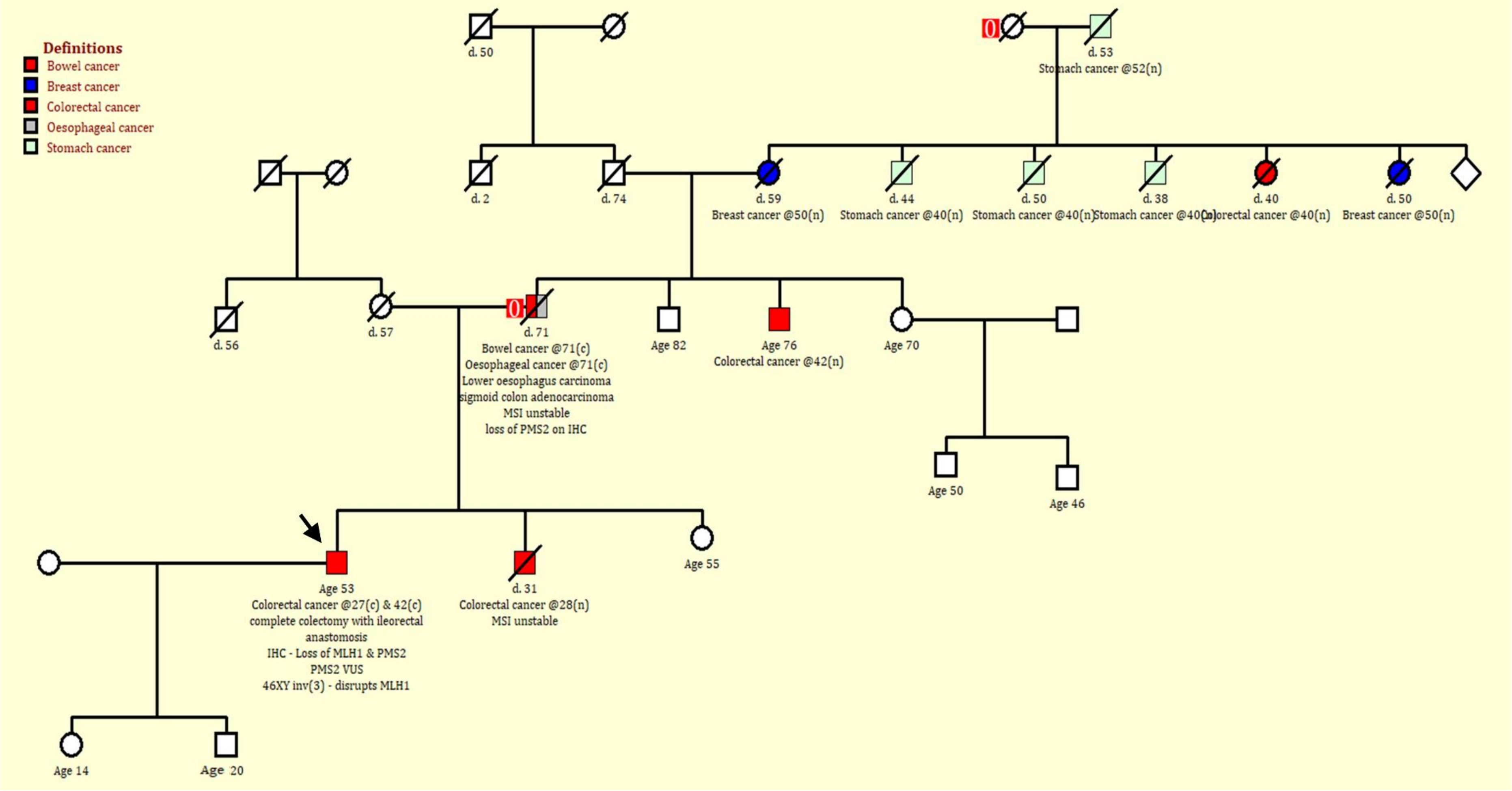

Figure S1: Pedigree for Family 19 harbouring a 30.7Mb inversion that disrupts *MLH1*. The 53 year old male labelled with the arrow is the 100k Genomes Project participant.

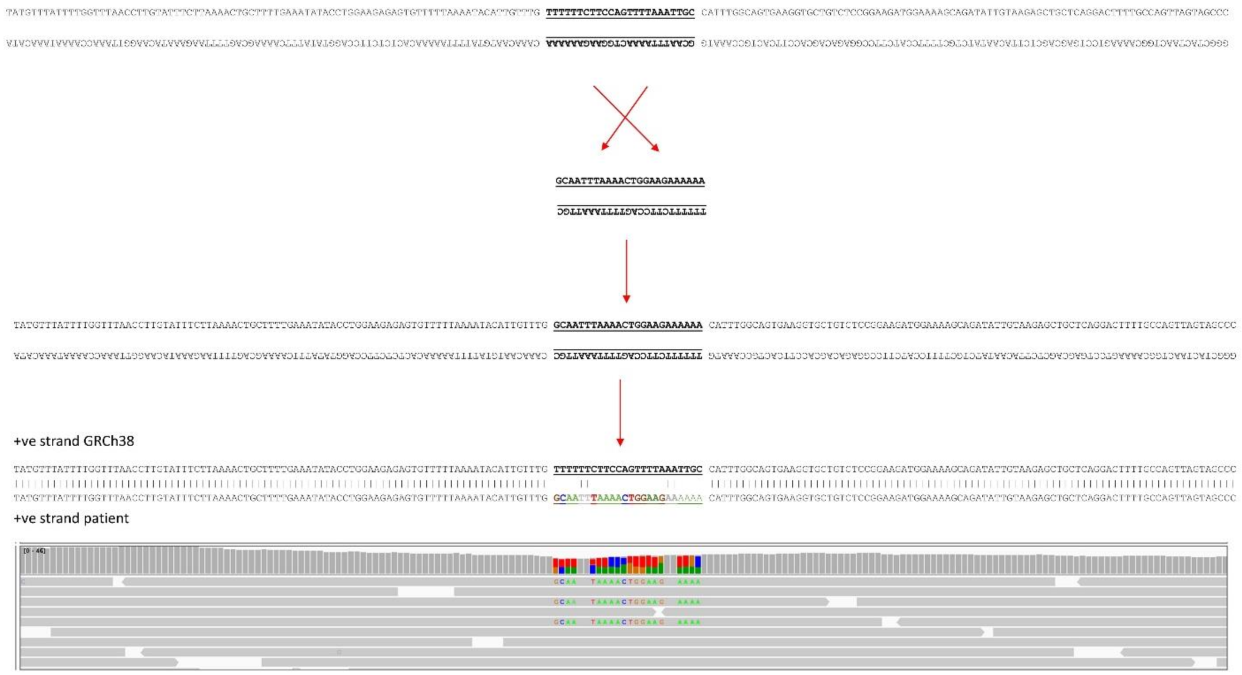

Figure S2: Schematic diagram illustrating how the 24bp inversion seen in Family 45 can result in the pattern of mismatches seen in the read alignments shown in IGV. For 4 positions, the inversion does not change the DNA base present.

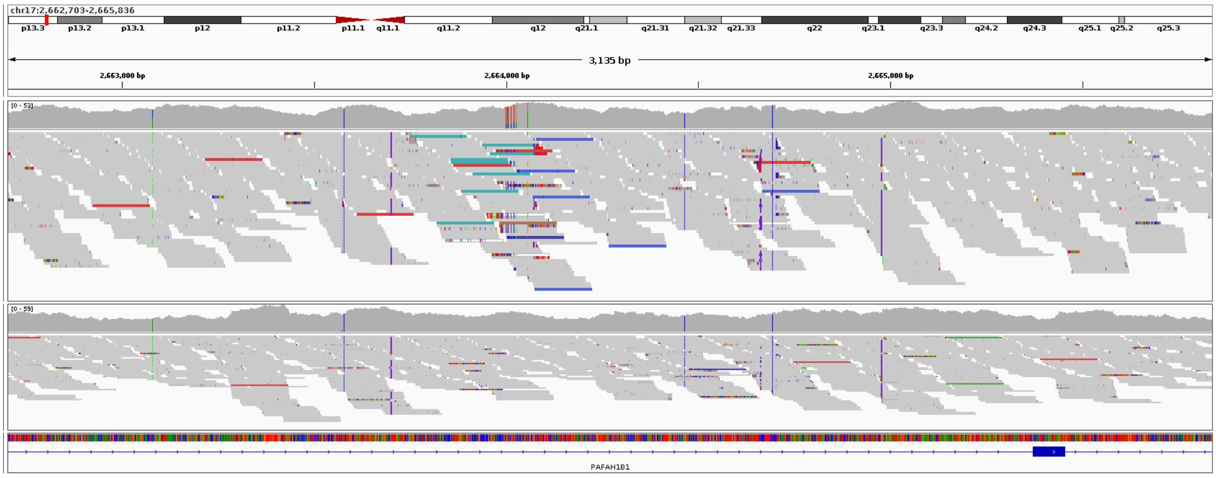

Figure S3: Read alignments supporting a 256kb inversion involving *PAFAH1B1* (NM_000430.4) in Family 13. Split read-pairs shown in blue (-ve to -ve strand) and teal (+ve to +ve strand) are seen for the proband (upper) but not in the mother (lower). As the proximal breakpoint lies in intron 2, this inversion is likely to disrupt gene function. The high degree of phenotypic specificity lends additional weight supporting this inversion to be responsible for the patient’s diagnosis.

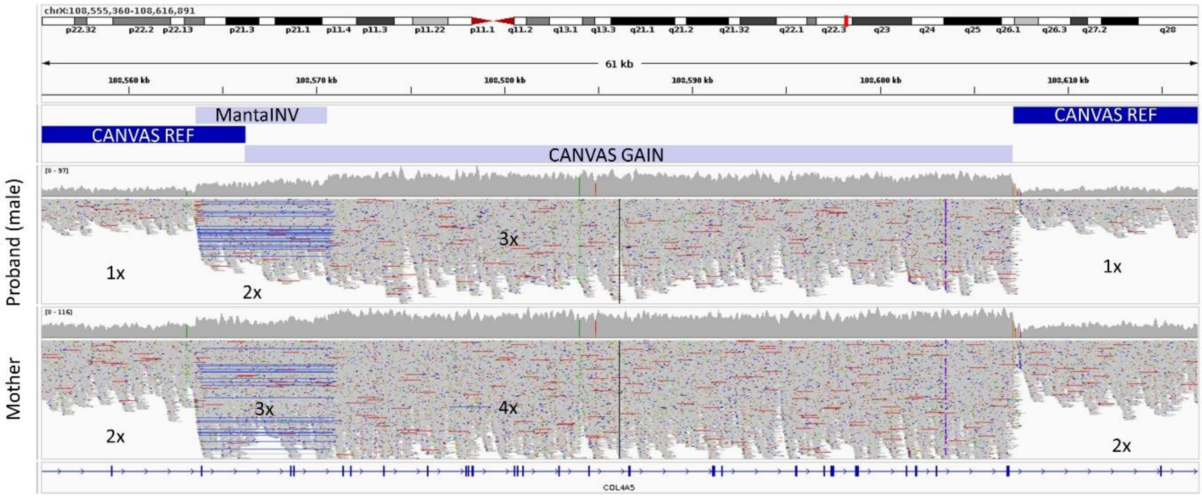

Figure S4: Read alignments supporting complex duplication-triplication involving *COL4A5* in Family 25. The 7kb MantaINV call involving 3 coding exons is shown as thin horizontal blue lines which denote -ve to -ve strand mapping split read pairs. Reads are shown using the squished view option in IGV. The algorithmic SV calls are shown in the top track and the relative copy number states are labelled. The rearrangement was present in the similarly affected mother (lower track).

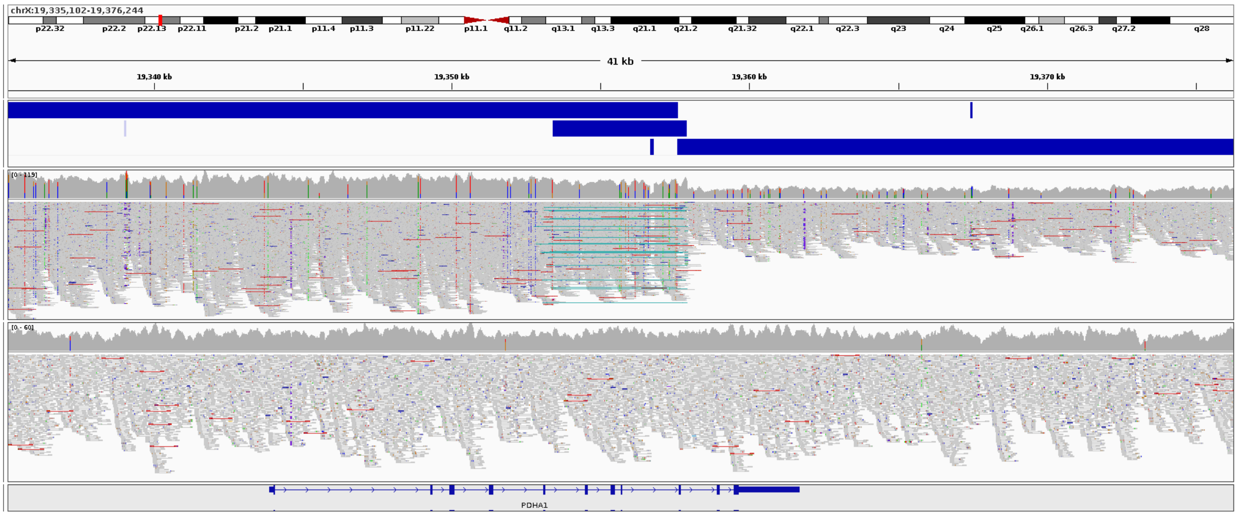

Figure S5: Read alignments supporting complex duplication-triplication involving *PDHA1* in Family 41. The 4.5kb MantaINV call involving 4 coding exons is shown as a horizontal blue bar in the upper track. Read alignments for the proband highlight +ve to +ve strand read pairs (in teal) which, combined with the increased coverage, are indicative of a duplication-triplication. The rearrangement was not seen in the unaffected mother (lower track). Testing of the father was not possible.

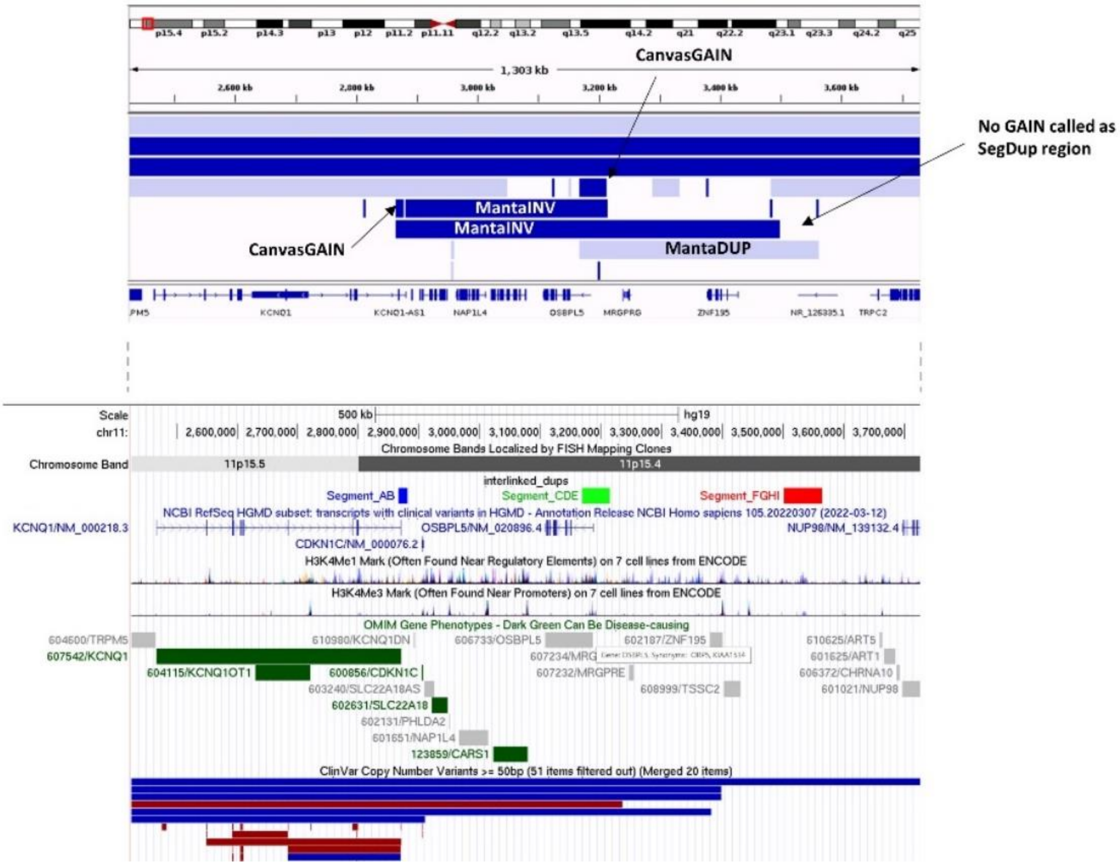

Figure S6: Complex *de novo* SV in Family 44 involving three interlinked duplications on chromosome 11p15.4. The SV was identified due to two overlapping MantaINV calls, as shown in the IGV screenshot. The genomic window shown in IGV is aligned to the UCSC genome browser image below. The smallest of the duplicated segments (AB; blue) lies 24kb downstream of *CDKN1C* and also close to *KCNQ1OT1* (KCNQ1-opposite strand/antisense transcript 1) which has a critical role in regulating *CDKN1C*. An interactive UCSC session is available at http://genome.ucsc.edu/s/AlistairP/CDKN1C_duplications. Coordinates shown here are based on GRCh37, but are lifted over to GRCh38 for Table S2.

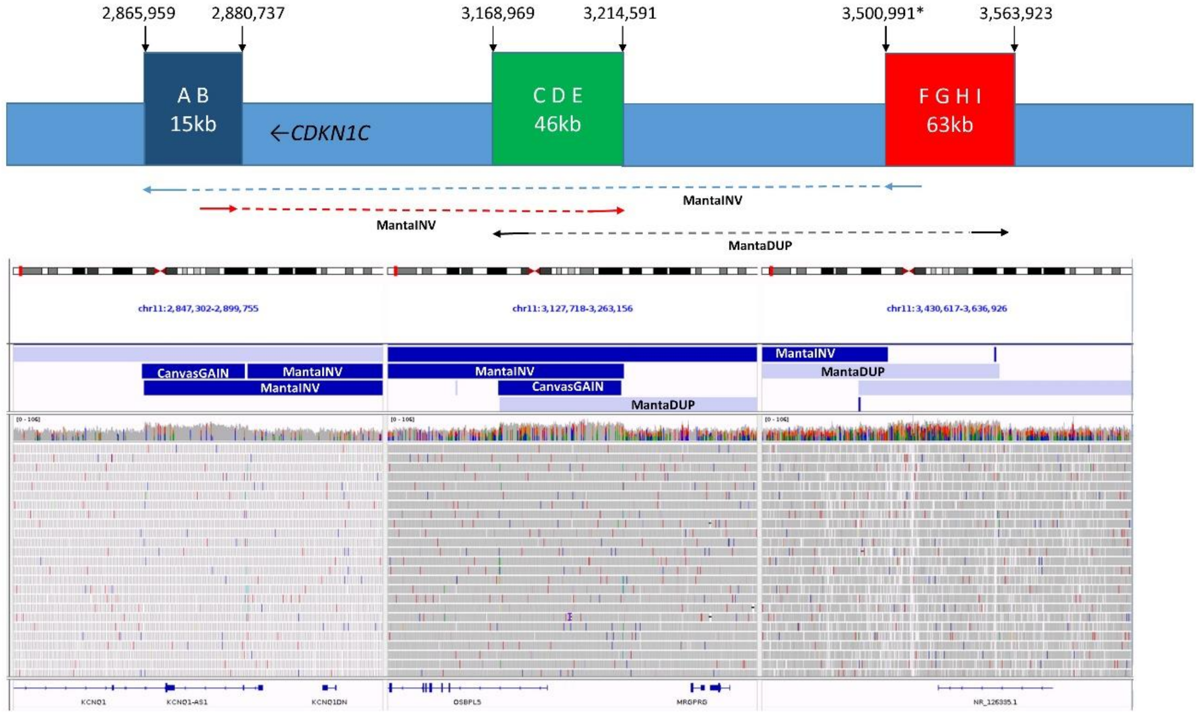

Figure S7: Summary of split reads for complex *de novo* SV found in Family 44. The three duplications on chromosome 11p15.4 are interlinked and this resulted in 2 MantaINV and 1 MantaDUP call. Two of the three duplications were called by CANVAS but the largest ~63kb was missed due to the presence of a SegDUP and low mapping quality. Positions shown are based on GRCh37.

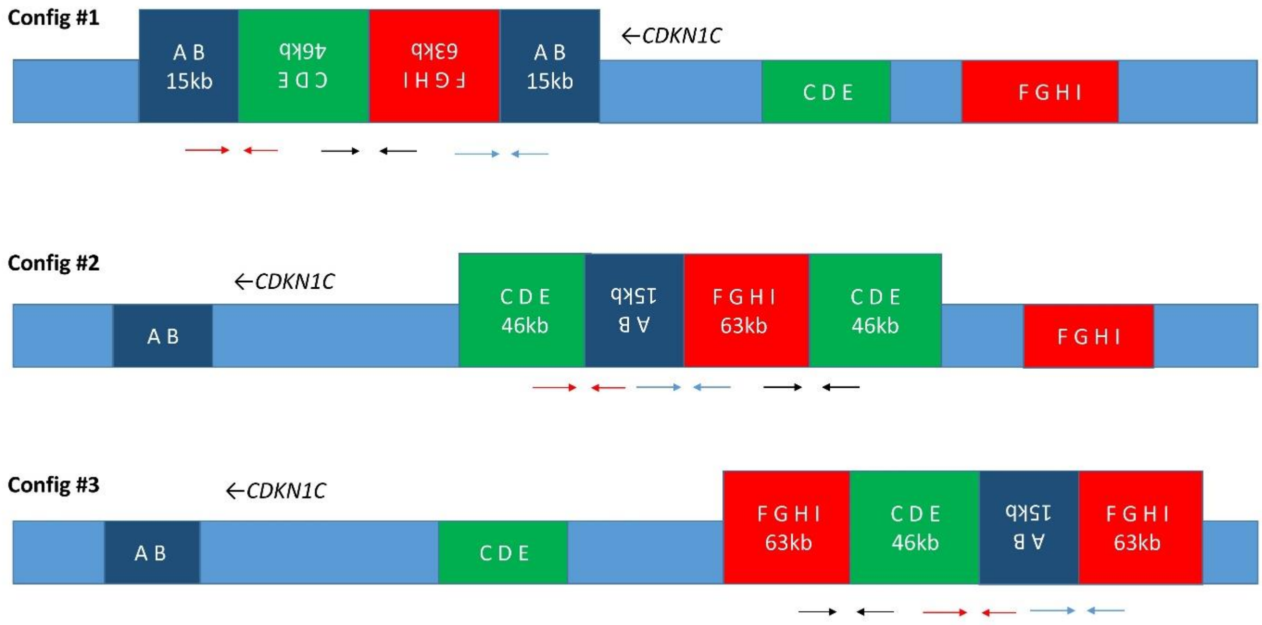

Figure S8: Schematic diagram of complex *de novo* SV found in Family 44. Short read data is ambiguous as there are three possible SV configurations that could potentially explain the split-read data. Approximate segment sizes are indicated, but not drawn to scale.

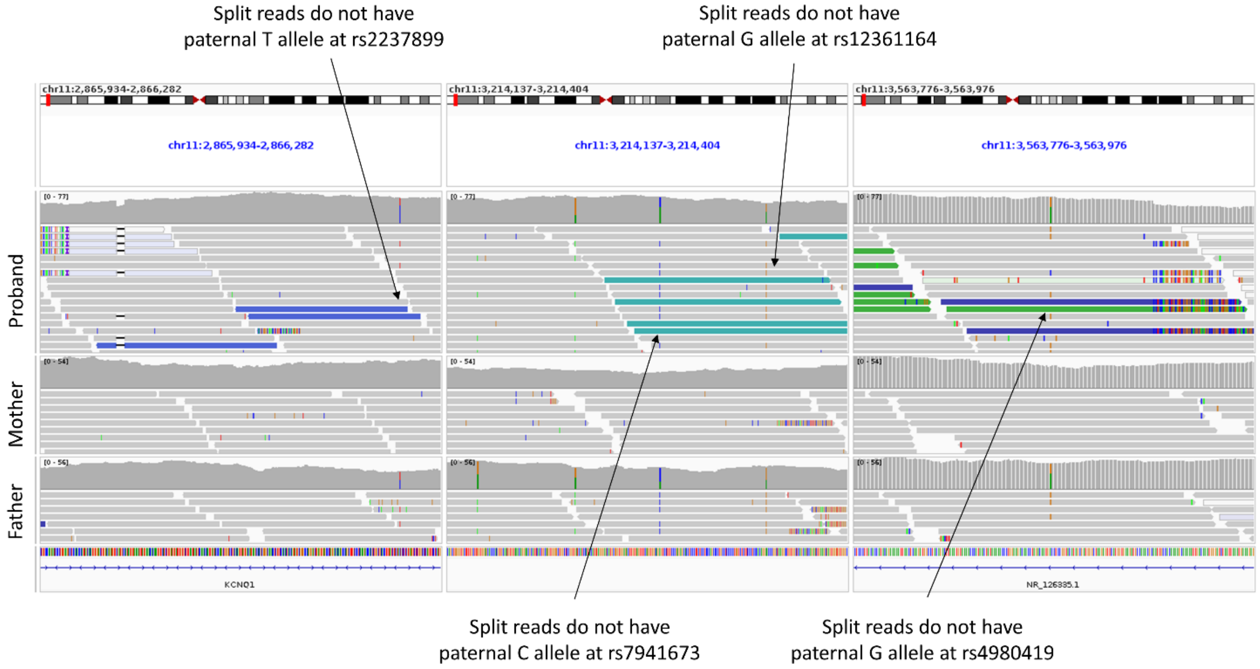

Figure S9: Informative SNPs close to the breakpoints allow phasing of the *de novo* SV found in Family 44 to the maternal chromosome. IGV screenshot shows split view corresponding to GRCh37 chr11:2865934-2866282 (rs2237899), chr11:3214137-3214404 (rs7941673, rs12361164) and chr11:3563776-3563976 (rs4980419). In each case the transmitted paternal non-reference allele is not present in read-pairs that span the SV breakpoints.

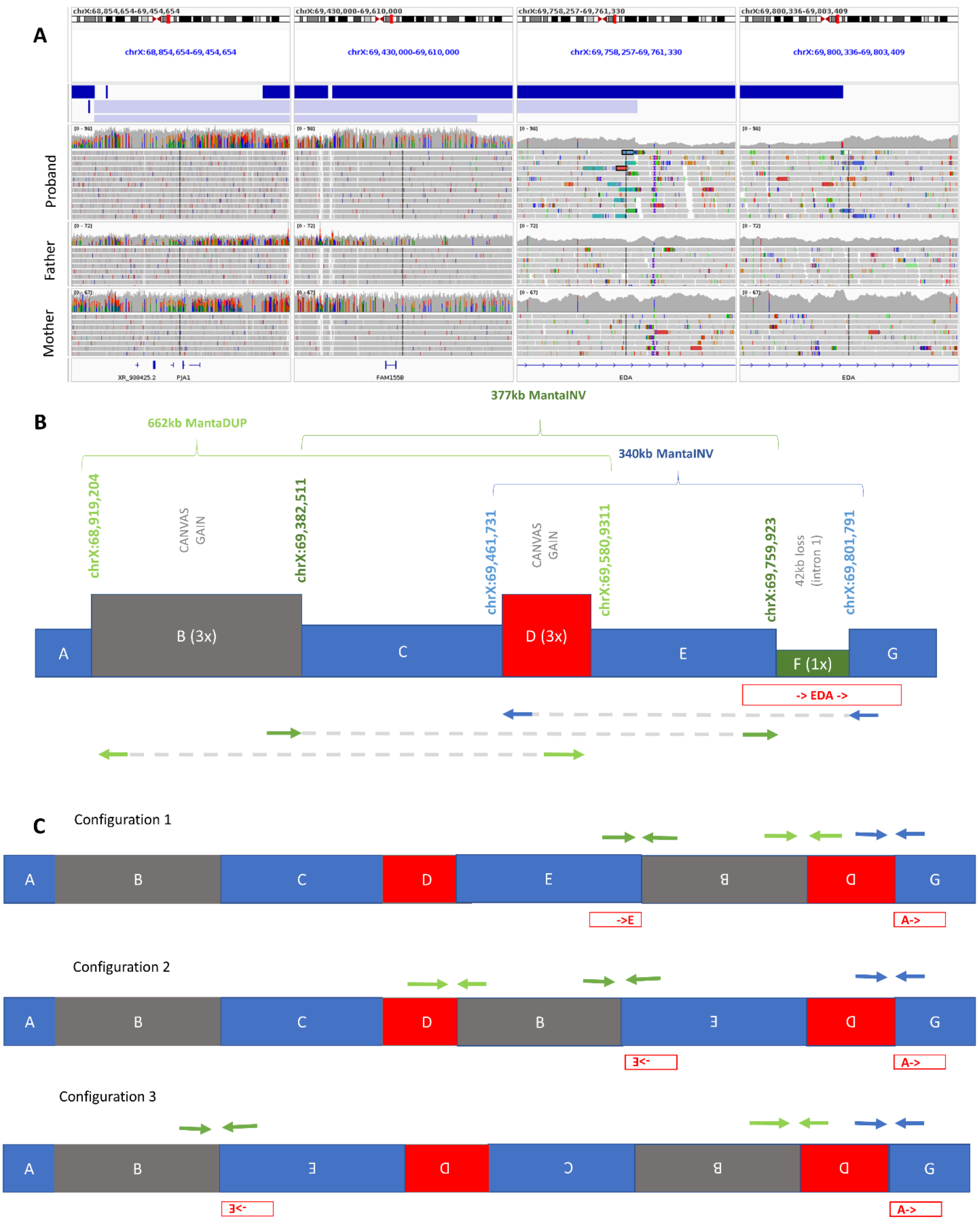

Figure S10: Read alignments and schematic diagram of a *de novo* SV involving *EDA* in Family 30. A) Read alignments shown in IGV highlighting (from LH to RHS); a 463kb gain, a 119kb gain and proximal/distal breakpoints of a 42kb deletion in intron 1 of *EDA*. The MantaINV calls (upper track) indicate that the duplicated segments are non-tandem and have been integrated at the position of the deletion. Genomic windows shown are chrX:68854654-69454654, chrX:69430000-69610000, chrX:69758257-69761330 and chrX:69800336-69803409. B) Schematic diagram (not to scale)

highlighting the duplicated segments of 463kb (grey) and 119kb (red), the relative orientation of the split reads and the resulting Manta SV calls. C) Three possible configurations can explain the short-read data as shown. For configurations #2 and #3, exon 1 of *EDA* has switched to the negative strand. However, even if configuration #1 is correct, insertion of 583kb and deletion of 42kb in intron 1 is likely to impact on correct splicing of the gene.

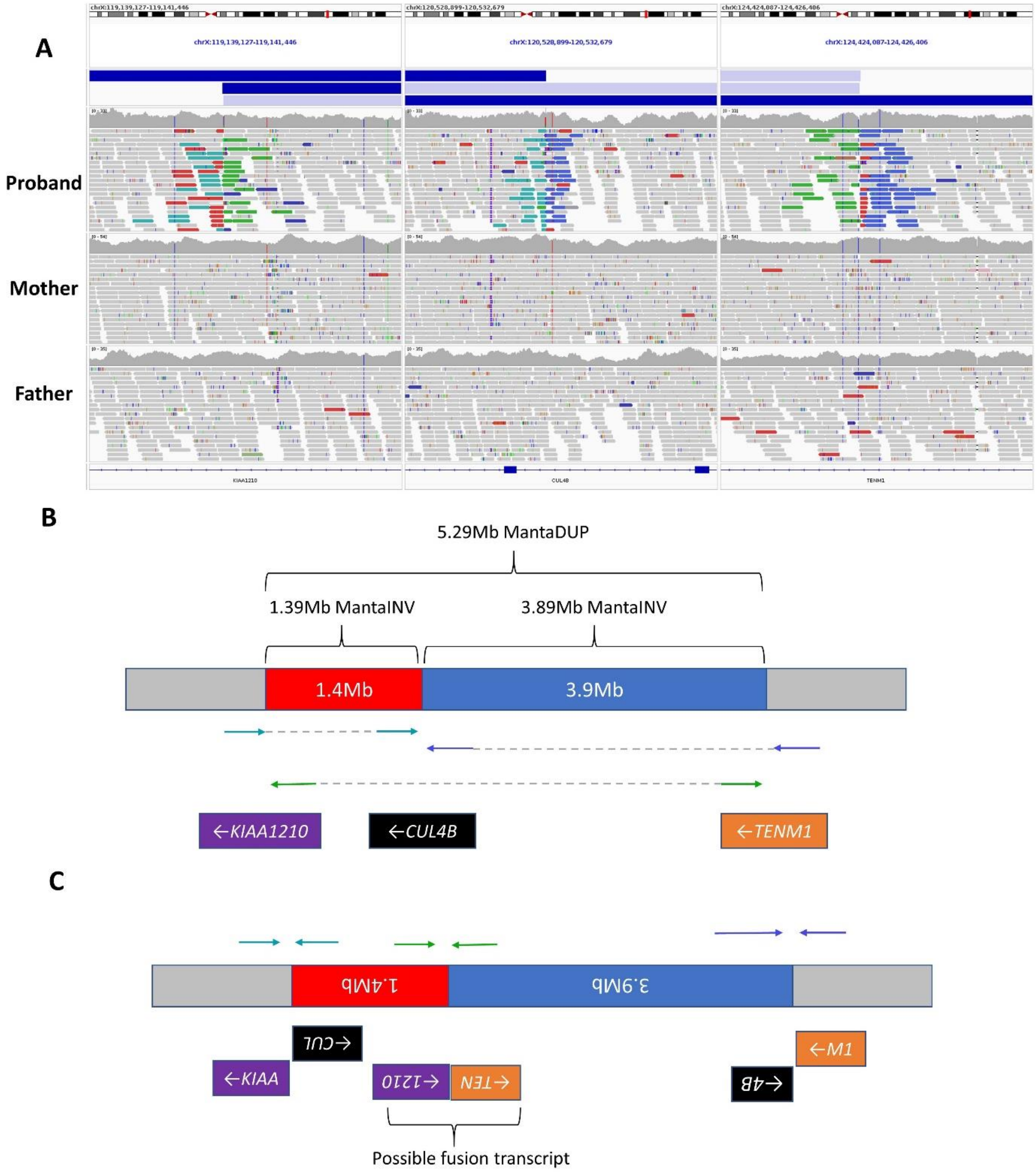

Figure S11: Complex *de novo* rearrangement in Family 39 that disrupts *CUL4B*. A) IGV screenshot showing alignments supporting two immediately adjacent inversions. Regions shown in the multi-region view are chrX:119,139,127-119,141,446 chrX:120,528,899-120,532,679 chrX:124,424,087-124,426,406 (GRCh38). B) Schematic diagram showing the relative split-read positions and Manta SVs compared to the reference genome and C) the configuration in the patient genome that can explain these pattern of split-reads. Although the breakpoints in intron 26 of 34 for *TENM1*

(NM_001163278.2) and intron 2 of 13 for *KIAA1210* (NM_020721.1) suggest the possibility of a fusion transcript involving *KIAA1210* and *TENM1*, the *TENM1* segment would be out of frame and therefore a gain of function mechanism seems unlikely.

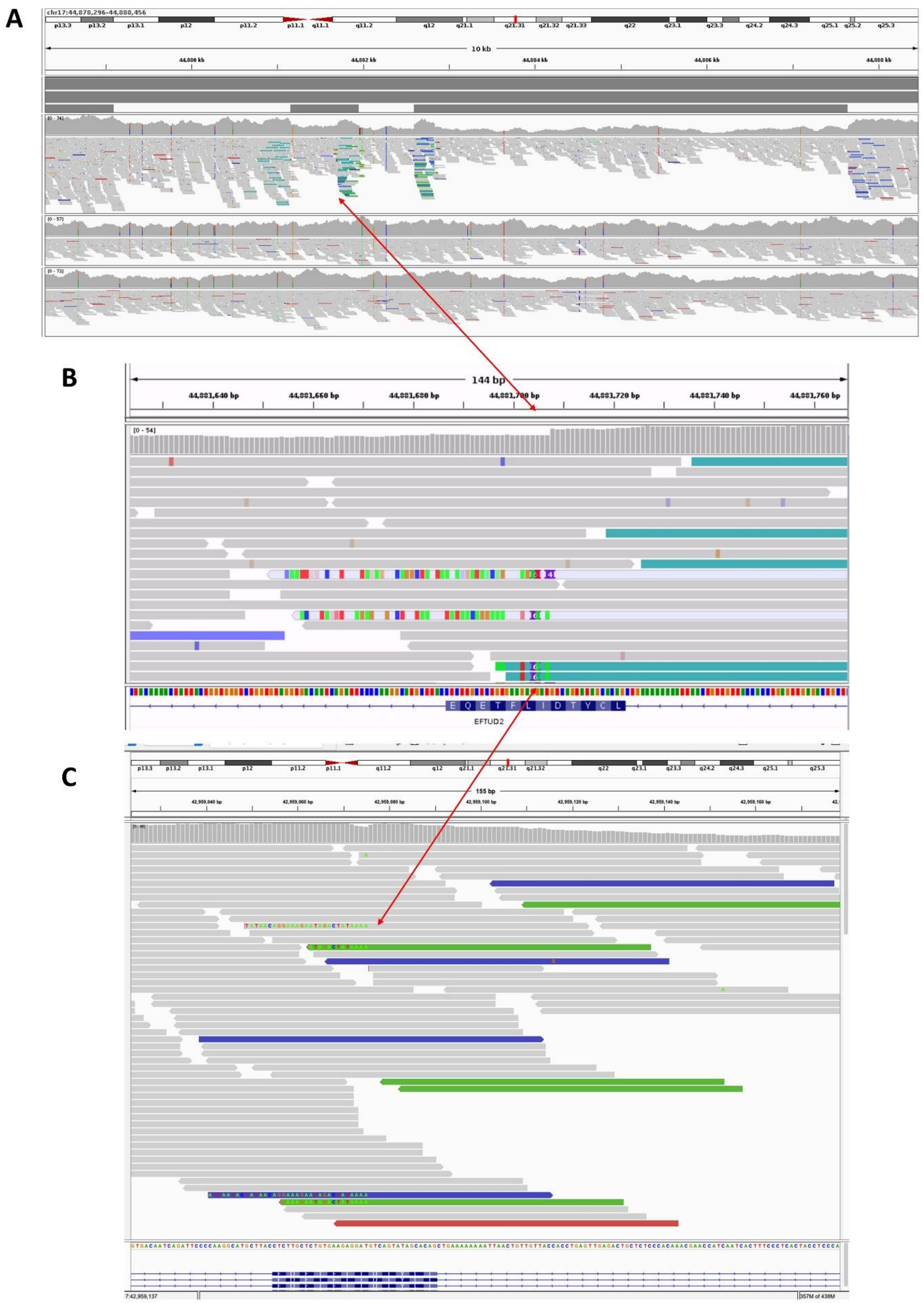

Figure S12: IGV screenshot showing read alignments supporting a complex SV involving *EFTUD2.* A) Zoomed out view showing drops in coverage and split read-pairs in proband (upper) but not in father

(middle) or mother (lower) suggesting *de novo* occurrence. The deletion contains 2 internal segments which are retained in an inverted orientation. Zoomed in view of B) genome sequence data and C) exome sequence data showing the same breakpoint in the middle of exon 7. Coordinates of the exome data are on GRCh37.

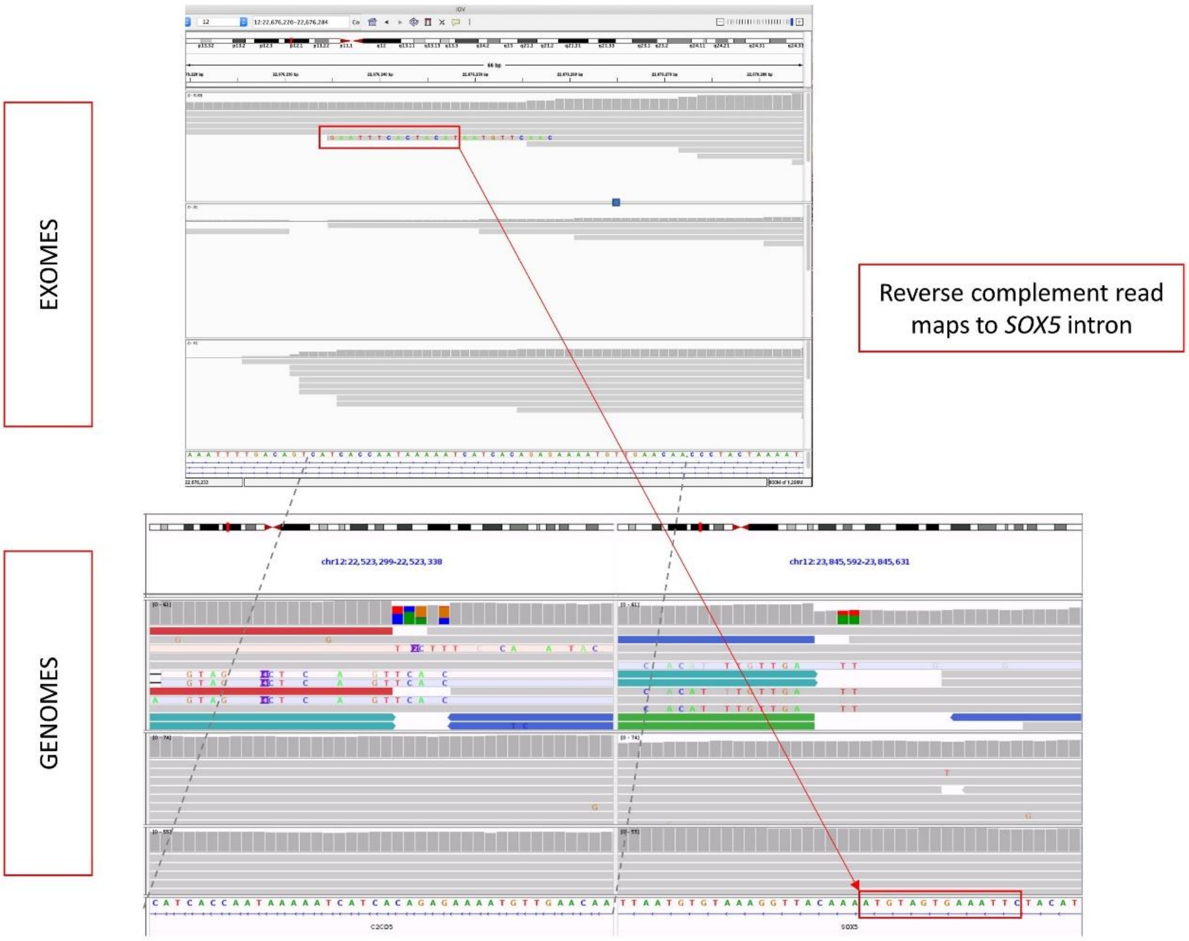

Figure S13: IGV screenshots showing read alignments supporting a *de novo* inversion disrupting *SOX5.* Top image shows trio exome data where a 1/6 reads from the proband (upper) has soft clipped sequence which maps 1.3Mb away to intron 3 of *SOX5,* but in an inverted orientation.

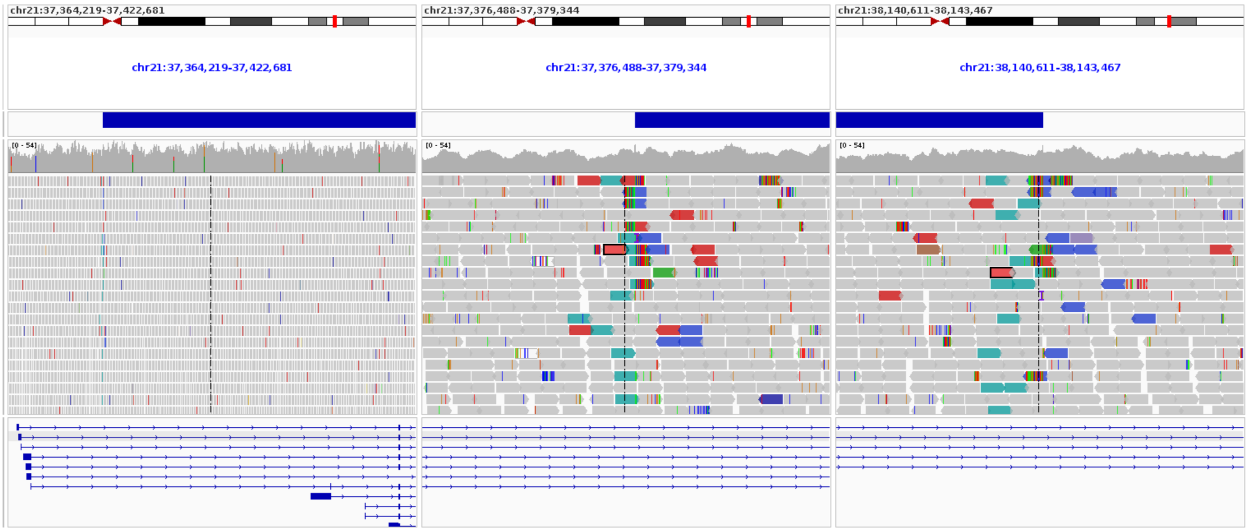

Figure S14: Read alignments and Manta calls supporting 764kb inversion that disrupts the 5'-UTR region of *DYRK1A*. The zoomed-out view (left panel) shows that, based on the canonical isoform (NM_001347721.2) and the majority of other RefSeq annotations, the proximal breakpoint lies in intron 1, whilst the start codon is in exon 2. Zoomed in views are shown of the proximal (centre panel) and distal breakpoints (right panel), the latter which lies in *DSCR8*. GRCh38 coordinates for the three IGV windows shown are chr21:37,364,219-37,422,681, chr21:37,376,488-37,379,344 and chr21:38,140, 611-38,143,467.

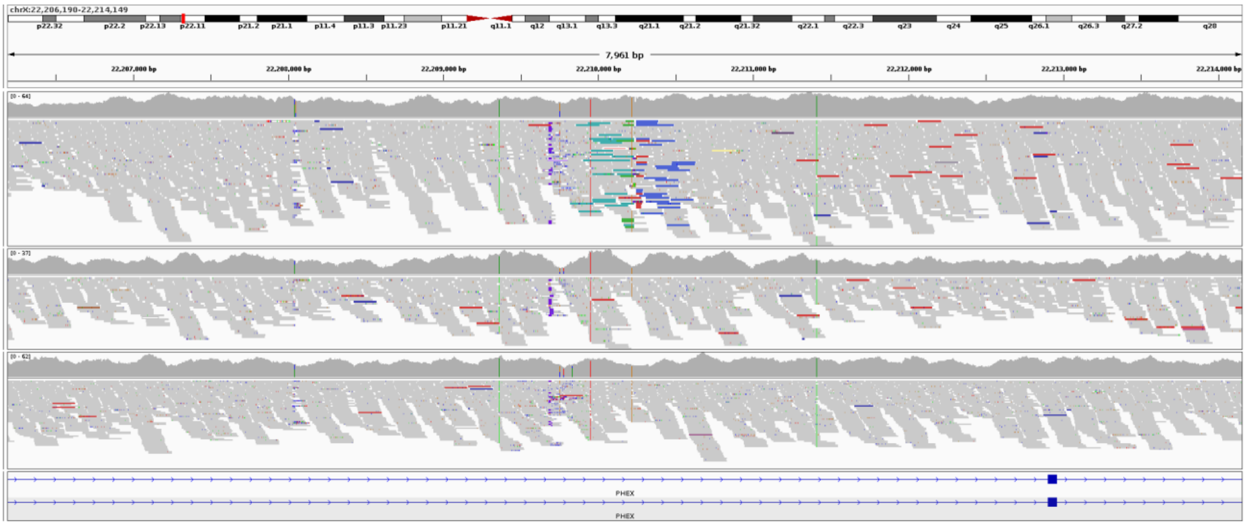

Figure S15: Read alignments supporting a 2.6Mb inversion involving *PHEX* (NM_000444.6). Only the proximal breakpoint in intron 15 is shown. This SV was identified in an individual with suspected hypophosphatemic rickets (Family 26). The absence of split read-pairs in the father (middle track) and mother (bottom) suggest that the inversion arose *de novo*.

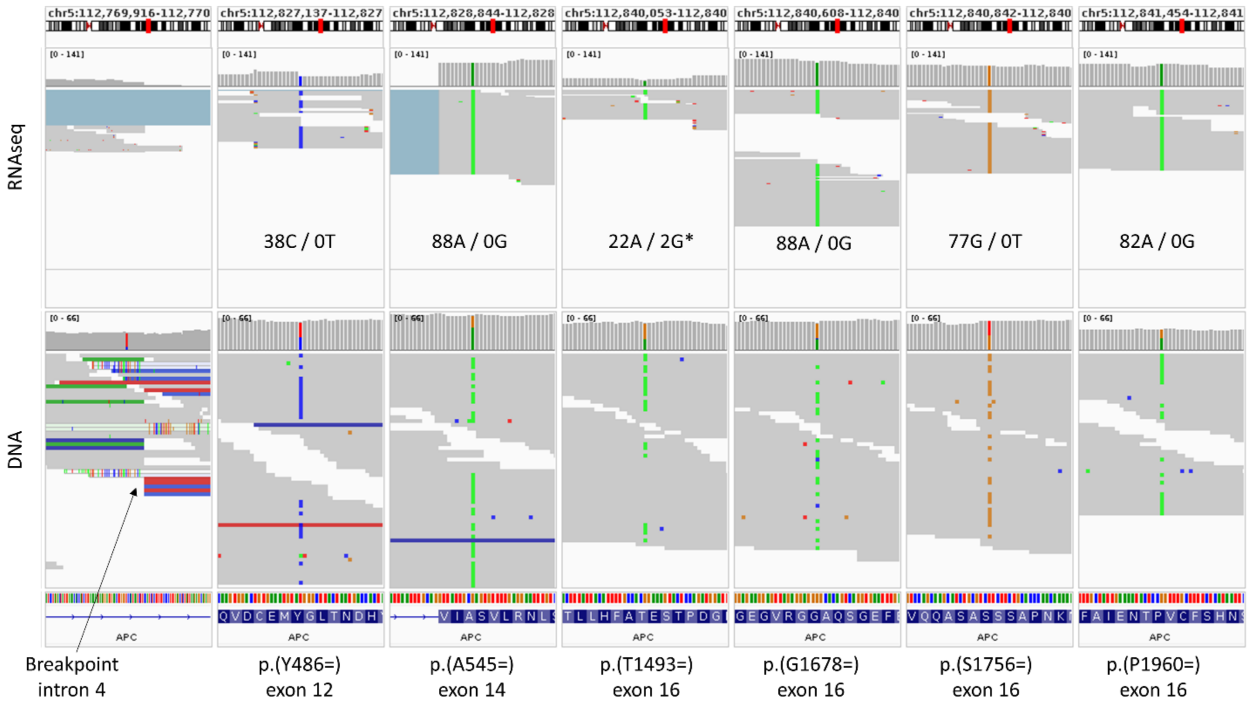

Figure S16: RNAseq data indicates that the complex *APC* translocation in Family 43 results in monoallelic expression. RNAseq data for the proband (F43-II-2; upper track) is compared to the genome sequencing data (lower) for the same individual. Monoallelic expression is apparent for a common 6 SNP haplotype (rs2229992-rs351771-rs41115-rs42427-rs866006-rs465899; C-A-A-A-G-A) spanning exons 12-16 (NM_000038.6). Only the non-reference alleles were expressed. PacBio data (available for F43-I-1) confirmed that the SV lies *in cis* with the reference alleles at these respective sites. *both Gs lie at the ends of reads.

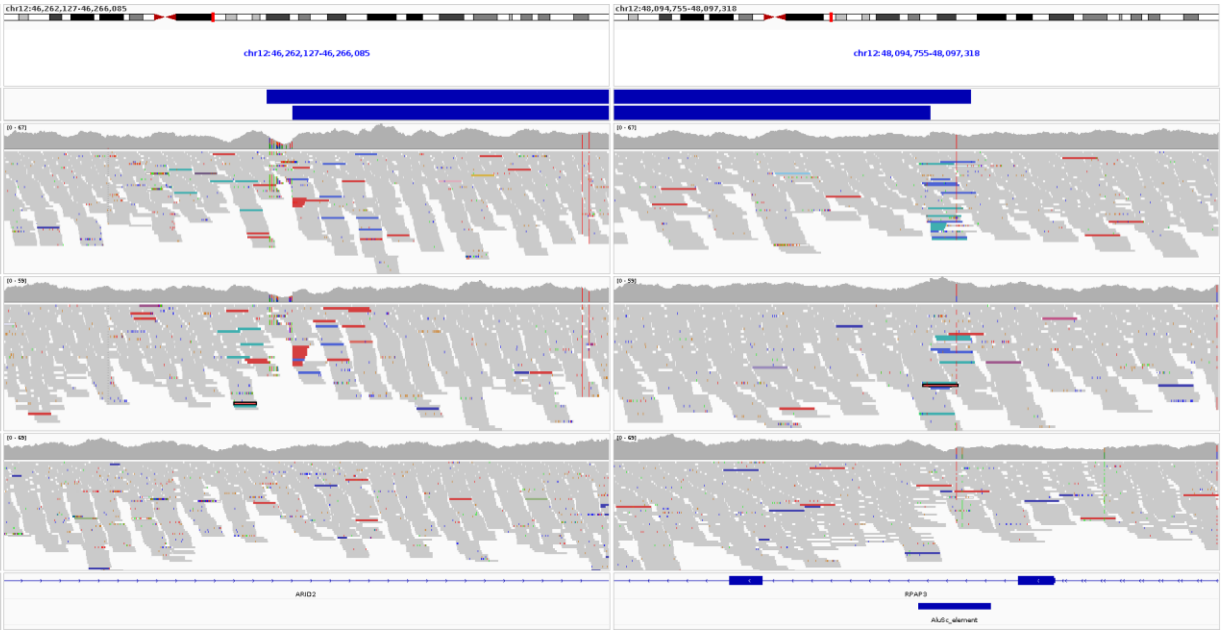

Figure S17: Read alignments and Manta calls suggesting possibility of a 1.8Mb inversion disrupting *ARID2* in Family 35 in proband (top) and inherited from the mother (middle). The bottom track is a control genome sequenced in the same batch as the mother. Although the +ve strand split read pairs (green) and the -ve strand split read pairs (blue) lie distinctly at each side of the breakpoint on the

proximal side, at the distal end they overlap and coincide with an intronic AluSc element. A more likely explanation of this data is therefore an intronic retrotransposon event into *ARID2* intron 16.

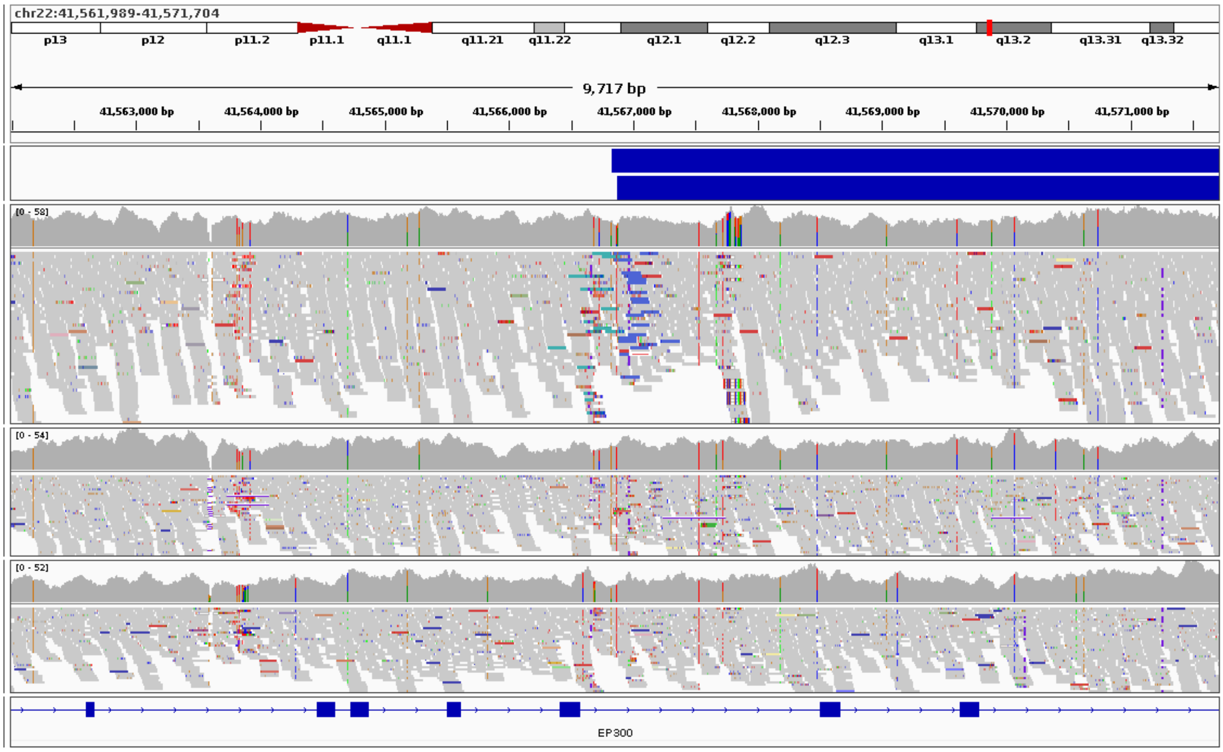

Figure S18: IGV screenshot showing read alignments supporting the 1.0Mb inversion on 22q13.2 in Family 18 that disrupts *EP300* and *TCF20*. In this view, only the proximal breakpoint that disrupts *EP300* in intron 27/30 (NM_001429.4) is shown. The horizontal blue bars in the top track show the reciprocal MantaINV calls. The parental data is shown in the two tracks immediately below that of the proband, confirming the SV to have arisen *de novo*.

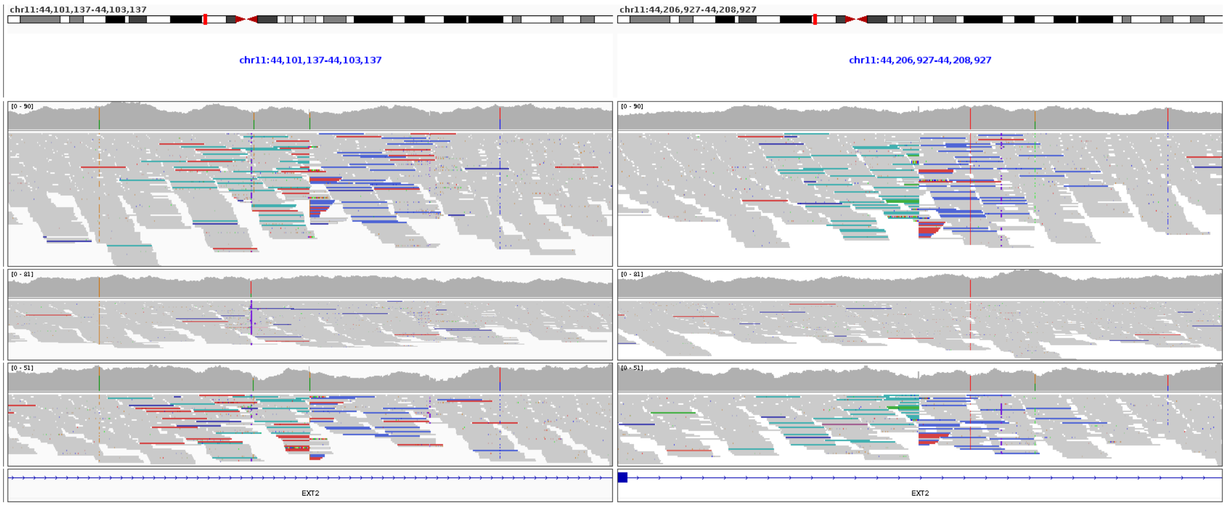

Figure S19: IGV screenshot showing read alignments supporting the 106kb inversion disrupting *EXT2* in Family 4. The inversion is seen in the proband (upper) and is inherited from the father (bottom) but not seen in the mother's data (middle). The reads are shown using the split-window option so that both the proximal and distal breakpoints can be viewed at the same time. The structural rearrangement inverts exons 2-10 of the 14 exon gene.

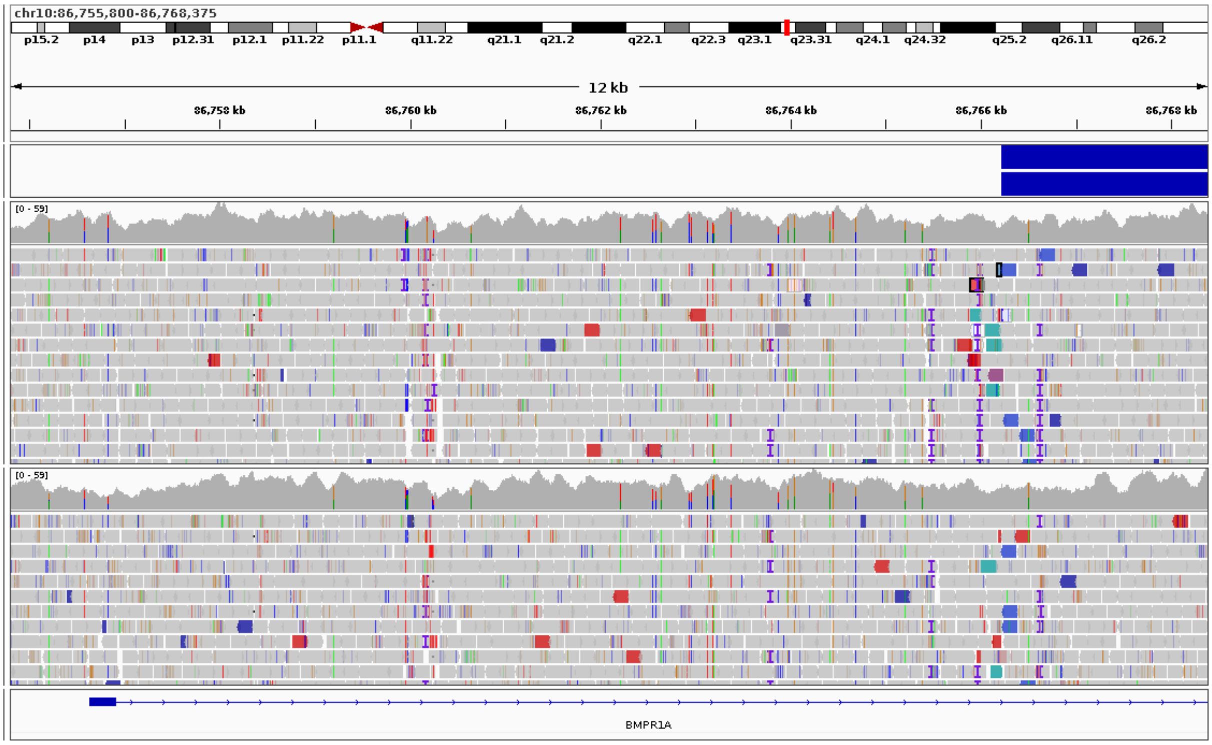

Figure S20: Read alignments and reciprocal MantaINV calls for a 1.4Mb inversion with a proximal breakpoint in intron 1 of *BMPR1A*. The inversion is seen in both the proband in Family 1 (upper) and in her affected mother (lower); both these individuals have a phenotype consistent with classical juvenile polyposis syndrome.

A

B

C

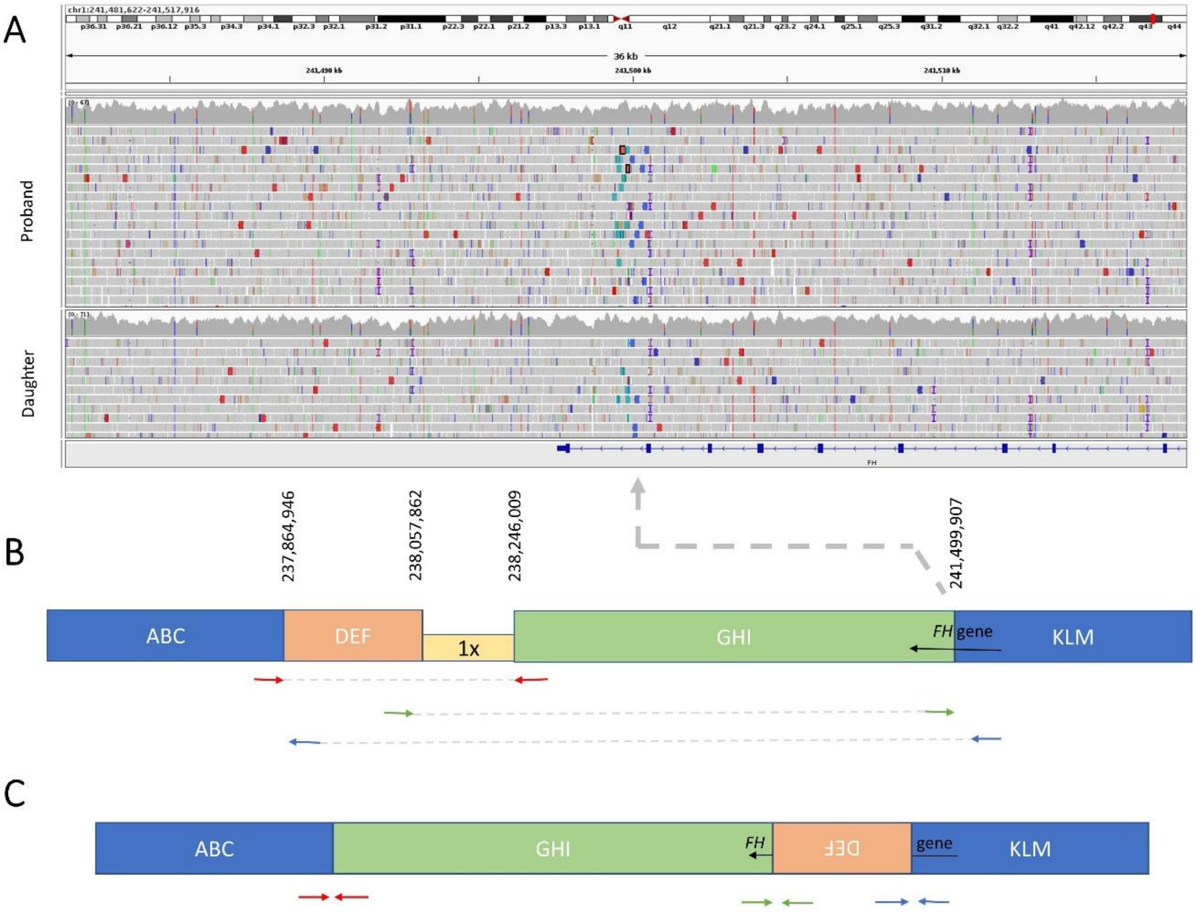

Figure S21: Read alignments and schematic diagrams explaining the structure of a complex rearrangement disrupting the fumarate hydratase gene. A) IGV screenshot showing split read pairs highlighted in green/blue that signify a breakpoint in the final intron of *FH*. B) Diagram summarising the positions of the split read-pairs. Plus to plus strand mappings are shown in red arrows and minus to minus strand mappings are shown as green arrows. Chromosomal segments are labelled A-M to help with orientation. Although mostly balanced, the rearrangement also involves a deleted segment (yellow) of 188kb in size. C) Schematic diagram showing the structure of patient genome that explains the split read-pairs in panel B. Segments are not shown to scale and genomic coordinates are based on GRCh38.

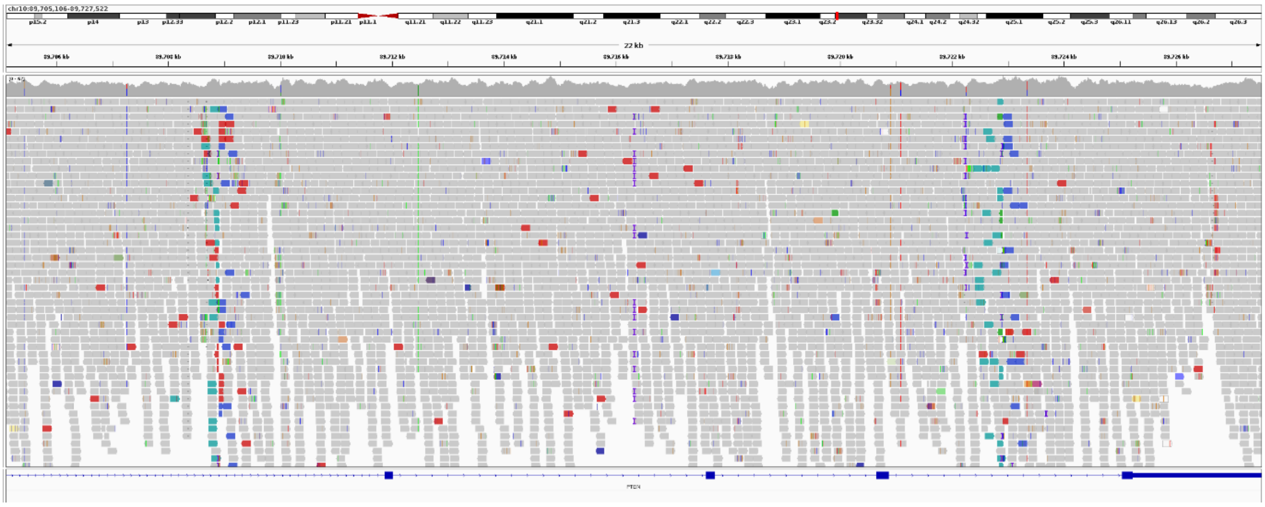

Figure S22: Read alignments supporting a 14kb inversion in Family 6 that disrupts *PTEN*. The rearrangement involves exons 6-8 and so is highly likely to disrupt gene function. Although the data shown here is on GRCh37, the coordinates were lifted over to GRCh38 for the purposes of SVRare and for Tables 1 and S2.

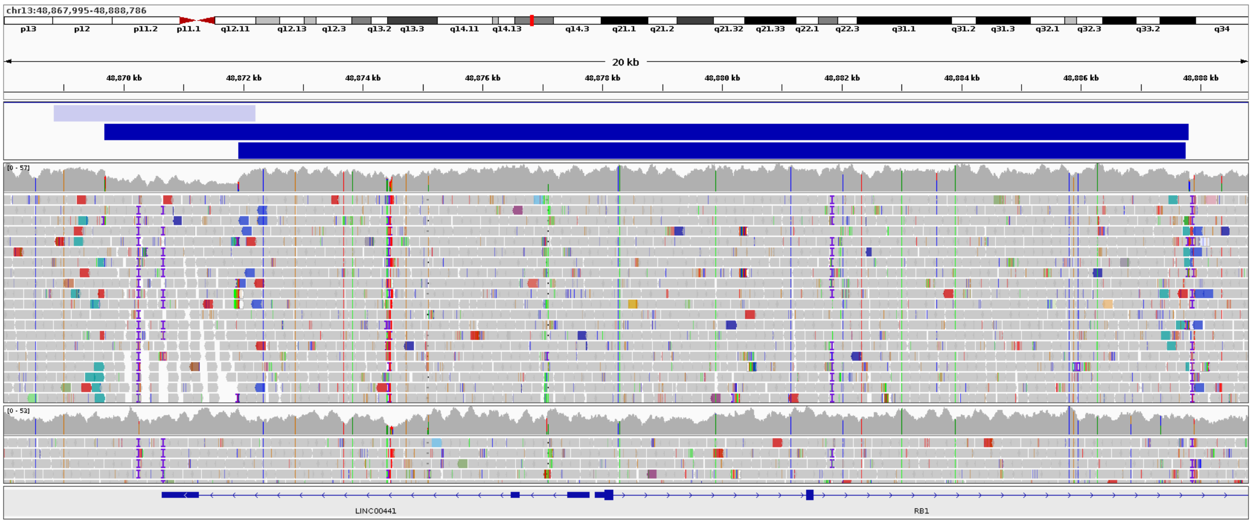

Figure S23: Read alignments supporting an 18.1kb inversion in Family 8 that disrupts *RB1*. The variant is private to this family amongst data from the 100kGP and as the distal breakpoint lies in intron 2, it is highly likely to disrupt gene function. The proximal end of the inversion is associated with a 2246bp loss involving the last exon of *LINC00441.* Identification of the loss only would have prioritised the wrong gene. The top track shows the Canvas call overestimates the deleted region (light blue), whilst the Manta accurately detected both reciprocal breakpoints of the inversion (dark blue). The middle track shows read alignments from the patient and the bottom track shows data from a control individual. The data is shown on GRCh37 build but we note coordinates have been lifted over to GRCh38 in Table S2.

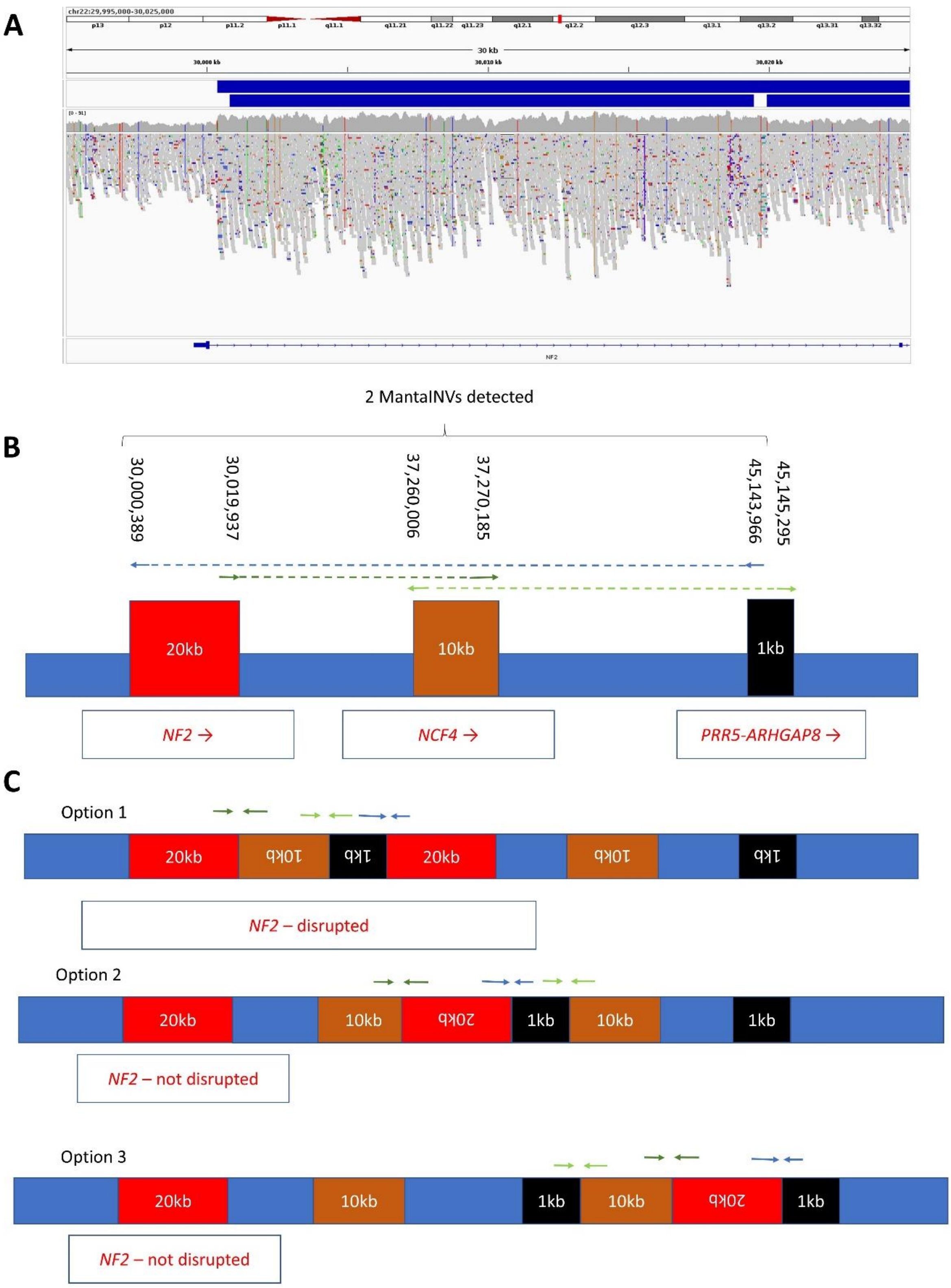

Figure S24: Complex SV involving *NF2* in Family 40. A) Read alignments shown in IGV are for the larger of the 3 duplicated segments which lies in intron 1 of *NF2*. B) Schematic diagram showing the 3 interlinked duplicated segments and the relative positions of the split read-pairs that result in the MantaINV call. *PRR5-ARHGAP8* refers to a readthrough transcript NM_181334.6. Genome coordinates are based on GRCh37 but the coordinates are converted to GRCh38 for Table S2. C) Schematic diagram showing 3 possible solutions to the short-read data. Only the first of these configurations is likely to

disrupt *NF2* and even then the duplication/insertion lies in intron 1. Clinical presentation and initial analysis of long-read PacBio sequencing data do not support options 2 and 3.

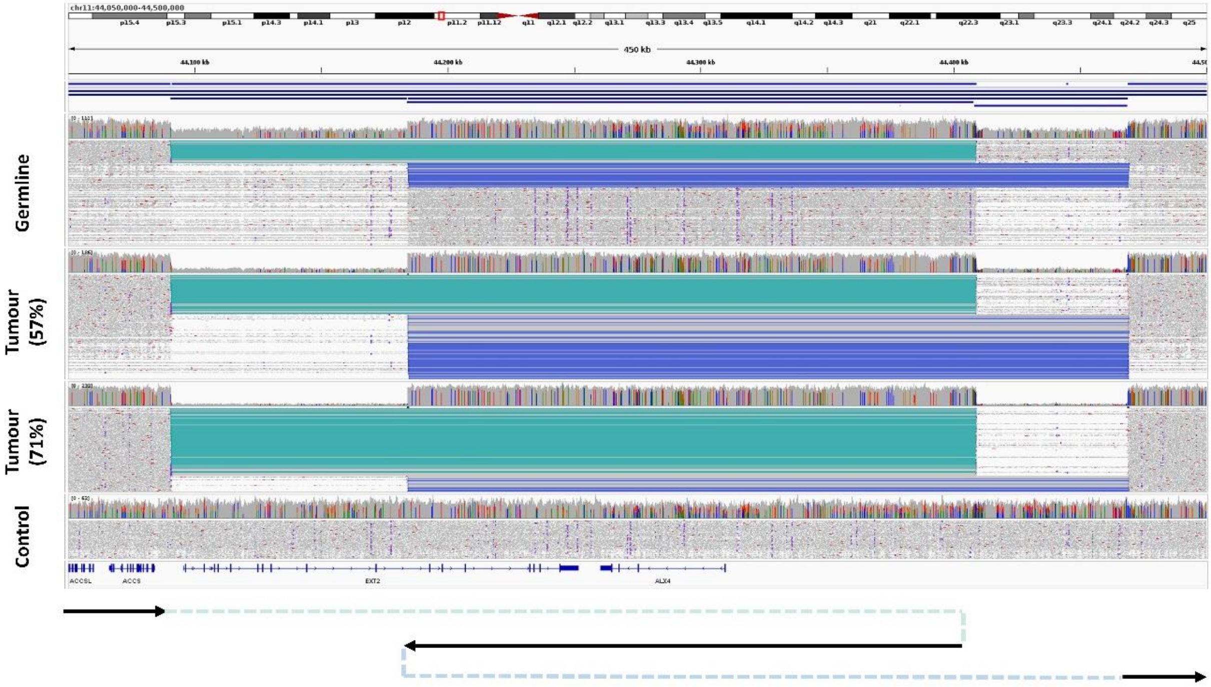

Figure S25: Example of a germline deletion-inversion from the cancer arm of the 100kGP that removes exons 1-8 of *EXT2* (NM_207122.2). The rearrangement was seen at a far higher allelic fraction in the tumour samples (of differing purity) due to a somatic chromosome 11 cnLOH event and so is predicted to result in a complete loss of *EXT2*. Multiple biopsies, all from the primary tumour of a participant with multiple exostoses and chondrosarcoma were sequenced alongside the germline. The +ve to +ve strand mapping read-pairs (teal) and the -ve to -ve mapping pairs (blue) indicates that the central segment has been inverted, as shown in the schematic diagram below. Reads are shown as pairs, squished and sorted by insert size. Genome coordinates are on GRCh38. The track at the top shows squished Manta/CANVAS calls. The control sample is a randomly selected sample sequenced in the same batch as the germline sample.

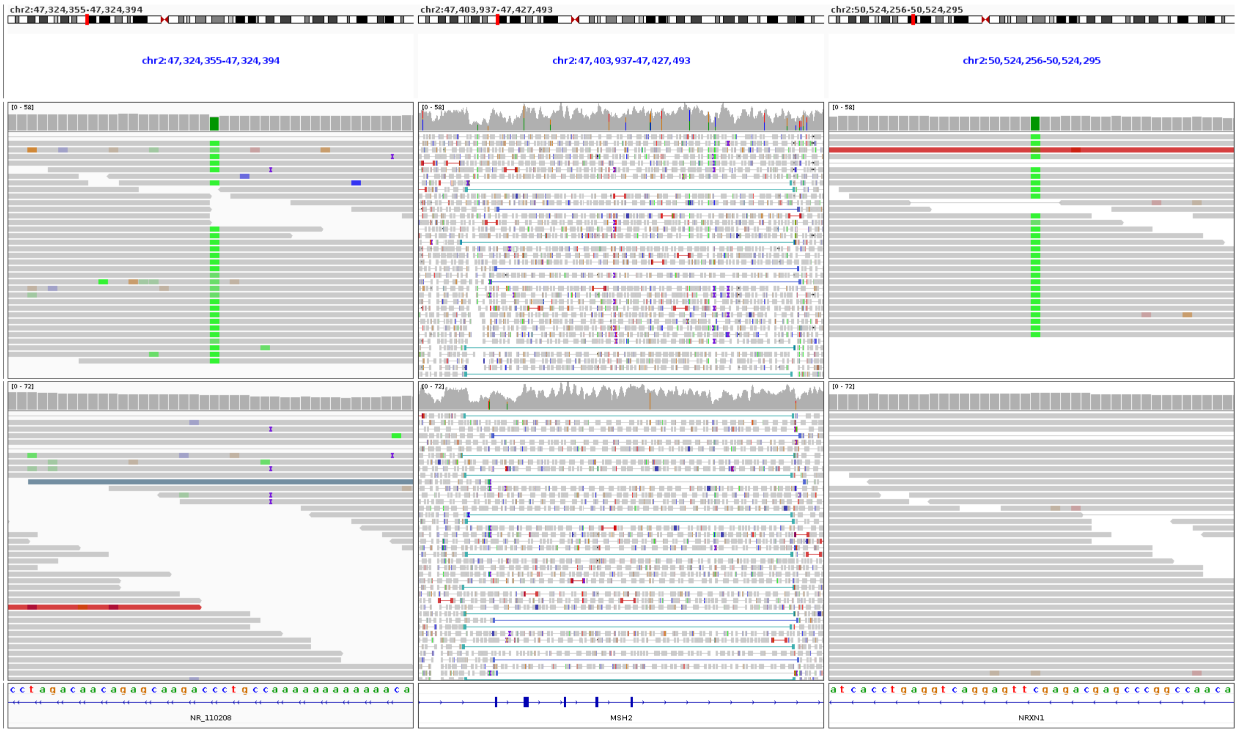

Figure S26: Conflicting homozygosity at common SNPs that flank the *MSH2* inversion haplotype and define the maximal shared region to be 3.2Mb (chr2:47,324,375-50,524,276, GRCh38). IGV image shows data for F16 (upper) and F17 (lower). The three windows show read alignments supporting rs115321698 (left), the inversion (middle) and rs13420048 (right).

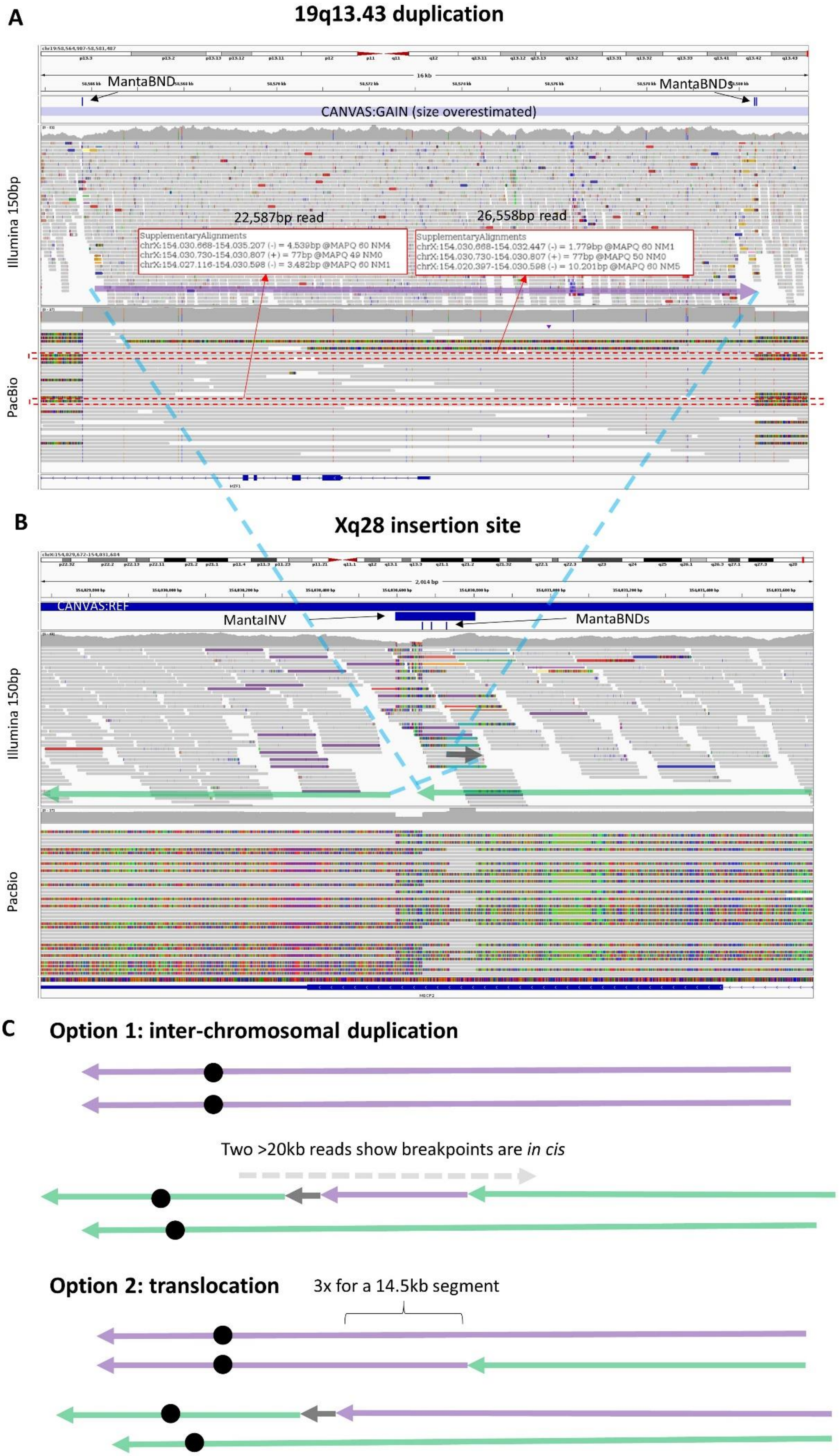
A
19q13.43 duplication
MantaBND
MantaBNDs
CANVAS:GAIN (size overestimated)
Illumina 150bp
22,587bp read
SupplementaryAlignments
chrX:154.030.668-154.035.207 (-) = 4.539bp @MAPQ 60 NM4
chrX:154.030.730-154.030.807 (+) = 77bp @MAPQ 49 NM0
chrX:154.027.116-154.030.598 (-) = 3.482bp @MAPQ 60 NM1
26,558bp read
SupplementaryAlignments
chrX:154.030.668-154.032.447 (-) = 1.779bp @MAPQ 60 NM1
chrX:154.030.730-154.030.807 (+) = 77bp @MAPQ 50 NM0
chrX:154.020.397-154.030.598 (-) = 10.201bp @MAPQ 60 NM5
PacBio
B
Xq28 insertion site
CANVAS:REF
MantaINV
MantaBNDs
Illumina 150bp
PacBio
C
Option 1: inter-chromosomal duplication
Two >20kb reads show breakpoints are in cis
Option 2: translocation
3x for a 14.5kb segment

Figure S27: Representation of a complex inter chromosomal rearrangement disrupting the final exon of *MECP2*. A) IGV screenshot showing Manta and CANVAS calls (upper panel), Illumina 150bp read alignments (middle) and PacBio long reads (lower) for the proband in Family 47. Two PacBio reads of >20kb are highlighted that span both breakpoints and for these the information about the supplementary alignments are shown. B) IGV image similar to above for the Xq28 locus, showing *in silico* calls and read alignments supporting the complex SV. Dotted blue lines highlight the junctions between the chromosome segments. C) schematic diagram highlighting the two possible configurations that could explain the short read WGS data. Chr19 is shown in purple whilst chrX segments are in green/grey, consistent with the colour coding in panels A/B. Due to the two long reads shown in panel A the presence of two derivative chromosomes (and thus a translocation event) could be ruled out.

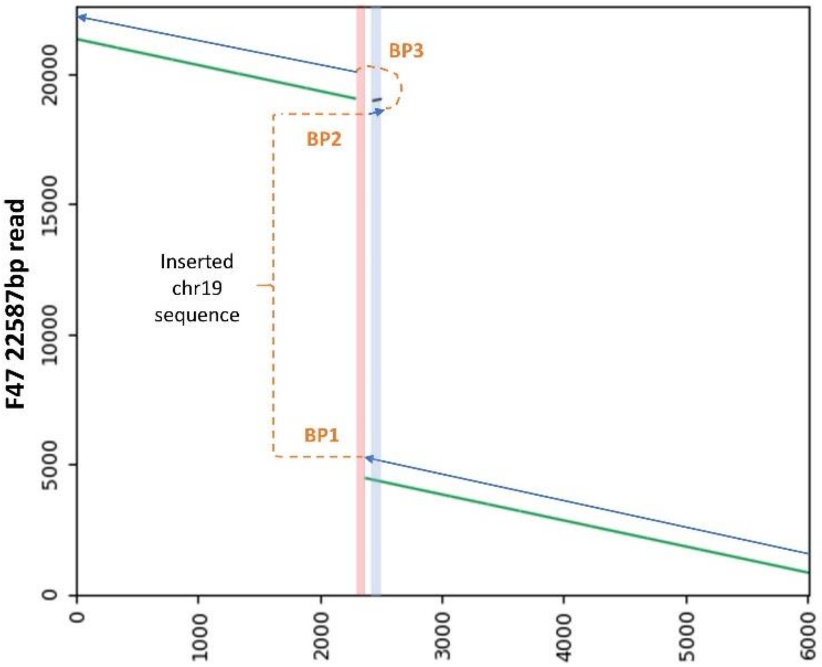

Figure S28: Dot-plot using the 22.6kb PacBio read indicated in Figure S27A showing the structure of a *de novo* inter-chromosomal duplication in Family 47 that involves the final exon of *MECP2*. To enable comparison to the *MECP2* rearrangement seen for F33, the X axis corresponds to the identical region shown in Figure 4 (chrX:154,028,301-154,034,315, GRCh38). Grey and green lines indicate sense/antisense matches to the reference, whilst the blue/orange lines help show how these segments are connected. The vertical red and blue shading highlights deleted and duplicated regions respectively.

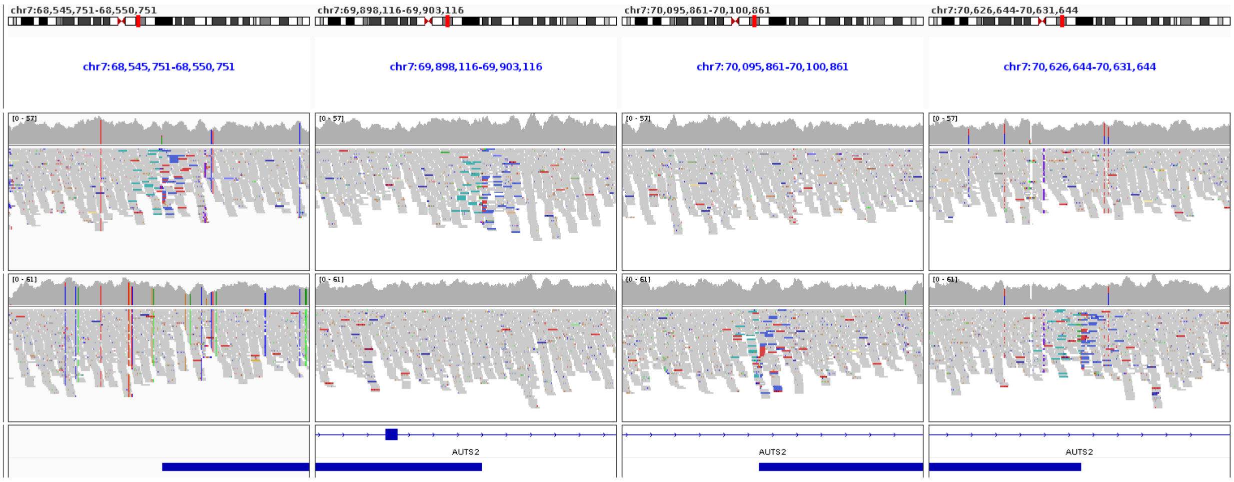

Figure S29: Proband-only read alignments and MantaINV calls for 2 *de novo* inversions involving *AUTS2* (NM_015570.4). For the 1.35Mb inversion in Family 37 (upper), the distal breakpoint lay near the start of intron 2. For the 531kb inversion in Family 38 (lower), the proximal and distal breakpoints lay towards the end of intron 2 and in the middle of intron 5, respectively. GRCh38 coordinates for the IGV windows shown are chr7:68545751-68550751, chr7:69898116-69903116, chr7:70095861-70100861 and chr7:70626644-70631644.

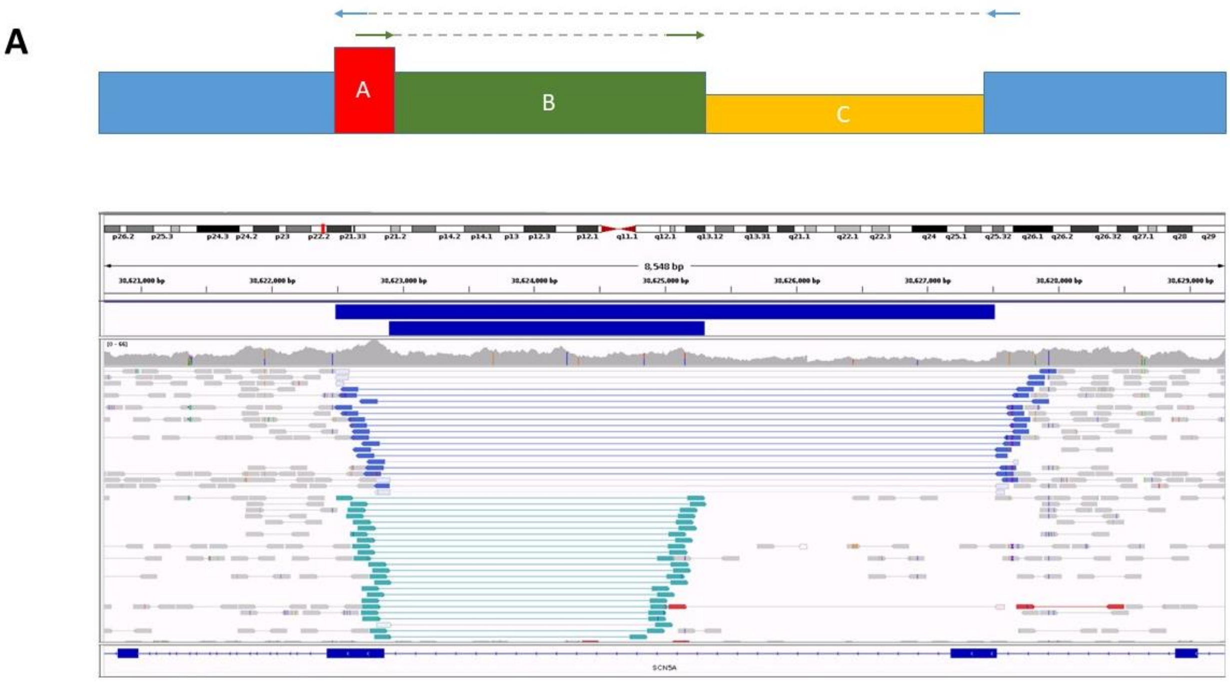

Deletes nearly all exon 16 (NM_000335.5) and non-tandem inverted duplication of nearly all exon 17

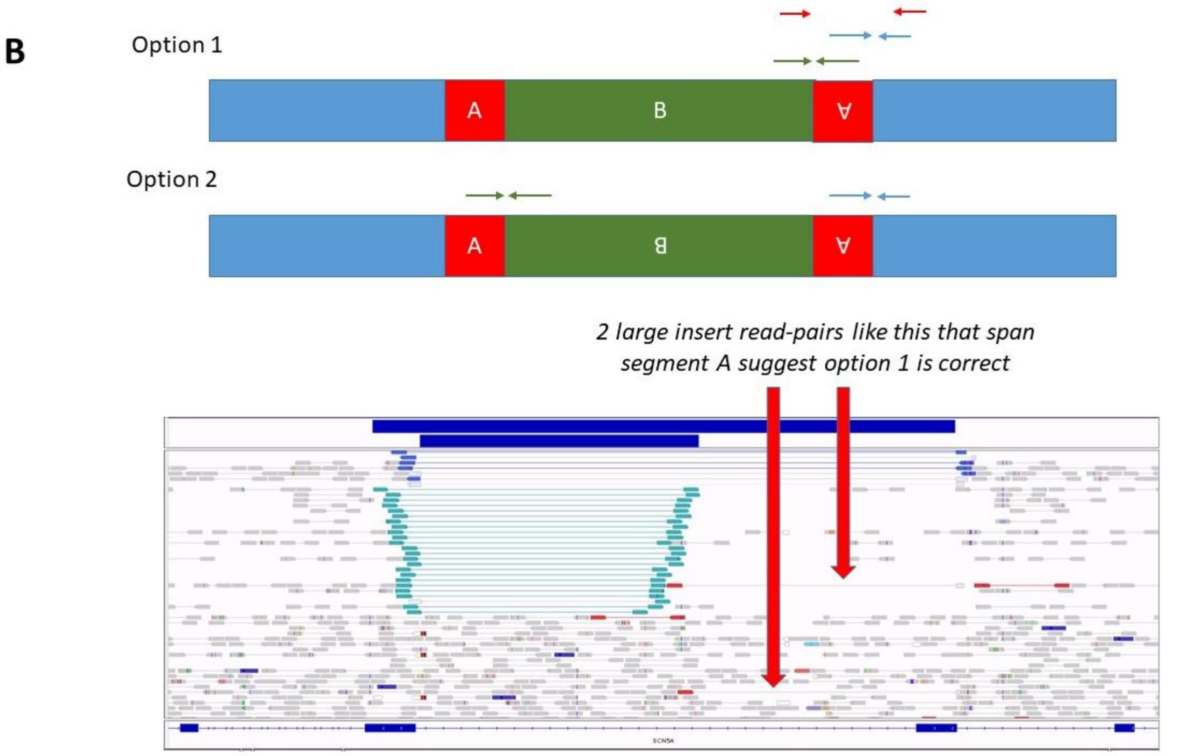

Figure S30: Schematic diagrams and read alignments supporting a *SCN5A* variant from the 100kGP pilot study. A) Schematic diagram highlights the different copy number states and split read-pairs. IGV screenshot shows read-pair alignments, which are sorted by size. B) Schematic diagram highlights two possible solutions to the short-read data. However, as there are two read pairs with large insert sizes that span the deleted region and also the 406bp segment A (red arrows), option 1 appears to be the correct orientation.

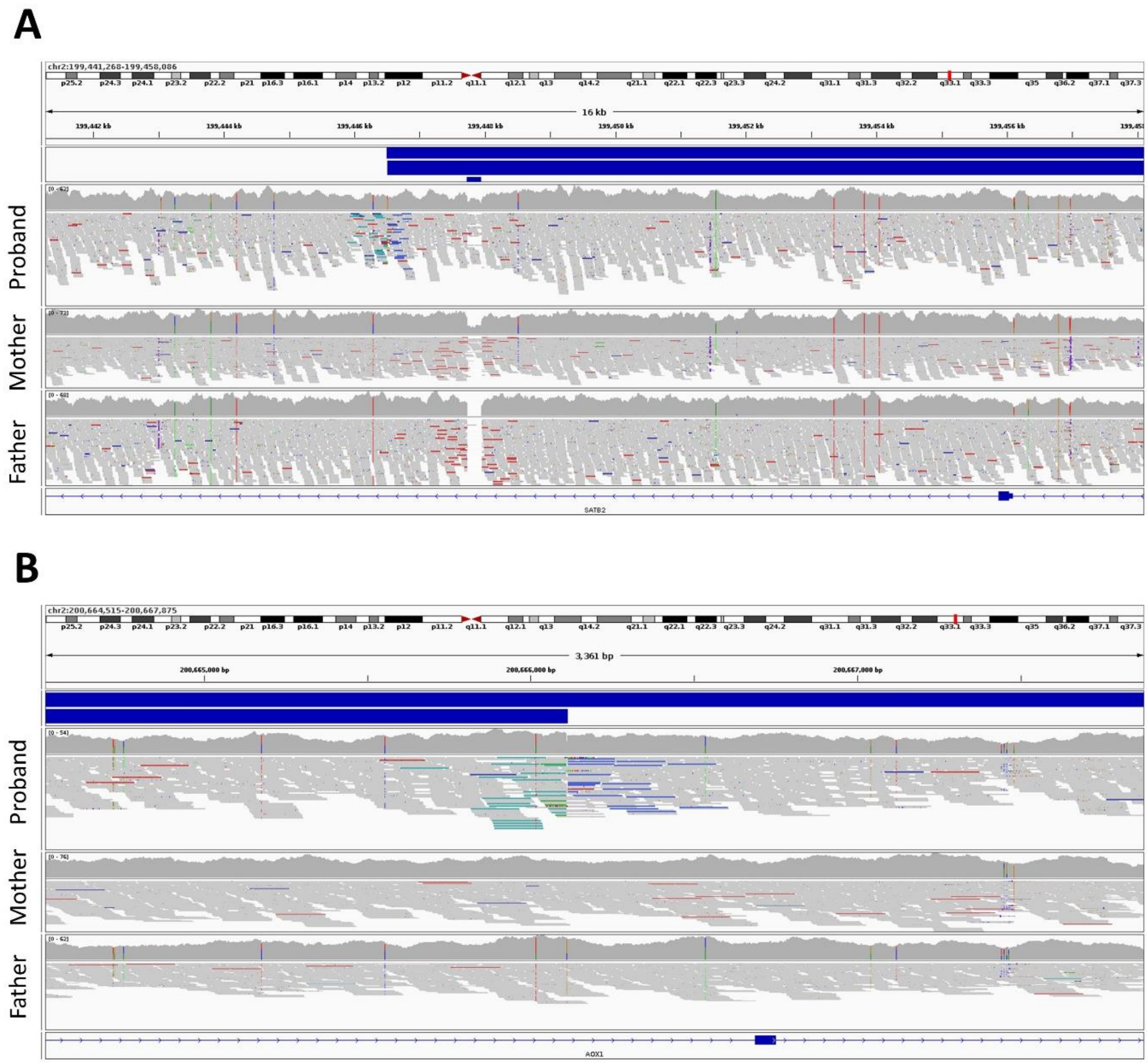

Figure S31: Read alignments supporting a *de novo* inversion involving *SATB2* (NM_015570.4) in Family 15. A) The proximal breakpoint of this 1.22Mb inversion lies in intron 2 of *SATB2,* whilst B) the distal breakpoint disrupts *AOX1*, a gene according to OMIM not known to be associated with disease.

Supplemental Tables

Table S1: Set of curated disease associated genes where haploinsufficiency is a known disease mechanism (HI Score = 3). List downloaded from ClinGen November 2022. Coordinates from initial download were from GRCh37 and so these were switched to GRCh38 using the UCSC LiftOver tool. *Table available as separate xlsx file.*

Table S2: Full details for 47 families with rare structural variants detected on account of a MantaINV call. *Table available as separate xlsx file.*

Table S3: Targeted validation strategies and PCR primers used for families where the SV has been confirmed with an orthogonal approach.

Family	Gene	Strategy (laboratory type)	Primers or other details
1	*BMPR1A*	PCR-Sanger (clinical)	BMPR1AChr10inv_P1F N13-TACCATGCCCAGCTAATTAAAAAAT<br>BMPR1AChr10inv_P1R N13-ACTGCCTAATCCGGGTGTTT<br>BMPR1AChr10inv_P2F N13-ATGGTACGGGTCGATTAATTTTTTA<br>BMPR1AChr10inv_P2R N13-TGACGGATTAGGCCCACAAA<br>BMPR1AChr10inv_D1F N13-TCAGAAAATGGAATAACTGCTTAAC<br>BMPR1AChr10inv_D1R N13-TTACCTTCATGGGATGCACA<br>BMPR1AChr10inv_D2F N13-AGTCTTTTACCTTATTGACGAATTG<br>BMPR1AChr10inv_D4R N13-AATGGAAGTACCCTACGTGT
2	*FH*	PCR-Sanger (clinical)	FH_BREAKPOINT_A_F N13-AACCCAAGGGCTGGATCAAA<br>FH_BREAKPOINT_A_R N13-ACCAAGGTTGACTTGGCCTG<br>FH_BREAKPOINT_B_F N13-CTGGGAAGAAAAAGAGGCTTA<br>FH_BREAKPOINT_B_R N13-GTTGTGGGAGAAACCTGGTG<br>FH_BREAKPOINT_C_F N13-TTAAGTGGAGGAGGCATTGG<br>FH_BREAKPOINT_C_R N13-AGTTTCATGTCATTGTGGTTAGAA<br>FH_BREAKPOINT_D_F N13-TGCAACATAATGCCTCAAAATC<br>FH_BREAKPOINT_D_R N13-CAATTCAGAAATGGAAAGTTACAA
5	*KMT2A*	PCR-Sanger (clinical)	Breakpoint 1 (NGS-4665)<br>Forward primer CCTCCTCTTGTACCTTGGCC<br>Reverse primer TGAGGGGAGGTGTTTGTTGG<br><br>Breakpoint 2 (NGS-4790)<br>Forward primer CACAGTCTCCATTCCTTGCCA<br>Reverse primer TCTCCCATCCCAAAGCAACC<br><br>Primers had the M13 tag for sequencing<br>M13F GTAAAACGACGGCCAGT<br>M13R CAGGAAACAGCTATGAC
6	*SOX5*	PCR-Sanger (clinical)	SOX5 Breakpoint 1F AGTGTTTCGATCTGGAGGGC<br>SOX5 Breakpoint 1R ACCAGGCTAGGCAACATGAC<br>SOX5 Breakpoint 2F CAGAGCCGGGAATAGTCACC<br>SOX5 Breakpoint 2R TCCATTTGCCTATCACCTGAAGG<br><br>SOX5 inversion breakpoint pair<br>Breakpoint 1F AGTGTTTCGATCTGGAGGGC<br>Breakpoint 2F CAGAGCCGGGAATAGTCACC
9	*FBN1*	PCR-Sanger (clinical)	As described (Family 3 in PMID:36411030)
13	*PAFAH1B1*	PCR-Sanger (clinical)	PAFAH1B1_BP1a_F GGGCATCAAAGGTGGTAGTG<br>PAFAH1B1_BP1a_R AACAGGGAATTACCAGCAAAAA<br>PAFAH1B1_Inv1a_F AGCGACAAGCCCTGCTAATA<br>PAFAH1B1_Inv1a_R CTGGTGGGATTACTGGCTTT<br>PAFAH1B1_Inv2a_F CTCAGTGGGGAGTGCTAGAG<br>PAFAH1B1_Inv2a_R GGCATCAAAGGTGGTAGTGC
14	*KMT2B*	Digital droplet PCR (clinical)	KMT2B_ddIn1_F_V1_and_KMT2B_ddIn1_R_V1<br>GGAAAGGGCCTCTGGAAGTG CGAAAAGGATCGGCCCAAGA<br><br>KMT2B_dd12_F_V1_and_KMT2B_dd12_R_V1<br>TCTGCTGTGACCCATTCCAC GTCCACAGACGTGGCAGAAT<br><br>KMT2B_dd13_F_V1_and_KMT2B_dd13_R_V1<br>CATACCACCCGGCCTGTC AGCAAGTGGGTGAACCTCAT

17	*MSH2*	PCR and Sanger (clinical)	Primers described elsewhere (PMID: 26498247)
21	*KMT2E*	PCR-Sanger (research & clinical)	Forward ACATTTACGCTTGGAAATTAAAA<br>Reverse GCTCTCTGATAACTCCTTTCTCTGA.
22	*GLI3*	PCR-Sanger (research)	As described (Family 2 in PMID:36411030)
23	*GLI3*	PCR-Sanger (clinical)	As described (Family 1 in PMID:36411030)
26	*PHEX*	PCR-Sanger (research)	PHEX-INV-1F TCTCTCACAAAGGTCACAGTCA<br>PHEX-INV-2F AAGATATTGAGTTGACCCTGTAG<br>PHEX-INV-1R CCCATGAGCCCAAACCTTCT<br>PHEX-INV-2R ACTTTTGCCGTTAGAAGCCC
30	*EDA*	PCR-Sanger (clinical)	EDA Breakpoint 1F GGGGAAATCTACCTAGGCACC<br>EDA Breakpoint 1R AGAGTGGGCTCAAGCATGAC<br>EDA Breakpoint 2F AGAGGTTGGAGAGGGAGTGG<br>EDA Breakpoint 2R CTCAGTCCTCTTCTGCTGGC<br><br>EDA inversion 1 breakpoint pair<br>Breakpoint 1F GGGGAAATCTACCTAGGCACC<br>Breakpoint 2F AGAGGTTGGAGAGGGAGTGG
33	*MECP2*	PCR-Sanger (research)<br>Single breakpoint	MECP2_Aii TGCAAATAATTCTAAGCTGTCCC<br>MECP2_DF GCCACCCACAAGTCTCCTA
38	*AUTS2*	Nanopore WGS (service/research)	Methods and analysis pipeline to be described elsewhere
39	*CUL4B*	PCR-Sanger (clinical)	Inv1: chrX: 118274086 – 119664466 (Build37)- KIAA1210 (R) + CUL4B (R)<br><br>KIAA1210-int2R GGGGCACATGGAGTCCTTTC<br>CUL4B-int20R TGCTGACAGAGAAAAATCCTACAAAC<br><br>Inv2: chrX:119664465 – 123558976 (Build37)- - CUL4B (F) + TENM1 (F)<br><br>CUL4B-int20F TGCTGCAAAAAGGCCAAACTG<br>TENM1-int23F CTCACCCCAGTTGGAATGGC
40	*NF2*	PCR-Sanger validated (clinical) and PacBio HiFi data	Described elsewhere (PMID: 38302265)
43	*APC*	Karyotyping (clinical), PacBio data (service, via Genomics England), PCR-nanopore of clinically relevant breakpoints (research)	Karyotyping confirms translocation and used for cascade testing, PacBio data confirms conformation, PCR-nanopore of selected breakpoints:<br><br>Breakpoint 1<br>APC-EF CTCTCCAGTTTCATATATGCCCA<br>APC-CR CAGGAGCATGGTGTGAGC<br>Breakpoint 2<br>APC-XR AGAGACTAGTGGTACTACAGGGA<br>APC-FR CTGAAATTCCCTCTCTCTGCT<br><br>Notes: The first targeted breakpoint contained a 92bp product (chr5:112,769,884-112,769,975) from *APC*, followed by sequence chr5:111,639,990-111,640,288 from *STARD4-AS1*. The second product contained 173bp from chr5:116,946,043-116,946,215, followed by 137bp of the proceeding sequence for the first junction chr5:112,769,977-112,770,113 within *APC*.

Table S4: Rare Variants defining inversion haplotype. Ultra rare variants (<0.1% AF in 100kGP) across the *MSH2* locus that are shared by the probands in Families 16 and 17. Although a 6Mb region was interrogated (chr2:45,450,067-51,450,067), all shared rare variants lay within the same 3.2Mb region identified by analysis of common variants (Figure 3C). Genomic positions are based on GRCh38. *MANE isoform unless otherwise stated. AggV2, aggregate vcf file with AN=156,390 unless otherwise stated. †ENST00000644092.1, ‡AN=156388. §Allelic read depths (ref/alt) for the individual reported by Brennan *et al*[11] from genome sequencing data (150-bp paired-end sequencing on a NovaSeq6000) were consistent with heterozygosity at all 13 positions.

Chr2 position	Ref/Alt	AF in AggV2	gnomAD AF (v4.0.0)	rsID	Gene (region)*	HGVSc	Allelic depth for Australian individual§
47,459,403	A/G	0.0352%	0.0197%	rs915614489	*MSH2* (intron 8 of 15)	c.1387-3628A>G	20/28
47,629,158	G/A	0.0454%	0.0151%	rs755620092	*MSH2* (intron 17 of 19†)	c.*1243-3644G>A	9/13
47,892,894	A/G	0.0121%	0.0026%	rs776369167	*FBXO11* (intron 1 of 22)	c.232+12595T>C	25/23
48,202,772	G/A	0.0109%	0.0013%	rs767041723	intergenic	NA	19/30
48,383,404	C/T	0.0403%	0.0118%	rs771247708	intergenic	NA	15/22
48,595,107	T/C	0.0090%	0.0013%	rs1374545554	*STON1* (intron 3 of 3)	c.2134-121T>C	14/15
48,998,287	G/A	0.0019%	0.0020%	rs1351434493	*FSHR* (intron 4 of 9)	c.375-7650C>T	18/19
49,099,629	A/T	0.0083%‡	Absent	rs1670951031	*FSHR* (intron 1 of 9)	c.153-31339T>A	22/25
49,234,481	C/T	0.0109%	0.0013%	rs902839622	intergenic	NA	21/23
49,437,430	C/A	0.0032%	Absent	rs1669735567	intergenic	NA	23/30
50,014,470	T/C	0.0058%	Absent	NA	*NRXN1* (intron 21 of 22)	c.4128+38801A>G	24/31
50,037,384	C/T	0.0959%	0.0507%	rs761040510	*NRXN1* (intron 21 of 22)	c.4128+15887G>A	22/25
50,095,187	A/G	0.0058%	Absent	NA	*NRXN1* (intron 18 of 22)	c.3547-3693T>C	20/30

References

1. Rhees, J., Arnold, M., and Boland, C.R. (2014). Inversion of exons 1-7 of the MSH2 gene is a frequent cause of unexplained Lynch syndrome in one local population. Fam Cancer *13*, 219-225. 10.1007/s10689-013-9688-x.
2. Moore, A.R., Yu, J., Pei, Y., Cheng, E.W.Y., Taylor Tavares, A.L., Walker, W.T., Thomas, N.S., Kamath, A., Ibitoye, R., Josifova, D., et al. (2023). Use of genome sequencing to hunt for cryptic second-hit variants: analysis of 31 cases recruited to the 100 000 Genomes Project. J Med Genet. 10.1136/jmg-2023-109362.
3. Loftus, S.K., Lundh, L., Watkins-Chow, D.E., Baxter, L.L., Pairo-Castineira, E., Nisc Comparative Sequencing, P., Jackson, I.J., Oetting, W.S., Pavan, W.J., and Adams, D.R. (2021). A custom capture sequence approach for oculocutaneous albinism identifies structural variant alleles at the OCA2 locus. Hum Mutat *42*, 1239-1253. 10.1002/humu.24257.
4. Shirts, B.H., Salipante, S.J., Casadei, S., Ryan, S., Martin, J., Jacobson, A., Vlaskin, T., Koehler, K., Livingston, R.J., King, M.C., et al. (2014). Deep sequencing with intronic capture enables identification of an APC exon 10 inversion in a patient with polyposis. Genet Med *16*, 783-786. 10.1038/gim.2014.30.
5. Xu, L., Wang, X., Lu, X., Liang, F., Liu, Z., Zhang, H., Li, X., Tian, S., Wang, L., and Wang, Z. (2023). Long-read sequencing identifies novel structural variations in colorectal cancer. PLoS Genet *19*, e1010514. 10.1371/journal.pgen.1010514.
6. Su, L.K., Steinbach, G., Sawyer, J.C., Hindi, M., Ward, P.A., and Lynch, P.M. (2000). Genomic rearrangements of the APC tumor-suppressor gene in familial adenomatous polyposis. Hum Genet *106*, 101-107. 10.1007/s004399900195.
7. Nakamura, W., Hirata, M., Oda, S., Chiba, K., Okada, A., Mateos, R.N., Sugawa, M., Iida, N., Ushiama, M., Tanabe, N., et al. (2024). Assessing the efficacy of target adaptive sampling long-

read sequencing through hereditary cancer patient genomes. NPJ Genom Med *9*, 11. 10.1038/s41525-024-00394-z.
8. Bozsik, A., Butz, H., Grolmusz, V.K., Polgar, C., Patocs, A., and Papp, J. (2023). Genome sequencing-based discovery of a novel deep intronic APC pathogenic variant causing exonization. Eur J Hum Genet *31*, 841-845. 10.1038/s41431-023-01322-y.
9. Weisschuh, N., Mazzola, P., Zuleger, T., Schaeferhoff, K., Kuhlewein, L., Kortum, F., Witt, D., Liebmann, A., Falb, R., Pohl, L., et al. (2023). Diagnostic genome sequencing improves diagnostic yield: a prospective single-centre study in 1000 patients with inherited eye diseases. J Med Genet. 10.1136/jmg-2023-109470.
10. Horton, A.E., Lunke, S., Sadedin, S., Fennell, A.P., and Stark, Z. (2023). Elusive variants in autosomal recessive disease: how can we improve timely diagnosis? Eur J Hum Genet *31*, 371-374. 10.1038/s41431-023-01293-0.
11. Brennan, B., Hemmings, C.T., Clark, I., Yip, D., Fadia, M., and Taupin, D.R. (2017). Universal molecular screening does not effectively detect Lynch syndrome in clinical practice. Therap Adv Gastroenterol *10*, 361-371. 10.1177/1756283X17690990.
